# Development of hydrophobic tag purifying monophosphorylated RNA for chemical synthesis of capped mRNA and enzymatic synthesis of circular mRNA

**DOI:** 10.1093/nar/gkae847

**Published:** 2024-10-17

**Authors:** Mami Ototake, Masahito Inagaki, Seigo Kimura, Kaoru Onda, Mizuki Tada, Daisuke Kawaguchi, Hirotaka Murase, Kosuke Fukuchi, Yinuo Gao, Kengo Kokubo, Susit Acharyya, Zheyu Meng, Tatsuma Ishida, Tairin Kawasaki, Naoko Abe, Fumitaka Hashiya, Yasuaki Kimura, Hiroshi Abe

**Affiliations:** Department of Chemistry, Graduate School of Science, Nagoya University, Furo-cho, Chikusa-ku, Nagoya, Aichi 464-8602, Japan; Department of Chemistry, Graduate School of Science, Nagoya University, Furo-cho, Chikusa-ku, Nagoya, Aichi 464-8602, Japan; Integrated Research Consortium on Chemical Sciences (IRCCS), Nagoya University, Furo-cho, Chikusa-ku, Nagoya, Aichi 464-8601, Japan; Department of Chemistry, Graduate School of Science, Nagoya University, Furo-cho, Chikusa-ku, Nagoya, Aichi 464-8602, Japan; Department of Chemistry, Graduate School of Science, Nagoya University, Furo-cho, Chikusa-ku, Nagoya, Aichi 464-8602, Japan; Department of Chemistry, Graduate School of Science, Nagoya University, Furo-cho, Chikusa-ku, Nagoya, Aichi 464-8602, Japan; Department of Chemistry, Graduate School of Science, Nagoya University, Furo-cho, Chikusa-ku, Nagoya, Aichi 464-8602, Japan; Department of Chemistry, Graduate School of Science, Nagoya University, Furo-cho, Chikusa-ku, Nagoya, Aichi 464-8602, Japan; Department of Chemistry, Graduate School of Science, Nagoya University, Furo-cho, Chikusa-ku, Nagoya, Aichi 464-8602, Japan; Department of Chemistry, Graduate School of Science, Nagoya University, Furo-cho, Chikusa-ku, Nagoya, Aichi 464-8602, Japan; Department of Chemistry, Graduate School of Science, Nagoya University, Furo-cho, Chikusa-ku, Nagoya, Aichi 464-8602, Japan; Department of Chemistry, Graduate School of Science, Nagoya University, Furo-cho, Chikusa-ku, Nagoya, Aichi 464-8602, Japan; Department of Chemistry, Graduate School of Science, Nagoya University, Furo-cho, Chikusa-ku, Nagoya, Aichi 464-8602, Japan; Department of Chemistry, Graduate School of Science, Nagoya University, Furo-cho, Chikusa-ku, Nagoya, Aichi 464-8602, Japan; Department of Chemistry, Graduate School of Science, Nagoya University, Furo-cho, Chikusa-ku, Nagoya, Aichi 464-8602, Japan; Research Center for Materials Science, Nagoya University, Furo-cho, Chikusa-ku, Nagoya, Aichi 464-8602, Japan; CREST, Japan Science and Technology Agency. 7 Gobancho, Chiyoda-ku, Tokyo 102-0076, Japan; Department of Chemistry, Graduate School of Science, Nagoya University, Furo-cho, Chikusa-ku, Nagoya, Aichi 464-8602, Japan; Department of Chemistry, Graduate School of Science, Nagoya University, Furo-cho, Chikusa-ku, Nagoya, Aichi 464-8602, Japan; CREST, Japan Science and Technology Agency. 7 Gobancho, Chiyoda-ku, Tokyo 102-0076, Japan; Institute for Glyco-core Research (iGCORE), Nagoya University, Furo-cho, Chikusa-ku, Nagoya, Aichi 464-8601, Japan

## Abstract

We developed phosphorylation reagents with a nitrobenzyl hydrophobic tag and used them for 5′-phosphorylation of chemically or transcriptionally synthesized RNA. The capability of hydrophobic tags to synthesize 5′-monophosphorylated RNA was evaluated based on the yield of the desired oligonucleotides, stability of protecting groups during cleavage/deprotection, separation ability in reverse-phase HPLC (RP-HPLC), and deprotection efficiency after RP-HPLC purification. The results showed that a nitrobenzyl derivative with a *tert*-butyl group at the benzyl position was most suitable for RNA 5′-phosphorylation. Using the developed phosphorylation reagent, we chemically synthesized 5′-phosphorylated RNA and confirmed that it could be purified by RP-HPLC and the following deprotection. In addition, we demonstrated complete chemical synthesis of minimal mRNA by chemical capping of 5′-monophosphorylated RNA. Ribonucleoside 5′-monophosphates with hydrophobic protecting groups have also been developed and used as substrates to transcriptionally synthesize 5′-phosphorylated RNA with >1000 bases. From the mixture of the by-products and the desired RNA, only 5′-monophosphorylated RNA could be effectively isolated by RP-HPLC. Furthermore, monophosphorylated RNA can be converted into circular mRNA via RNA ligase-mediated cyclization. Circular mRNA expression of nanoluciferase in cultured cells and mice. These techniques are important for the production of chemically synthesized mRNA and circular mRNA.

## Graphical Abstract

**Graphical Abstract:**
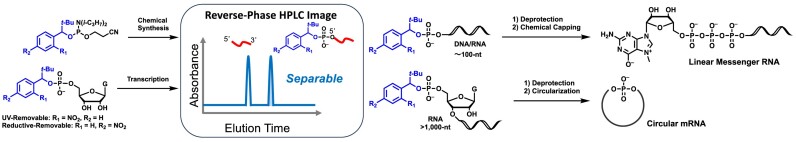

## Introduction

**Figure 1.**
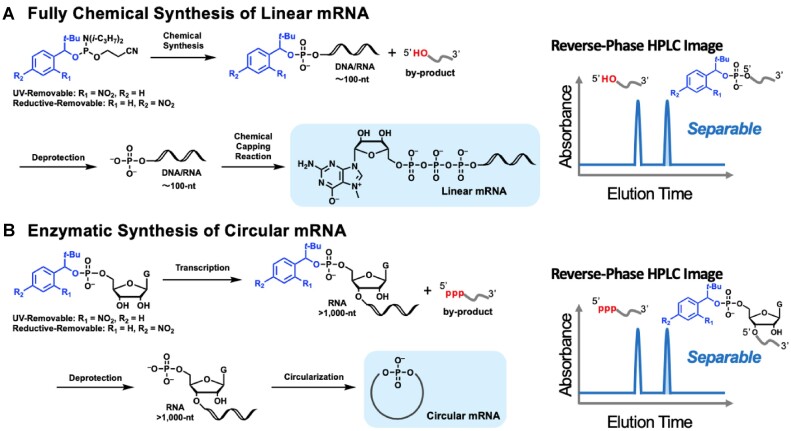
Strategy for synthesizing (**A**) chemically synthesized linear mRNA by a chemical capping reaction and (**B**) circular mRNA by enzymatic circularization reaction, starting from purified 5′-monophosphate RNA based on RP-HPLC purification using hydrophobic tags.

The therapeutic application of mRNA vaccines against the novel coronavirus disease (COVID-19) that emerged in 2019 has sparked increased interest in the development of new drugs using mRNA (1). Research and development are underway to enhance the functionality of mRNA. Linear RNA with a 5′ cap structure, produced by *in vitro* transcription, is considered the gold standard for mRNA (2). However, fully chemically synthesized minimal mRNA, aimed at precisely introducing chemical modifications, has been developed and shown to have improved translational activity compared to transcriptionally synthesized mRNA (Figure 1A) (3–5). This method demonstrated that it is possible to synthesize capped RNA by introducing a cap structure to chemically synthesized monophosphorylated RNA. Furthermore, circular RNA has been shown to have higher translation persistence and reduced excess immune responses compared to linear RNA (Figure 1B) (6–9). Research aimed at improving the functionality of circular RNA is progressing, with notable reports of significant enhancements in the translational activity of circular mRNA through improvements in the internal ribosome entry site (IRES) (6,10–13). Circular RNAs were synthesized through cyclization reactions using linear RNA as a precursor. To date, syntheses that utilize cellular splicing mechanisms (14), cyclization reactions with ribozymes (15), and enzymatic ligation have been developed (16). In particular, cyclization reactions by RNA ligase using 5′-monophosphate RNA as a precursor have been widely studied because of their versatility (14,17,18). Thus, 5′-monophosphate RNA serves as an important precursor for the chemical synthesis of minimal mRNA and the production of circular RNA, making the development of methodologies for synthesizing long-chain monophosphorylated RNA of high purity of significant importance.

**Figure 2.**
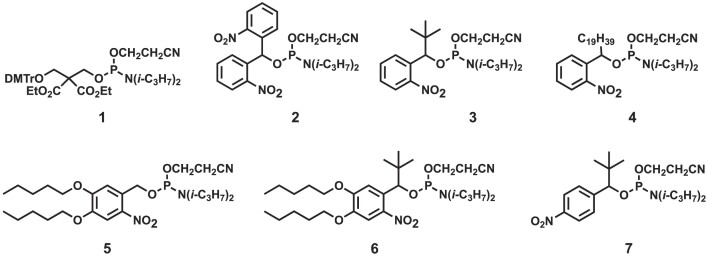
Structures of chemical phosphorylation reagents **1–7**.

A commercially available chemical phosphorylation reagent (CPR (**1**), Figure 2), a phosphoramidite compound, is widely used in the production of monophosphorylated DNA using an automated oligonucleotide synthesizer (19). CPR (**1**) contains a highly hydrophobic 4,4′-dimethoxytrityl group (DMTr), which can be used as a hydrophobic tag to purify 5′-monophosphate oligonucleotides by reverse-phase HPLC (RP-HPLC) (20). When synthesizing 5′-phosphorylated DNA using CPR (**1**), it is possible to obtain 5′-phosphorylated DNA after RP-HPLC purification followed by deprotection processes, including DMTr removal and basic deprotection (21). However, this method cannot be applied to RNA synthesis, because RNA is degraded during the basic deprotection process after DMTr removal. In particular, the synthesis of high-purity 5′-phosphorylated forms of long RNA sequences of >100 nucleotides is challenging. Pradère et al. developed a bis (2-nitrophenyl)methyl phosphoramidite (**2**) with a hydrophobic tag that can be removed by ultraviolet (UV) light and demonstrated its potential for purification by RP-HPLC (22). They succeeded in synthesizing microRNAs and small nuclear RNA by ligating chemically synthesized 5′-phosphorylated RNA.

The use of transcription reaction is practical for the synthesis of monophosphorylated RNA of approximately 150 bases or longer. When monophosphorylated RNA is synthesized by transcription reactions, guanosine 5′-monophosphate is added to the reaction at a certain ratio to nucleoside 5′-triphosphates (23). However, the product is a mixture of 5′-monophosphate RNA and 5′-triphosphate RNA, which makes analysis and purification difficult because of the similar molecular property. This mixture also affects the efficiency of cyclization reactions. Developing a technique to purify only monophosphorylated RNA from the transcription reaction could improve the efficiency of cyclization reactions.

In this study, we developed a new technique for the isolation and purification of 5′-monophosphorylated RNA from chemically and transcriptionally synthesized RNA. This technique uses hydrophobic tags that can be removed by UV irradiation (24–26) or reductive treatment (Figure 1) (27–29). The 5′-monophosphorylated RNA obtained using this method can be applied to the complete chemical synthesis of mRNA by a chemical capping reaction (Figure 1A) (3). In addition, using guanosine monophosphate derivatives with this hydrophobic tag, we synthesized and purified transcriptionally synthesized 5′-monophosphorylated RNA and successfully used it to synthesize circular mRNA (Figure 1B). The biological activities of chemically synthesized mRNA and circular mRNA were evaluated in cultured cells and mice.

## Materials and methods

### General information

All starting materials, reagents, and solvents of guaranteed grade were purchased from FUJIFILM-Wako Chemicals, Tokyo Chemicals, Sigma-Aldrich or Kanto Chemicals, and used without further purification. All reactions involving moisture-sensitive reagents were performed under argon atmosphere using oven-dried glassware. Column chromatography was performed on silica gel (63–210 mesh) purchased from Kanto Chemicals. All solid-phase oligonucleotide synthesis reagents were purchased from ChemGenes or Glen Research. All solvent compositions are reported in volume % unless specified otherwise. Oligonucleotides were synthesized using the DNA/RNA synthesizer NR-2A_7MX or NRs-4A_10R7NP (Nihon Techno Service). NMR spectra were obtained on JOEL NMR-ECS 400 (400 MHz for ^1^H NMR, 101 MHz for ^13^C NMR, and 163 MHz for ^31^P NMR) and JOEL NMR-ECS 600 (600 MHz for ^1^H NMR, 151 MHz for ^13^C NMR, and 243 MHz for ^31^P NMR) instruments. The ^1^H and ^13^C{^1^H} NMR chemical shifts (δ) are reported in parts per million (ppm) relative to residual solvents: CHCl_3_ (7.26 ppm for ^1^H NMR, 77.16 ppm for ^13^C NMR), CD_3_OD (3.31 ppm for ^1^H NMR, 49.00 ppm for ^13^C NMR), DMSO-*d*_6_ (2.50 ppm for ^1^H NMR, 39.52 ppm for ^13^C NMR), and D_2_O (4.79 ppm for ^1^H NMR) (42). The purity of the DNA and RNA was confirmed by denaturing polyacrylamide gel electrophoresis (dPAGE) containing 7.5 M urea as a denaturant (acrylamide:*N*,*N*'-methylenebis (acrylamide) = 19:1, electrophoresis buffer: 1× TBE, band visualization by SYBR Green II). The ESI-TOF mass spectra were obtained using a micro TOF-QII instrument (Bruker Daltonics). MALDI-TOF mass spectra were obtained using an UltrafleXtreme (Bruker Daltonics) with 3-hydroxypicolinic acid as a matrix to detect the peaks of the synthesized RNAs. LCMS analyses of the synthesized oligonucleotides were performed using an Agilent 1290 Infinity II—6530 LC/Q-TOF system and Waters ACQUITY H-Class PLUS_LBNW—Xevo G2-XS Qtof System_NQTW.

### The organic synthesis initial stages

The initial stages of organic synthesis are described in detail in the Supplementary Material.

### General synthetic procedure of phosphoramidites

Nitrobenzyl alcohols (4.8 mmol) and triethylamine (38 mmol) were dissolved in dry THF (19 ml). Cyanoethyl-*N*,*N*-diisopropylchlorophosphoramidite (11 mmol) was added to the solution and stirred at room temperature for 4 h. After the starting material disappeared on TLC, the reaction was stopped and 50 ml of NaHCO_3_ was added to the mixture. The mixture was extracted with ethyl acetate and washed 2-times with brine. The organic layer was dried over Na_2_SO_4_ and concentrated. The residue was purified by silica gel column chromatography on a neutral silica gel column using an eluent gradient of (hexane/EtOAc = 3/1 + 1% triethylamine) to afford the target compound. Fractions containing the target product were collected, evaporated, and dehydrated under vacuum to obtain the corresponding phosphoramidites.

### Compound 3

Yield **3** 83.0%, ^1^H-NMR (400 MHz, CDCl_3_) *δ* 7.70–7.77 (m, 2H, Ph-CH), 7.51–7.61 (m, 1H, Ph-CH), 7.36–7.41 (m, 1H, Ph-CH), 5.40–5.45 (m, 1H, CH of benzyl), 3.49–3.99 (m, 4H, –OCH_2_CH_2_CN), 2.62–2.76 (m, 1H, CH of *i*-Pr), 2.17–2.36 (m, 1H, CH of *i*-Pr), 1.27 (d, *J* = 7.0 Hz, 2H, CH_3_ of *i*-Pr), 1.16 (dd, *J* = 10.3, 6.7 Hz, 6H, CH_3_ of *i*-Pr), 0.99 (d, *J* = 6.7 Hz, 4H, CH_3_ of *i*-Pr), 0.87 (d, *J* = 3.8 Hz, 9H, CH_3_ of *t*-Bu); ^13^C-NMR (101 MHz, CDCl_3_) *δ* 149.78, 149.44, 135.62, 135.51, 131.78, 131.68, 131.07, 130.86, 128.09, 127.87, 123.86, 123.59, 117.75, 77.36, 77.21, 75.83, 75.71, 58.99, 58.76, 58.14, 57.91, 43.57, 43.42, 43.29, 37.23, 37.18, 37.12, 37.05, 25.89, 25.77, 24.83, 24.78, 24.73, 24.70, 24.67, 24.34, 24.27, 20.59, 20.53, 20.17, 20.10; ^31^P-NMR (162 MHz, CDCl_3_) *δ* 152.95, 148.78; HR-ESI-MS (*m*/*z*) calcd. for C_20_H_32_N_3_NaO_4_P [M + Na]^+^ 432.2028, found 432.1818.

### Compound 7

Yield **7** 82.8%, ^1^H-NMR (400 MHz, CDCl_3_) δ 8.14–8.19 (m, 2H, Ph-CH), 7.43–7.49 (m, 2H, Ph-CH), 4.52–4.56 (m, 1H, CH of benzyl), 3.42–3.91 (m, 4H, –OCH_2_CH_2_CN), 2.19–2.68 (m, 2H, CH of *i*-Pr), 0.98–1.27 (m, 12H, CH_3_ of *i*-Pr), 0.91 (d, *J* = 6.8 Hz, 9H, CH_3_ of *t*-Bu), ^13^C-NMR (101 MHz, CDCl_3_) δ 148.77, 148.53, 147.26, 147.11, 129.26, 129.16, 122.65, 122.50, 117.77, 117.50, 84.38, 84.24, 83.01, 82.89, 58.33, 58.12, 57.80, 57.58, 43.44, 43.31, 43.27, 43.15, 36.34, 36.29, 36.20, 26.18, 26.08, 24.81, 24.71, 24.64, 24.42, 24.34, 20.63, 20.55, 20.30, 20.23, ^31^P-NMR (162 MHz, CDCl_3_) δ 152.18, 147.97. ESI-TOF-MS calcd. for C_20_H_32_N_3_NaO_4_P^+^, 432.2023 [M + Na]^+^; found 432.2037.

### General synthetic procedure of RpGs

Compound **3** or **7** (1.08 mg, 2.63 mmol), compound **13** (690 mg, 1.58 mmol), and Molecular Sieves 3A (2.00 g) were suspended in 15.0 ml acetonitrile. After stirring at room temperature for 20 min, 1*H*-tetrazole (373 mg, 3.95 mmol) was added and the solution was stirred for 4 h. Subsequently, 5 M TBHP in decane (0.789 ml, 3.95 mmol) was added to the reaction mixture. After stirring at room temperature for 1 h, the reaction mixture was quenched by adding Milli-Q water and extracted with dichloromethane. The organic layer was washed with water and brine and dried over Na_2_SO_4_. The filtrate was evaporated *in vacuo* and purified by column chromatography on silica gel (dichloromethane/methanol = 99/1 to 10/1) to obtain compound **14** or **15** (846 mg, 1.11 mmol, 42.2%) as a yellow solid. Compounds **14** and **15** (846 mg, 1.11 mmol) were dissolved in MeOH (11 ml), and 28% NH_3_ aq. (11 ml) was added to the solution. The mixture was then stirred for 16 h at 55°C. The solvent was evaporated *under vacuum* and dissolved in water. The solution was washed with CH_2_Cl_2_. The aqueous layer was purified on a DEAE-Sephadex TM A-25 column (particle size: 40–100 μm, column size: φ = 4.5 cm, *h* = 8.4 cm (140 cm^3^), flow rate: 12 ml/min, solvent A: miliQ-water, solvent B: 1.5 M TEAB buffer + 10% acetonitrile, gradient: 0–40%B over 180 minutes). After purification, fractions containing the desired compounds were purified using a Wakosil^®^ 25C18 column (particle size: 15–30 μm (spherical), column size: φ = 4.80 cm, *h* = 10.2 cm (184 cm^3^), flow rate: 12 ml/min, solvent A: 50 mM TEAA + 5% acetonitrile, solvent B: acetonitrile, gradient: 0–90% over 100 minutes). After purification, the solution was evaporated *in vacuo* to obtain the desired compound as a yellow solid (counter cation: triethylammonium). The compound was dissolved in 2 ml MeOH, and 40 ml of 0.14 M NaClO_4_ in acetone was added and centrifuged at 4500 rpm for 10 min. This procedure was repeated three times. Compounds ***Compound 16***

Yield **16** 72.3%, ^1^H-NMR (600 MHz, DMSO-D6) δ 10.66 (s, 1H, NH), 7.89–7.43 (m, 5H, Ph-CH and CH-8), 6.65 (2s, 2H, NH_2_), 5.64 (d, *J* = 9.2 Hz, 1H, CH of *t*-Bu), 5.59 (dd, *J* = 5.9, 1.2 Hz, 1H, CH-1), 5.45 (m, 1H, OH-2′), 5.34 (t, *J* = 5.3 Hz, 1H, OH-3′), 4.37 (m, 1H, CH-2′), 3.97 (m, 1H, CH-3′), 3.72 (m, 1H, CH-4′), 3.64–3.45 (m, 2H, CH_2_-5′), 0.80 (s, 9H, CH_3_ of *t*-Bu) ppm. ^13^C-NMR (151 MHz, DMSO-D_6_) δ 207.1, 157.4, 154.2, 151.9, 149.3, 136.4, 136.2, 132.5, 130.9, 128.3, 124.1, 117.1, 87.0, 76.3, 74.2, 74.1, 71.4, 71.3, 64.8, 36.9, 31.2, 26.3 ppm. ^31^P-NMR (243 MHz, DMSO-D_6_) δ −0.2, −0.3, −0.4 ppm. HRMS (ESI) *m/z* calculated for [M−H]^−^: 553.1454, found for [M−H]^−^: 553.1481.

### Compound 17

Yield **17** 72.3%, ^1^H-NMR (600 MHz, DMSO-D6) δ 10.72 (s, 1H, NH), 8.15–8.08 (m, 2H, Ph-CH), 7.91–7.86 (m, 1H, CH-8), 7.52 (dd, *J* = 11.6, 8.8 Hz, 2H,, Ph-CH), 6.60 (s, 2H, NH_2_ of 2), 5.61 (m, 1H, OH-2′), 5.38–5.28 (m, 2H, OH-3′and CH of *t*-Bu), 4.86 (dd, *J* = 9.3, 3.2 Hz, 1H, CH-1′), 4.39–4.32 (m, 1H, CH-2′), 3.88 (m, 1H, CH-3′), 3.79–3.58 (m, 3H, CH-4′, CH_2_-5′), 0.84 (d, *J* = 4.4 Hz, 9H, CH of *t*-Bu) ppm. ^13^C-NMR (151 MHz, DMSO-D_6_) δ 207.1, 157.4, 154.2, 151.9, 149.3, 136.4, 136.2, 132.5, 130.9, 128.3, 124.1, 117.1, 87.0, 76.3, 74.2, 74.1, 71.4, 71.3, 64.8, 36.9, 31.2, 26.3 ppm. ^31^P-NMR (243 MHz, DMSO- D_6_) δ −0.2, −0.3, −0.4 ppm. HRMS (ESI) *m/z* calculated for [M−H] ^−^: 553.1454, found for [M−H] ^−^: 553.1484.

### Solid-phase DNA synthesis

DNAs with hydrophobic protecting groups were synthesized on a DNA/RNA synthesizer NR-2A_7MX or NRs-4A_10R7NP (Nihon Techno Service) using DNA phosphoramidites and CPG (Chemgenes; deoxyguanosine (n-ibu) 3′-lcaa CPG, 1000A T.V. 47.3 μmol/g) and CPR**1–7**. 50 mM DNA phosphoramidite and 100 mM CPR**1–7** solution in acetonitrile were prepared for use in the DNA synthesis. Reagents for the synthesizer were used as follows: 3 w/v% trichloroacetic acid in dichloromethane for deblocking; 0.25 M 5-benzylthio-1*H*-tetrazole in acetonitrile (Wako) for coupling; a mixture of acetic anhydride/ tetrahydrofuran/ pyridine (1:8:1, Wako) and 10 (v/v)% 1-methylimidazole in tetrahydrofuran (Wako) for capping; 0.01 M iodine in 64% acetonitrile, 6% pyridine, 30% water for oxidation (Honeywell). After synthesis, DNAs was cleaved from the support and deprotected using a 1:1 mixture of 40% aqueous methylamine-28% ammonium hydroxide at 65°C for 15 min or 28% ammonium hydroxide at room temperature overnight. After cleavage and deprotection, the CPG was removed by filtration, and the filtrate was concentrated. Crude DNAs was purified using reversed-phase HPLC. The purification conditions were as follows: column, YMC Hydrosphere C18, 250 × 10 mm I.D., S-5 μm, 12 nm; Solution A, 50 mM triethylammonium acetate (pH 7.0) containing 5% acetonitrile; Solution B, acetonitrile; typical gradient, 0–100% Solution B over 20 min; column temperature, 50°C; flow rate, 1 ml/min; detection wavelength, 260 nm. After purification, the DNA was precipitated by isopropanol precipitation (DNA solution: 1.00 ml, 3 M NaOAc aq.: 125 μl, isopropanol: 1.25 ml, 20 mg/ml glycogen aqueous solution: 12.5 μl). The mixture was cooled to –80°C for 30 min and centrifuged (15 000 rpm, 4°C, 15 min). The supernatant was removed, and 80% aqueous ethanol (1.0 ml) was added to the pellet, and centrifuged (15 000 rpm, 4°C, 15 min). The supernatant was removed, and the pellet was dried under reduced pressure. The resulting white solid was dissolved in water. The DNA concentration was determined using the extinction coefficient calculated by the nearest-neighbor method using Oligoanalyzer software from Integrated DNA Technologies, based on the absorbance at 260 nm measured on a NanoDrop2000 spectrometer (Thermo Fisher Scientific) (43). DNA purity was confirmed by 20% denaturing polyacrylamide gel electrophoresis (dPAGE) containing 7.5 M urea as a denaturant and reverse-phase HPLC. For dPAGE analysis, the ratio of acrylamide to *N*,*N*'-methylenebis (acrylamide) was 19:1. After electrophoresis, the gel was stained with SYBR Green II (Lonza) and visualized using a ChemiDoc XRS Plus system (Bio-Rad).

### Solid-phase RNA synthesis

RNAs with nitrobenzyl tags were synthesized in the same manner as the DNA described above. Cleavage from the support and deprotection were performed by the treatment with a 1:1 mixture of 28% aqueous ammonia and 40% methylamine (1.00 ml) at 65°C for 15 min. After filtration with a membrane filter and centrifugal evaporation, the RNAs were treated with 1 M tetrabutylammonium fluoride in THF (1.0 ml) at room temperature overnight. The reaction mixture was quenched by the addition of 1 M Tris–HCl buffer (pH 7.5, 1.0 ml) and concentrated to remove the THF. The solution was desalted using an NAP25 column (Merck), and the RNA was precipitated by isopropanol precipitation (RNA solution: 1.00 ml, 3 M NaOAc aq.: 125 μl, isopropanol: 1.25 ml, 20 mg/ml glycogen aqueous solution: 12.5 μl). The mixture was cooled to –80°C for 30 minutes and centrifuged (15 000 rpm, 4°C, 15 min). The supernatant was removed, and 80% aqueous ethanol (1.0 ml) was added to the pellet, and centrifuged (15 000 rpm, 4°C, 15 min). The supernatant was removed, and the pellet was dried under reduced pressure. The resulting white solid was dissolved in water. Crude RNAs was purified by RP-HPLC. The purification conditions were as follows: column, YMC Triart Bio C4, 250 × 10 mm I.D., S-5 μm, 12 nm; Solution_A, 50 mM triethylammonium acetate (pH 7.0) containing 5% acetonitrile; Solution_B, acetonitrile; typical gradient, 0 to 20% Solution_B over 20 min; column temperature, 50°C; flow rate, 1 ml/min; detection wavelength, 260 nm. The RNA concentration and purity were confirmed in the same way as DNA described above.

### Deprotection of *o*-nitrobenzyl groups by UV-irradiation

5′-Nitrobenzylphosphate DNA/RNA solutions were added to a transparent 96-well multi-well plate (75 μl/well) and irradiated with 365 nm light at 4 mW/cm^2^ for 10 min by a MAX-305 light source device (Asahi spectroscopy). After deprotection, the DNA/RNA was precipitated by isopropanol precipitation (DNA/RNA solution: 1.00 ml, 3 M NaOAc aq.: 125 μl, isopropanol: 1.25 ml, 20 mg/ml glycogen aqueous solution: 12.5 μl). The mixture was cooled to –80°C for 30 min and centrifuged (15 000 rpm, 4°C, 15 min). The supernatant was removed, and 80% aqueous ethanol (1.0 ml) was added to the pellet, and centrifuged (15 000 rpm, 4°C, 15 min). The supernatant was removed, and the pellet was dried under reduced pressure. The resulting white solid was dissolved in water. DNA/RNA was analyzed using reverse-phase HPLC and dPAGE. HPLC condition was as follows: case of DNA, column, YMC Hydrosphere C18, 250 × 10 mm I.D., S-5 μm, 12 nm; Solution A, 50 mM triethylammonium acetate (pH 7.0) containing 5% acetonitrile; Solution B, acetonitrile; typical gradient, 0 to 60% Solution B over 20 min; column temperature, 50°C; flow rate, 1 ml/min; detection wavelength, 260 nm. In Case of RNA, column, YMC Triart Bio C4: Solvent A, 50 mM TEAA (pH 7.0), 5% ACN; Solvent B, ACN; Gradient 0–100% B/ 0–20 min; Flow rate, 1 ml/min; Column temperature, 50°C; Column size 250 × 4.6 mm I.D., S-5 μm, 12 nm; detection wavelength, 260 nm.

### Deprotection of o-/p-nitrobenzyl groups by reductive condition

For the deprotection of *o*-nitrobenzyl groups, the 10 μM 5′-nitrobenzylphosphate DNA/RNA solutions (1.0 nmol) in 20 mM Tris–HCl buffer (pH 8.0) containing 100 mM sodium dithionite were incubated at 37°C for 30 min and then 65–95°C for 10–20 min. The reaction time varies depending on the substituents of benzene position (see Table 2). For the deprotection of *p*-nitrobenzyl group, the 10 μM 5′-nitrobenzylphosphate DNA/RNA solutions (1.0 nmol) in 20 mM Tris–HCl buffer (pH 8.0) containing 100 mM sodium dithionite were incubated at 37°C for 30 min and then 55°C for 2 h. After deprotection, DNA/RNA were analyzed by reversed-phase HPLC. HPLC condition was as follows: In the case of 19 mer DNA, column: YMC hydrosphere C18: solvent A, 50 mM TEAA (pH 7.0), 5% acetonitrile; solvent B, ACN; gradient 0–60% B/ 0–20 min; flow rate, 1 ml/min; column temperature, 50°C; column size 250 × 4.6 mm I.D., S-5 μl, 12 nm; detection wavelength, 260 nm. In the case of transcribed RNA, column: YMC Triart Bio C4: solvent A, 100 mM TEAA (pH 7.0), 5% ACN; solvent B, 100 mM TEAA (pH 7.0), 50% ACN; gradient 15–30% B/ 0–25 min; flow rate, 1 ml/min; column temperature, 50°C; column size 250 × 4.6 mm I.D., S-5 μm, 30 nm; detection wavelength: 260 nm.

### Chemical synthesis of mRNA

5′-Phosphate RNA was synthesized using phosphoramidite **3** and purified by reverse-phase HPLC (column: hydrosphere C18 (250 × 4.6 mm I.D., S-5 μm, 12 nm), solvent A 50 mM TEAA buffer (pH 7.0) + 5% CH_3_CN, solvent B: CH_3_CN, gradient: 5–50% B over 20 min, column temperature 50°C, flow rate: 1 ml/min, detection: 254 nm, loop size: 2.0 ml). After HPLC purification, the 5′-nitrobenzylphosphate RNA solution was added to a transparent 96-well multi-well plate (75 μl/well) and irradiated with 365 nm light at 4 mW/cm^2^ for 10 min by a MAX-305 light source device (Asahi spectroscopy). After removal of the nitrobenzyl group, RNA was precipitated by isopropanol precipitation (RNA solution: 1.00 ml, 3 M NaOAc aq.: 125 μl, isopropanol: 1.25 ml, 20 mg/ml glycogen aqueous solution: 12.5 μl). The mixture was cooled to –80°C for 30 min and centrifuged (15 000 rpm, 4°C, 15 min). The supernatant was removed, and 80% aqueous ethanol (1.0 ml) was added to the pellet, and centrifuged (15 000 rpm, 4°C, 15 min). The supernatant was removed, and the pellet was dried under reduced pressure. The resulting white solid was dissolved in water. The RNA pellet was dissolved in water and quantified by NanoDrop. The RNA solution was lyophilized and dissolved in dry dimethyl sulfoxide (DMSO, 127 μl/5 nmol RNA). A 15 mM DMSO solution of 7-methylguanosine 5′-diphosphate-imidazolide (chemical capping reagent, 333 μl/5 nmol RNA) and 1-methylimidazole (40 μl/5 nmol RNA) was added to the RNA solution (3). The mixture was incubated at 55°C for 4 h, and then RNA was precipitated by isopropanol precipitation (reaction mixture: 500 μl, water: 500 μl, 3 M NaOAc aq.: 125 μl, isopropanol: 1.25 ml, 20 mg/ml glycogen aqueous solution: 12.5 μl). The mixture was cooled to –80°C for 30 min and centrifuged (15 000 rpm, 4°C, 15 min). The supernatant was removed, and 80% aqueous ethanol (1.0 ml) was added to the pellet, and centrifuged (15 000 rpm, 4°C, 15 min). The supernatant was removed, and the pellet was dried under reduced pressure. The resulting white solid was dissolved in water. The crude capped-RNA was purified by RP-HPLC (Column: Hydrosphere C18 (250 × 4.6 mm I.D., S-5 μm, 12 nm), Solvent A: 50 mM TEAA buffer (pH 7.0) + 5% CH_3_CN, Solvent B: CH_3_CN, Gradient: 5–50% B over 20 min, column temperature 50°C, flow rate: 1 ml/min, detection: 254 nm, loop size: 2.0 ml). Characterization of the product was carried out by dPAGE (20 × 20 cm gel, 30 W, 3 h, 2 pmol RNA sample loading, SYBR Green II Stain, Low range ssRNA Ladder (NEB) as a size marker), and LC–MS.

### WST assay

HeLa cells were cultured and maintained at 37°C and 5% CO_2_ in a humidified incubator in D-MEM medium containing 10% FBS. One day before transfection, the cells were trypsinized and seeded into a 96-multiwell cell culture plates at 6000 cells per well. The cells were then incubated at 37°C for 24 h in a 5% CO_2_ atmosphere. The mRNA transfection was performed using Lipofectamine messengerMAX. After incubation at 37°C for 24 h in a 5% CO_2_ atmosphere, the WST8 assay was performed according to general procedures (44).

### HiBiT expression in HeLa cells

HeLa cells were cultured and maintained at 37°C and 5% CO_2_ in a humidified incubator in D-MEM medium containing 10% FBS. One day before transfection, the cells were trypsinized and seeded into a 96-multiwell cell culture plate at 1 × 10^4^ cells per well. Immediately before transfection, the medium was replaced with Opti-MEM I (90 μl). To transfect RNA into HeLa cells, a mixture of RNA (20, 100 or 200 ng), Lipofectamine^®^ MessengerMAX (0.3 μl), and Opti-MEM I (10 μl) per well were added to the cells. After 4 h of incubation, the supernatant was removed and replaced with 100 μl D-MEM containing 10% FBS. Eight hours after transfection, the medium was removed from the wells, washed twice with 150 μl PBS, and the cells were lysed with 20 μl of Nano-Glo^®^ HiBiT Lytic Buffer containing LgBiT Protein solution (0.2 μl) and Nano-Glo^®^ HiBiT Lytic Substrate solution (0.4 μl), which were obtained using the Nano-Glo^®^ HiBiT Lytic Detection System (Promega) (35). After 5 min of incubation, the lysates were transferred to a white 96-multiwell plate and chemiluminescence was measured on a Mithras LB940 plate reader (Berthold).

### Preparation of LNP

LNPs were prepared by the vortex mixing method. Briefly, 10 μg of RNA was dissolved in 600 μl of 5 mM Citrate buffer (pH4). Lipids were dissolved in 200 μl of ethanol with the following lipid composition: SM102/DOPE/Cholesterol/DMG-PEG2k = 50/10/38.5/1.5 molar ratio (lipid/RNA = 400 nmol/10 μg). Under vortex mixing, the lipid ethanol solution was added into the buffer containing RNA, and then 1 ml of PBS was added. The LNP suspension was centrifuged (25°C, 1500 × g, 30 min) with an Amicon Ultra 100k. LNPs were recovered from the tubes with PBS.

### HiBiT-expression in mice

LNP-formulated mRNAs were injected into C57BL6N-5w-female mice (10 μg/mouse) via the tail vein. Six hours after the injection, the liver and the spleen were collected. These tissues were homogenized using a Micro Smash MS-100R (TOMY, Japan) in 1 ml of lysis buffer (100 mM Tris–HCl, 2 mM EDTA, 0.1% Triton X-100, pH = 7.8). The homogenate was centrifuged (15 000 rpm, 4°C, 10 min) and the supernatant was collected. HiBiT expression was measured using a HiBiT Lytic assay system (Promega). Briefly, a 30 μl of the supernatant was mixed with 60 μl of the solution containing LgBiT protein and the substrate, and the luminescence was measured using a luminometer (GloMax, Promega). The total protein amount in the supernatant was quantified by BCA assay. HiBiT expressions were expressed as RLU per mg of protein.

### Preparation of template DNA by PCR

The PCR mixture was consisted of 0.3 μM primers, 1 ng/μl pNL1.1TK vector (for circular mRNA without IRES) or EMCV-IRES-pNL1.1TK plasmid (for circular mRNA with IRES), 0.2 mM dNTPs, 1.5 mM MgSO_4_, 1 × PCR Buffer for KOD -Plus- Neo, 0.02 units/μl KOD -Plus- Neo (Toyobo). The mixture was subjected to the following thermal cycling conditions: 95°C for 2 min → (98°C for 10 s → 55°C for 30 s → 72°C for 1 min) × 30 cycles → 72°C for 5 min. The reaction was analyzed by 1% agarose gel electrophoresis (100 V, 30 min, 1× TAE). After the gel electrophoresis, The PCR product was purified using a wizard^®^ SV gel and PCR Clean-up System (Promega) according to the manufacturer's recommended protocol.

### *In vitro* transcription by using RpGs

The transcription solution was consisted of 20 ng/μl DNA template (PCR product), 10 mM DTT, 24 mM MgCl_2_, 7.5 mM ATP, CTP, UTP, 1.9 mM, GTP, 7.5 mM *o*-Nb-RpG (**16**) or *p*-Nb-RpG (**17**), 25 U/μl T7 RNA polymerase (house-made), 0.004 U/μl pyrophosphatase (New England Biolab, N2403L), 0.2 U/μl RNase inhibitor). The transcription solution was incubated at 37°C for 2 h, and then DNase I (Takara) was added to the transcript solution and incubated at 37°C for 30 min to remove any remaining DNA transcription template. Next, a 1:1 mixture of TE-saturated phenol and chloroform was added, and the mixture was centrifuged. Chloroform was then added to the water layer and centrifuged. The aqueous layer was collected and RNA was precipitated by isopropanol precipitation (RNA solution: 1.00 ml, 3 M NaOAc aq.: 125 μl, isopropanol: 1.25 ml, 20 mg/ml glycogen aqueous solution: 12.5 μl). The mixture was cooled to –80°C for 30 min and centrifuged (15 000 rpm, 4°C, 15 min). The supernatant was removed, and 80% aqueous ethanol (1.0 ml) was added to the pellet, and centrifuged (15 000 rpm, 4°C, 15 min). The supernatant was removed, and the pellet was dried under reduced pressure. The resulting white solid was dissolved in water. The RNA solution was purified using reverse-phase HPLC. The condition was as follows: YMC-Triart Bio C4: Solvent A, 100 mM TEAA (pH 7.0), 5% acetonitrile; solvent B, 100 mM TEAA (pH 7.0), 50% acetonitrile; gradient 15–30% B/ 0–20 min; flow rate, 1 ml/min; column temperature, 50°C; column size 250 × 4.6 I. D., S-5 μm, 30 nm; detection wavelength, 260 nm.

### Synthesis of circular mRNA

The annealing solution was consisted of 1.0 μM Splint DNA, 0.5 μM linear RNA, T4 RNA ligase 2 buffer (50 mM Tris–HCl, 2 mM MgCl₂, 1 mM DTT, 400 μM ATP). The solution was heated at 90°C for 3 min and then gradually cooled to room temperature. PEG 8000 was added to the solution (10% final concentration), followed by T4 RNA ligase 2 (house-made) at a final concentration (25 pg/μl final concentration). The mixture was then incubated at 37°C for 1 h. RNA products were collected using the LiCl precipitation technique. The RNA pellet was dissolved in water and subjected to 5% dPAGE, and The RNA bands were visualized by handy UV ramp. The circular RNA bands were cut and the gel pieces were immersed in water (1.0 ml). The gel pieces were thoroughly clashed and shaken at room temperature overnight to extract the circular mRNA. 3 M sodium acetate aq. (pH 5.2, 125 μl), isopropanol (1.25 ml), and 20 mg/ml glycogen aqueous solution (12.5 μl), were added to the extracted RNA solution to precipitate RNA (1.00 ml). The mixture was cooled to –80°C for 30 min and centrifuged (15 000 rpm, 4°C, 15 min). The supernatant was removed, and 80% aqueous ethanol (1.0 ml) was added to the pellet, and centrifuged (15 000 rpm, 4°C, 15 min). The supernatant was removed, and the pellet was dried under reduced pressure. The resulting white solid was dissolved in water and quantified by absorption at 260 nm using a NanoDrop spectrophotometer. The RNA solution was analyzed using 5% dPAGE (26 mA, 2 h, SYBR Green II staining). Gel images were obtained using a ChemiDoc (Bio-Rad).

### NanoLuc expression in HeLa cells

HeLa cells cultured in a dish were washed twice with 3 ml PBS. Cells were trypsinized and seeded into a 96-well cell culture plate at 10 × 10E4 cells per well. Thereafter, the cells were incubated for 24 h at 37°C in a 5% CO_2_ atmosphere. The medium was removed and the cells were washed once with 150 μl of PBS. Lipofectamine^®^ MessengerMAX transfection reagent (Invitrogen, 0.15 μl/well) and Opti-MEM (Thermo Fisher Scientific, 10 μl/well) were mixed, and 32.48 μl of each solution was added to 32 ng of mRNA. After adding 89 μl Opti-MEM to each well, 11.3 μl of the prepared mRNA/Lipofectamine mixture was added to each well. After incubating for 3 h at 37°C in a 5% CO_2_ atmosphere, the supernatant was removed, and the medium was replaced with DMEM containing 10% feral bovine serum. 24 h after transfection, the medium was removed from the 96-well plate and washed once with 150 μl PBS. Luminescence measurements were performed using a NanoGlo Luciferase Assay System (Promega). The measurements were performed using a plate reader (Berthold).

### NanoLuc expression in mice

LNP-formulated circular mRNAs were injected into ICR45w-female mice (0.25 or 0.05 mg/kg) from the tail vein. 24 h after the injection, the liver and the spleen were collected. Tissue lysate was prepared as in the previous section for ‘HiBiT-expression in mice’. NanoLuc expression was measured using NanoGlo Luciferase Assay System (Promega). Briefly, a 20 μl of the lysate was mixed with 20 μl of the substrate solution, and the luminescence was measured using a luminometer (GloMax, Promega). The total protein amount in the lysate was quantified by BCA assay. NLuc expressions were expressed as RLU per mg of protein.

### Statistical analysis

Pair-wise comparisons between treatments were made using a two-tailed Student *t*-test. For comparisons among three or more groups, the one-way analysis of variance (ANOVA), followed by Bonferroni or Tukey–Kramer test, was used. A *P*-value of <0.05 was considered to be significant (*P< 0.05, **P< 0.001, ****P< 0.0001).

## Results and discussion

### Synthesis of chemical phosphorylation reagents

In the initial stage of this study, we optimized hydrophobic tag phosphoramidites for chemical synthesis, in which a substituent was introduced at the benzyl position of the nitrobenzyl group (30) and alkyl ether structures were introduced at the meta and para positions (31) (Figure 2). These phosphoramidite reagents can be used as phosphorylation reagents in oligonucleotide synthesis. It is expected that the *o*-nitrobenzyl group can be removed by UV irradiation and the *p*-nitrobenzyl group can be removed under reductive conditions after oligonucleotide synthesis. To identify the optimal structure, we comprehensively evaluated the reaction efficiency of the phosphoramidites, stability of the nitrobenzyl hydrophobic tag during deprotection/cleavage of oligonucleotides under basic conditions, and the deprotection efficiency of the newly designed hydrophobic tags after RP-HPLC purification. These properties were compared with those of the known phosphoramidites **1** and **2**. The separation performance of these hydrophobic tags in RP-HPLC was evaluated in detail based on the differences in hydrophobicity. Specifically, compound **3** with a *tert*-butyl group was introduced at the benzyl position (25), compound **4** with a linear C19 alkyl group, compound **5** with pentyl ether structures introduced at the meta- and para-positions, compound **6** with a *tert*-butyl group introduced at the benzyl position in compound **5**, and *p*-nitrobenzyl derivative **7** was designed (Figure 2).

**Figure 3.**
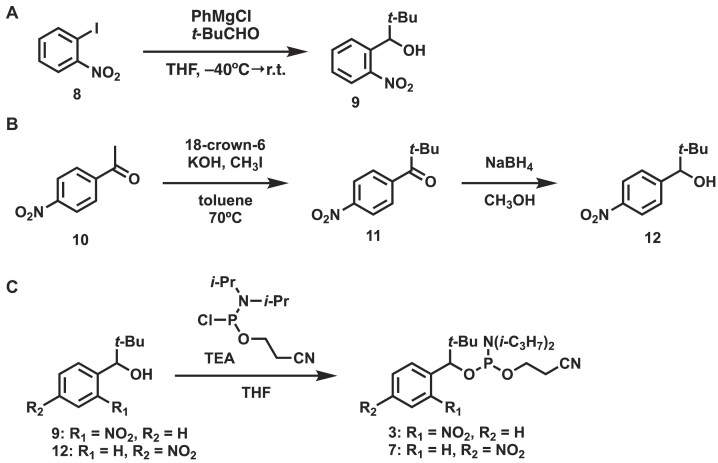
Synthesis of (**A, B**) nitrobenzyl Alcohols and (**C**) their phosphoramidite derivatives.

*o*-Nitrobenzyl alcohol (**9**) was synthesized from 1-iodo-2-nitrobenzene (**8**) and reacted with the corresponding aldehyde (65% yield; Figure 3A) (25). *p*-Nitrobenzyl alcohol was prepared from *p*-nitroacetophenone (**10**) in the presence of potassium hydroxide and iodomethane by methylation of the *α*-position with 18-crown-6 (53% yield). Ketone **11** was then reduced with sodium borohydride to afford *p*-nitrobenzyl alcohol **12** in 56% yield (Figure 3B). The resulting nitrobenzyl alcohols (**9** and **12**) were converted to the phosphoramidite form by reaction with 2-cyanoethyl-*N*,*N*-diisopropylchlorophosphoramidite in THF in the presence of triethylamine (**3**:83% yield, **7**:83% yield, Figure 3C).

### Chemical synthesis of 5′-monophosphate oligonucleotides

**Figure 4.**
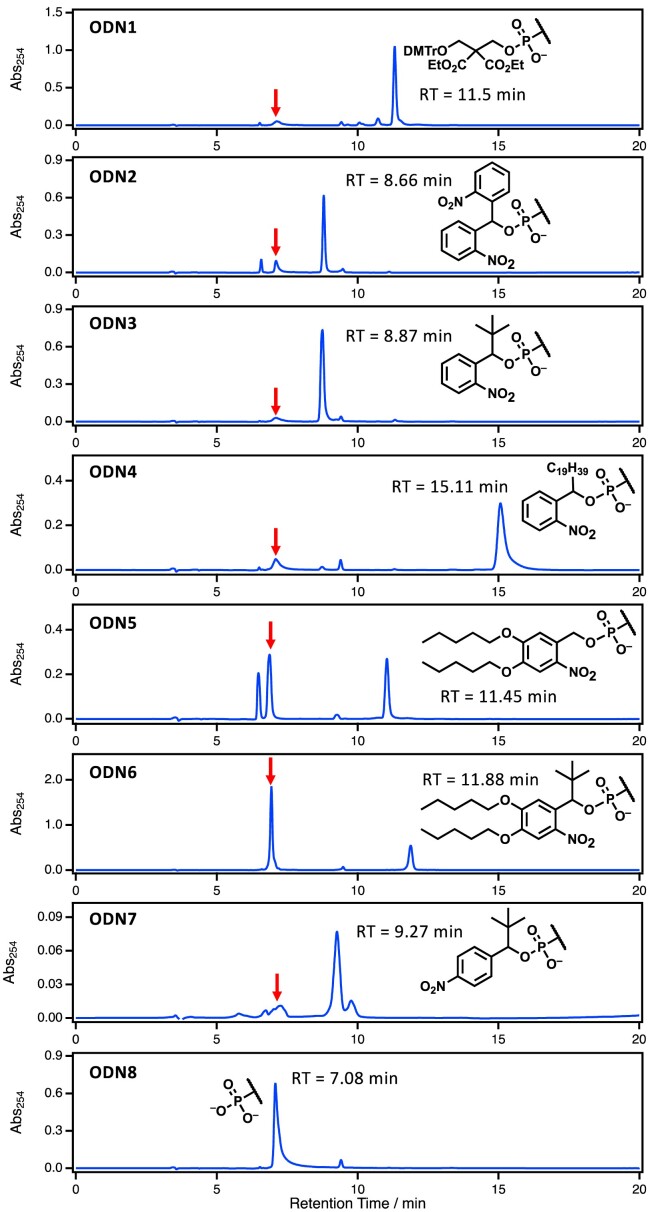
HPLC profiles of the synthesized **ODN1–8**. The red arrows indicate the peaks derived from 5′-phosphate-DNA produced by the hydrolysis of phosphate-protecting groups after the cleavage and deprotection of DNAs. Column: hydrosphere C18 (250 × 4.6 mm I.D., S-5 μm, 12 nm), solvent A 50 mM TEAA buffer (pH 7.0) + 5% CH_3_CN, solvent B: CH_3_CN, gradient: 0–100% B over 20 min, column temperature 50°C, flow rate: 1 ml/min, detection: 254 nm, loop size: 2.0 ml.

To evaluate the properties of the newly synthesized phosphorylation reagents, we synthesized 5′-monophosphorylated DNA using an automated oligonucleotide synthesizer following the general phosphoramidite chemistry. Specifically, we first synthesized 19-nucleotide DNA and phosphorylated it using commercially available CPR (**1**), previously reported bis (2-nitrophenyl)methyl phosphoramidite (**2**), and our newly developed phosphorylation reagents (**3–7**) (Table 1). The synthesized DNA was released from the solid support and deprotected by treatment with concentrated aqueous ammonia at 55°C for 16 h. This process yielded **ODNs 1–7**, each with different hydrophobic tag structures, which were subsequently analyzed and purified using RP-HPLC to isolate the target DNA. The results of the analyses of the mixtures before purification and DNA after purification are shown in (Figure 4 and Supplementary Figures S1–S9). All of the phosphorylation reagents tested produced the target, which was confirmed by MALDI-TOF-MS and LC–MS analyzes (Table 1, Supplementary Figures S1–S8). However, the hydrophobicity and stability of the tag in the phosphate group desired significantly in these reagents. As for the hydrophobicity, it was found that 5′-phosphate DNA bearing hydrophobic tags could be clearly separated from untagged DNAs by RP-HPLC. Fully-synthesized, tagged DNA showed significantly delayed retention times compared to DNA without hydrophobic tags (**ODN8**). In particular, **ODN4** showed the highest hydrophobicity owing to its long alkyl chain and was strongly retained on the column (retention time = 15.1 min). **ODN3** showed a separation performance comparable to that of **ODN2**, which was synthesized using a previously reported phosphorylation reagent (**2**) (**ODN2**: 8.66 min, **ODN3**: 8.87 min). In addition, **ODN5**, which has a nitrobenzyl protecting group with alkyl ether structures introduced into the benzene ring, showed a higher separation performance than **ODN2** and **ODN3**, with a retention time similar to that of **ODN1** (**ODN5**: 11.5 min). In addition, **ODN6**, which has a hydrophobic protecting group with a *tert*-butyl group introduced at the benzyl position, exhibited a longer retention time, as expected (**ODN6**:11.9 min). These results indicate that modifications in the structure of the hydrophobic tags can modulate the separation performance of RP-HPLC. The stability of the tag moiety in the deprotection reaction is critical for the hydrophobic protective groups. The analysis of the crude DNA mixture revealed that a peak of 5′-monophosphate DNA at a retention time of 7.08 min was produced, which indicate the tag was removed in the basic condition. **ODN5** and **ODN6**, despite having relatively long retention times and high separation performance, produced a 5′-monophosphate species by hydrolysis of the hydrophobic protecting group at approximately 7.08 min, resulting in a yield of the desired hydrophobically tagged ODN of less than 37%. For **ODN2** (with an *o*-nitrobenzene group at the benzyl position), **ODN3** (with a *tert*-butyl group), and **ODN4** (with a C19 alkyl group), the 5′-monophosphate DNA was produced in 15.6%, 8.3% and 11.8% yield, respectively. This suggests that the introduction of a *tert*-butyl group at the benzyl position improves the hydrolytic resistance of hydrophobic protecting groups. Therefore, our newly developed phosphoramidite reagent **3** can be considered as the optimal structure, which shows superior performance in both stability under strongly basic conditions and separation ability on RP-HPLC to give the desired ODN in 92% yield (Table 2).

**Table 1.** Synthesized 5′-phosphate DNAs with various phosphate-protecting groups

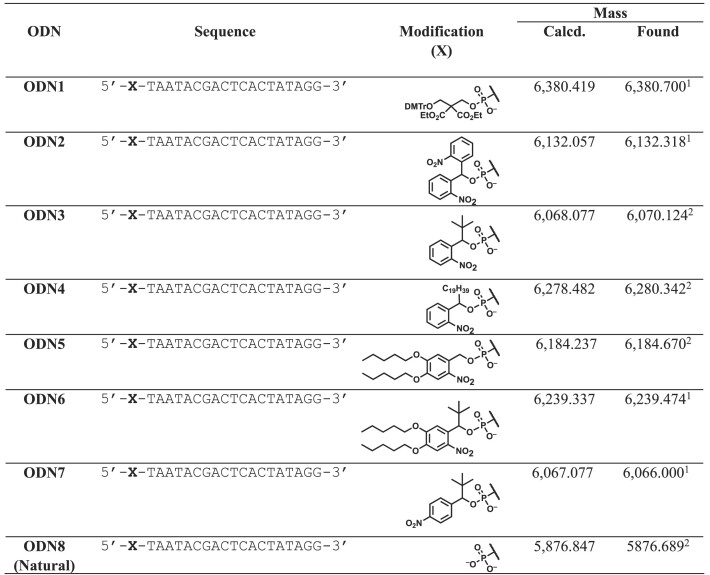

^1^The compounds were analyzed by LC–MS, and the multivalent mass spectra were deconvoluted to obtain mass data.

^2^The compounds were analyzed using MALDI-TOF-MS to obtain mass data. The analyses confirmed the presence of the target peaks; however, the primary peaks observed were those from which the protective groups were removed by laser irradiation during the measurement in the MALDI-TOF MS analysis.

**Table 2.** HPLC retention times, HPLC yields of **ODN1–8** and deprotection yields under reductive condition/UV irradiation

				Deprotection (%)		
Compound	Retention time (min)	ODN1–7 yield (%)^1^	ODN8 yield (%)^1,2^	Reductive condition^3^ (%, 37°C, 30 min)	Reductive Condition^3^ (%, best condition)	UV-Irradiation^8^ (%)
**ODN1**	11.5	76.4	9.46	–	–	–
**ODN2**	8.66	76	15.6	–	–	>99
**ODN3**	8.87	91.7	8.27	2	46 ^4^	>99
**ODN4**	15.1	82.9	11.8	42	98 ^5^	>99
**ODN5**	11.5	36.6	40.2	85	97 ^6^	No data
**ODN6**	11.9	31.4	66.4	No data	No data	No data
**ODN7**	9.27	59.1	24.3	–	>99 ^7^	–
**ODN8 (Natural)**	7.08	–	–	–	–	–

^1^calculated from HPLC peak area

^2^Produced by 28% NH_4_OH treatment for the synthesis of **ODN1–7**, ^3^Reduction condition: Na_2_S_2_O_4_ (1000 eq.), in Tris HCl buffer (pH 8.5), ^4^37°C, 30 min + 95°C, 20 min, ^5^37°C, 30 min + 95°C, 20 min, ^6^37°C, 30 min + 65°C, 10 min, ^7^37°C, 30 min + 50°C, 120 min, ^8^Photo-irradiation condition: 365 nm, 10 min, 4000 μW/cm^2^

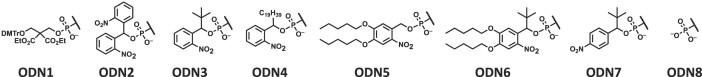

We also synthesized **ODN7** using a *p*-nitrobenzyl derivative (**7**) as a protective group that could be removed under reductive conditions. The HPLC yield of **ODN7** was 59.1%, which was 32.6% lower than that of **ODN3**. **ODN7** tended to have a slightly longer retention time (9.27 min) on RP-HPLC compared to that of **ODN3**, indicating the *p*-nitrobenzyl group shows higher hydrophobicity than the *o*-nitrobenzyl group.

### Investigation of deprotection conditions of nitrobenzyl groups

We investigated the reaction conditions for removing the hydrophobic tag from phosphorylated DNA isolated and purified by RP-HPLC. In particular, we used UV irradiation of *the o*-nitrobenzyl derivatives for the deprotection reaction (25). Specifically, **ODN3** and **ODN4** were dissolved in 20 mM Tris–HCl buffer (pH 8.5) and irradiated with 365 nm light at an intensity of 4 mW/cm^2^ for 10 min. The progress of the deprotection reaction was monitored by analyzing the samples before and after irradiation using RP-HPLC (Supplementary Figure S15). The results showed a high deprotection rate of more than 99% for all ODNs (Table 2). The proposed mechanism of the deprotection reaction by UV irradiation is shown in Scheme S8A (32) (33).

Furthermore, deprotection of **ODN3**, **ODN4** and **ODN5** was examined using a reduction reaction with sodium dithionite (Supplementary Figures S10–S13) (29). The proposed mechanism of the deprotection reaction by the reduction is shown in Scheme S8B (34). The experimental results showed that the nitro groups were reduced to amino groups when sodium dithionite was added and the mixture was heated at 37°C for 30 min. However, further heating was required to completely remove the protecting group; heating at 95°C for 20 min resulted in 46% of **ODN3** being converted to deprotected DNA, and 54% (converted) to a by-product with SO_2_ added to the amino group (Supplementary Figures S10 and S11, Supplementary Table S1). For **ODN4**, heating at 95°C for 20 min resulted in a high deprotection efficiency of 98% (Supplementary Figure S12). This indicates that the use of compound **4** is more suitable for reductive deprotection than compound **3**. Furthermore, **ODN5**, with a protecting group having a pentoxyether structure, was quantitatively deprotected after incubation at 65°C for 10 min (Supplementary Figure S13). However, the deprotection of *o*-nitrobenzyl derivatives requires heating and is not suitable for the purification of chemically unstable oligonucleotides such as long-stranded RNA. We also examined deprotection under reductive conditions using *p*-nitrobenzyl derivatives and found that **ODN7** was almost quantitatively deprotected after treatment at 37°C for 30 min followed by 50°C for 2 h (Supplementary Figure S14). From these results, we concluded that milder conditions under UV irradiation were optimal for the deprotection of 5′-monophosphorylated RNA, and in subsequent experiments, we mainly used phosphoramidite reagent **3**.

### Complete chemical synthesis of mRNA from 5′-monophosphate RNA and biological evaluation

**Figure 5.**
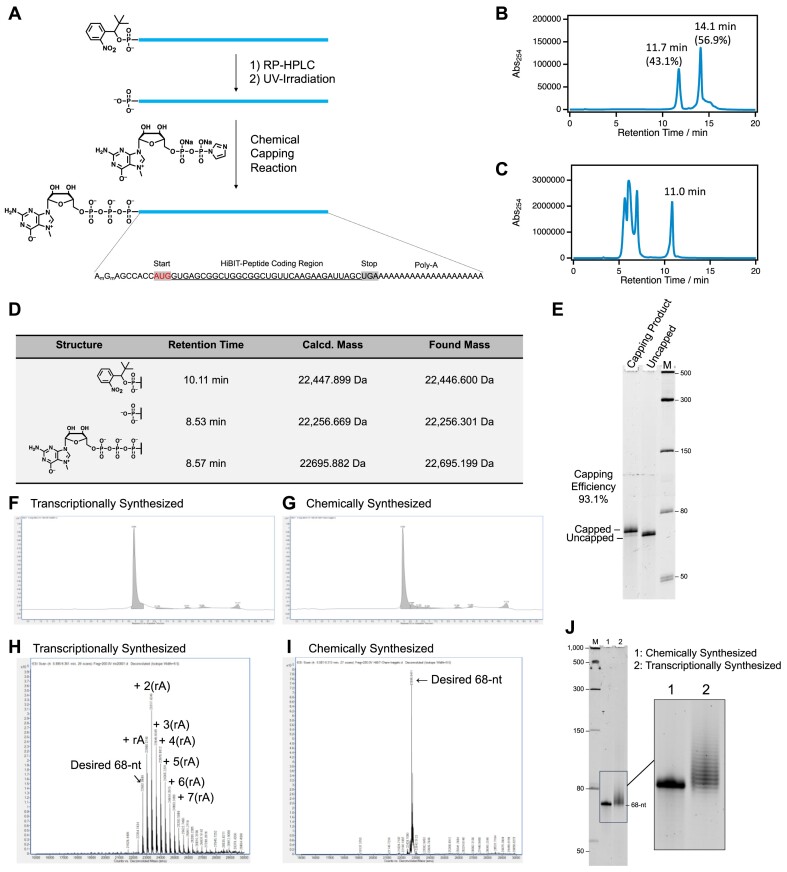
Chemical synthesis of messenger RNA by chemical capping reaction of 5′-monophosphate RNA purified by using nitrobenzyl hydrophobic tag: (**A**) Design and synthetic scheme of HiBiT-mRNA, (**B**) RP-HPLC profile of crude product containing 5′-Nb-protected RNA (RT = 14.1 min) and undesired shorter RNA (RT = 11.7 min) that failed to be elongated on the solid-phase chemical synthesis (Column: Hydrosphere C18 (250 × 4.6 mmI.D., S-5 μm, 12 nm), Solvent A: 50 mM TEAA buffer (pH 7.0) + 5% CH_3_CN, Solvent B: CH_3_CN, Gradient: 5–40%B over 20 min, column temperature 50°C, Flow rate: 1 ml/min, detection: 254 nm, loop size: 2.0 ml), (**C**) RP-HPLC profile of crude product of chemical capping reaction containing desired 5′-Capped minimal mRNA (RT = 11.0 min) and residue of chemical capping reagent (RT = 5.0–7.5 min) (Column: Hydrosphere C18 (250 × 4.6 mm I.D., S-5 μm, 12 nm), Solvent A: 50 mM TEAA buffer (pH 7.0) + 5% CH_3_CN, Solvent B: CH_3_CN, Gradient: 5–40% B over 20 min, column temperature 50°C, Flow rate: 1 ml/min, detection: 254 nm, loop size: 2.0 ml), (**D**) Result of LC-MS characterization of synthesized Nb-protected RNA, 5′-phosphate RNA, and Capped-mRNA, (**E**) Result of 15% dPAGE analysis to check purities of RNA before and after chemical capping reaction (1 x TBE buffer, gel size: 20 × 20 cm, 30 W, 2 h, SYBR Green II Stain), (**F, G**) UPLC profiles of transcriptionally and chemically synthesized HiBiT-mRNA (UPLC System: Agilent 1290 Infinity II -6530 LC/Q-TOF system, Column: ACQUITY UPLC® Oligonucleotide BEH C18 Column, 130Å, 1.7 μm, 2.1 mm x 50 mm, 1/pkg. part No. 18003949, ser No. 04133201918322, Column temp.: 60 degC, Detection: 260 nm, Flow rate: 0.3 ml/min, Solvent A: 100 mM HFIP buffer + 8.6 mM TEA (pH8.3), Solvent B: MeOH, Gradient Program: 0–30%B (0–12.0 min), 30–90% B (12.0– 12.1 min), 90%B (12.1– 15.0 min), 90–0% B (15.0– 15.1 min), 0%B (15.1–20.0 min)), (**H**, **I**) Deconvoluted mass spectra of transcriptionally and chemically synthesized HiBiT-mRNAs which were collected by using Agilent 1290 Infinity II – 6530 LC/Q-TOF system, **(J)** 10% dPAGE analysis of the chemically synthesized and transcriptionally synthesized HiBiT-mRNAs (1× TBE buffer, gel size; 8 × 8 cm, 30 mA, 2 h, SYBR Green II stain).

We carried out a complete chemical synthesis of the mRNA expressing the HiBiT peptide. First, we synthesized a 68-nucleotide 5′-phosphorylated RNA using phosphorylation reagent **3**. Subsequently, we were able to chemically synthesize mRNA with a cap structure by applying a previously developed chemical capping reaction (Figure 5A) (3). The synthesis of 5′-phosphorylated RNA was performed using the phosphoramidite method, according to the general RNA solid-phase synthesis protocol. RNA was released and deprotected from the solid support by treatment with a 1:1 mixture of 28% aqueous ammonia and 40% methylamine at 65°C for 15 min. This was followed by the removal of the triisopropylsilyloxymethyl protecting group at the 2′-hydroxyl using a 1 M tetrabutylammonium/THF solution at room temperature for 16 h. The resulting product was analyzed using RP-HPLC, and two distinct peaks were observed (Figure 5B). The peak with a retention time of 11.7 min contained short RNAs that were incompletely synthesized on the solid-phase and unreacted RNA (43.1% from HPLC peak area), whereas the peak with a retention time of 14.1 min contained the target 5′-phosphorylated RNA with the nitrobenzyl hydrophobic protecting group (56.9% from HPLC peak area). By collecting the peak with a retention time of 14.1 min using RP-HPLC, we were able to isolate and purify the target 5′-phosphorylated RNA with the protecting group (Supplementary Figure S16 and Supplementary Figure S17). The protective group was removed by UV irradiation (365 nm for 10 min) (Supplementary Figure S18). The 5′-cap structure was chemically introduced into 5′-phosphorylated RNA by reacting it with a chemical capping reagent in the presence of 1-methylimidazole in DMSO. By collecting and purifying the peak around the 11 min retention time with RP-HPLC, we synthesized mRNA with the desired 5′-cap structure (Figure 5C). The obtained RNA was characterized by LC-MS analysis, which confirmed a shift in the retention time of the peaks in the LC profile (Figure 5D). The 5′-phosphorylated RNA and 5′-capped mRNA were analyzed by denaturing polyacrylamide gel electrophoresis (dPAGE) containing 7 M urea, which showed that the acquisition of RNA of the target length and the efficiency of the chemical capping reaction was 93.1% (Figure 5E). Using this method, we were able to produce high-purity 5′-phosphorylated RNA, realizing chemical synthesis of mRNA (Supplementary Figure S19 and Supplementary Table S2). We also synthesized 107- and 131-nucleotide 5′-phosphorylated RNA using a similar approach, confirming that isolation and purification by RP-HPLC was feasible (Supplementary Table S3 and Supplementary Figures S20–S22). These results suggest that this method is also applicable for the purification of long-chain RNA of more than 100 nucleotides. To compare the chemical synthesis of mRNA, we conducted the synthesis of HiBiT-mRNA through *in vitro* transcription. UPLC profiles indicated that both chemically and transcriptionally synthesized HiBiT-mRNA displayed almost single peaks, signifying good purity (Figure 5F and G). However, the mass spectrum of transcriptionally synthesized mRNA showed a diversity in poly-A tail length (Figure 5H). Conversely, the mass spectrum of chemically synthesized mRNA was almost singular, suggesting better purity (Figure 5I). Furthermore, dPAGE analysis of chemically and transcriptionally synthesized mRNA also supports the differences in purities (Figure 5J). These results demonstrated that the chemical synthesis method can produce mRNA with a homogeneous poly-A tail, which is challenging to achieve with the transcriptional synthesis method.

**Figure 6.**
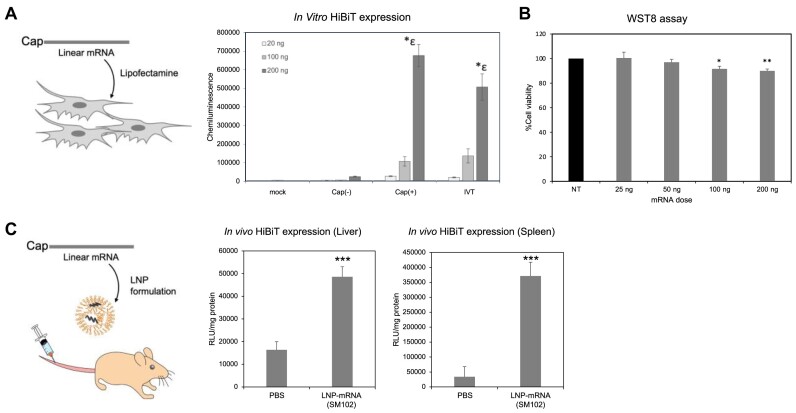
Biological activities of synthesized mRNAs: (**A**) *In vitro* HiBiT expression using HeLa cells: HeLa cells were seeded on 96-well plates at 10 000 cells/well. mRNA transfection was performed by using lipofectamine® messenger MAX. After incubation at 37°C for 8 h in a 5% CO_2_ atmosphere, a HiBiT lytic assay was performed (ϵ; ****P< 0.0001 versus cap−, cap+, 20 ng or mock, Δ; ****P< 0.0001 versus cap−, cap+, 20 ng or mock, ***P< 0.001 versus cap+, 100 ng). (**B**) Evaluation of cell viability: HeLa cells were seeded in 96-well plates at 6000 cells/well. mRNA transfection was performed by using lipofectamine® messenger MAX. After incubation at 37°C for 24 h in a 5% CO_2_ atmosphere, the WST8 assay was performed. (**C**) *In vivo* HiBiT expression assay in C57BL 6N-5w-female mouse (10 μg/mouse, *N* = 3). The LNP composition was SM102/DOPE/Cholesterol/DMG-PEG2k of 50/10/38.5/1.5 molar ratio (lipid/RNA = 400 nmol/10 μg). After 6 h, the liver and the spleen were collected and lysed. HiBiT expression was quantified using a HiBiT lytic assay system (***P < 0.001, versus PBS).

We performed biological experiments to evaluate the functionality of chemically synthesized HiBiT-mRNA. Both RNA without a 5′-cap structure and mRNA with a 5′-cap structure were transfected into HeLa cells. After 8 h incubation, the expression of HiBiT was quantified using the Nano-Glo^®^ HiBiT assay system (Figure 6A) (35). These results confirmed that the addition of a 5′-cap structure resulted in an approximately 10-fold increase in translational activity, and HiBiT expression was increased in a dose-dependent manner. Then, HiBiT-mRNA was introduced into HeLa cells by lipofection and incubated for 24 h. Cell viability was measured using the WST-8 assay (36), and virtually lower cytotoxicity (%Cell viability > 85) was observed at mRNA doses ranging from 25 to 200 ng (Figure 6B). *In vivo* expression of HiBiT peptide was also evaluated. HiBiT-mRNA was encapsulated in lipid nanoparticles (LNP) and intravenously injected into C57BL/6N mice (female, 5-week-old) at a dose of 10 μg of mRNA per mouse. The LNP was prepared based on the formulation used in clinical use (ionizable lipid/phospholipid/cholesterol/PEG-lipid = 50/10/38.5/1.5 molar ratio) (37) and SM102 was used as the ionizable lipid (38,39). At 6 h after the administration, the liver and the spleen were collected, and the luminescence of each tissue lysate was measured using a HiBiT Lytic assay. Significant HiBiT expressions in the liver and the spleen were confirmed (Figure 6C).

### Synthesis of circular mRNA from transcribed 5′-monophosphate RNA and biological evaluation

**Figure 7.**
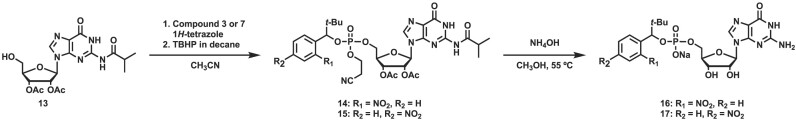
Synthesis of guanosine-5′-monophosphate with a Nitrobenzyl protecting group (**16**, **17**).

To synthesize long-chain 5′-monophosphorylated RNA *via* transcription reactions, we developed nitrobenzyl-modified guanosine 5′-monophosphate (RpG) as a substrate (Figure 7). The use of RpG allows it to compete with GTP during transcription reactions mediated by T7 RNA polymerase, producing a mixture of 5′-triphosphorylated RNA and nitrobenzyl-modified 5′-monophosphorylated RNA. The hydrophobic nature of the nitrobenzyl group is expected to allow selective isolation and purification of 5′-monophosphorylated RNA alone by RP-HPLC. Starting from 2′,3′-*O*-acetyl-*N*^2^-isobutyrylguanosine (**13**) (40), newly developed phosphorylation reagents **3** and **7** were reacted in the presence of 1*H*-tetrazole, and the resulting phosphite intermediates were oxidized with *tert*-butylhydroperoxide to give compounds **14** and **15** with a yield of 42.2% (Figure 7). Subsequently, the cyanoethyl group on the 5′-phosphate, isobutyryl group on the nucleobase, and acetyl groups on the hydroxyl groups of compounds **14** and **15** were removed in a 1:1 mixture of acetonitrile and 28% aqueous ammonia under heating conditions to synthesize the desired *o*-Nb-RpG (**16**) and *p*-Nb-RpG (**17**) with a yield of 72.3% (Figure 7). RNA synthesized with *o*-Nb-RpG (**16**) can have the *o*-nitrobenzyl group removed by UV irradiation. RNA synthesized with *p*-Nb-RpG (**17**) can have a *p*-nitrobenzyl group removed under reductive conditions.

**Figure 8.**
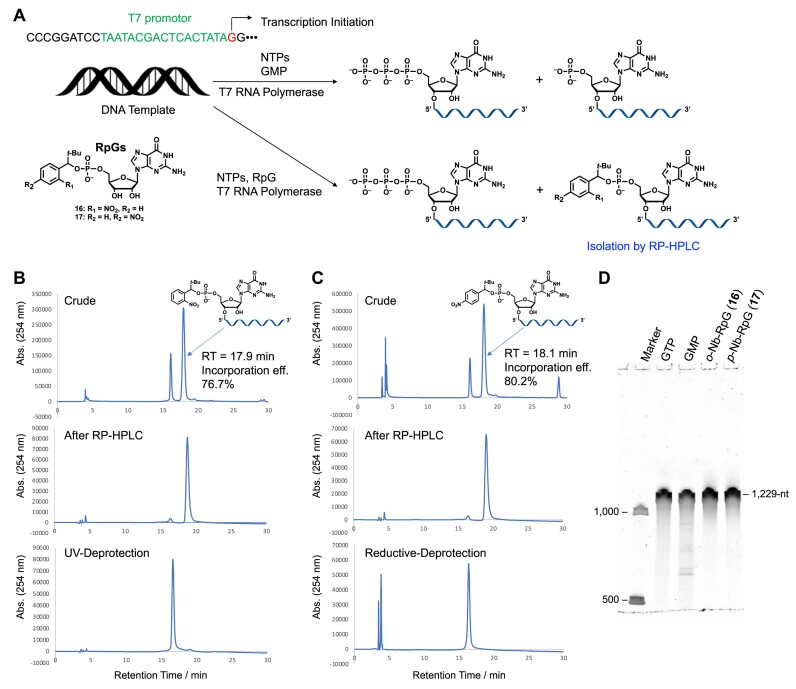
Synthesis of 5′-monophosphate RNA consisting of EMCV-IRES and NanoLuc-coding sequences by *In vitro* transcription: (**A**) Experimental design of *in vitro* transcription, (**B**) RP-HPLC profiles of synthesized RNA by *in vitro* transcription with RpG **16** before/after purification and after UV-deprotection (column: YMC Triart Bio C4 (250 × 4.6 mm I.D., S-5 μm, 30 nm), solvent A: 100 mM TEAA buffer (pH 7.0) + 5% CH_3_CN, solvent B: 50% CH_3_CN/100 mM TEAA buffer (pH 7.0), gradient: 15–30% B over 20 min, column temperature 50°C, flow rate: 1 ml/min, detection: 254 nm, loop size: 2.0 ml). The incorporation efficiency of RpG **16** was calculated to 76.7% from the peak ratio of 5′-*o*-Nb-RpG-RNA and 5′-triphosphate RNA, (**C**) RP-HPLC profiles of synthesized RNA by *in vitro* transcription with RpG **17** before/after purification and after reductive-deprotection (YMC Triart Bio C4: solvent A, 50 mM TEAA (pH 7.0), 5% ACN; solvent B, ACN; gradient 0–100% B/ 0–20 min; flow rate, 1 ml/min; column temperature, 50°C; column size 250 × 4.6 mm l.D., S-5 μm,. 12 nm; detection wavelength, 260 nm). The incorporation efficiency of RpG **17** was calculated to 80.2% from the peak ratio of 5′-*p*-Nb-RpG-RNA and 5′-triphosphate RNA, (**D**) result of 5% dPAGE analysis to check purities of RNA after RP-HPLC purification and deprotection (1 × TBE buffer, gel size: 8 cm × 8 cm, 26 mA, 60 min, SYBR Green II stain).

We performed *in vitro* transcription reactions to synthesize a 1229 nucleotide 5′-monophosphorylated RNA. The 1255-nucleotide template DNA used for transcription encoded encephalomyocarditis virus (EMCV)-derived IRES (41) and NanoLuc luciferase (NanoLuc). Using GMP as a substrate, the peaks of the 5′-triphosphate and 5′-monophosphate species overlapped, making separation difficult. However, by adding RpGs instead of GMP, we were able to clearly separate the RpG-containing RNA and the 5′-triphosphate species using RP-HPLC (Figure 8A, Supplementary Figures S24–S30, and Supplementary Table S4, Supplementary Table S5). When transcribed with the *o*-nitrobenzyl derivative (**16**), 5′-*o*-Nb-RpG RNA appeared with a retention time of 17.9 min on RP-HPLC and was completely separated from the 5′-triphosphate species (retention time 16.1 min). After isolation of the 5′-*o*-Nb-RpG-RNA peak and removal of the *o*-nitrobenzyl group by UV irradiation, a peak appeared with a retention time of 16.6 min, allowing high-purity recovery of 5′-monophosphate RNA (Figure 8B). In the case of transcription using the *p*-nitrobenzyl derivative (**17**), 5′-*p*-Nb-RpG-RNA appeared on RP-HPLC with a retention time of 18.1 min, and was also completely separated from the 5′-triphosphate species (retention time 16.1 min). After isolating the peak of 5′-*p*-Nb-RpG-RNA and completing deprotection by treating it at 50°C for 4 h, we were able to obtain high-purity 5′-monophosphate RNA (retention time 16.4 min, Figure 8C). As a result of the dPAGE analysis of the transcriptionally synthesized RNA, products of the desired length were obtained in both cases of *o*-Nb-RpG (**16**) and *p*-Nb-RpG (**17**), and the purity was higher than that of the transcript using GMP (Figure 8D). This method demonstrates the feasibility of purifying long-chain RNA of >1000 nucleotides, synthesized by *in vitro* transcription. Furthermore, in the transcription reaction using RpGs as substrates, we analyzed the separation ability of RNA products of different lengths (34, 100, 250, 650 and 1078 nucleotides) in RP-HPLC (Supplementary Table S6, Supplementary Figure S23). As the length increases, the difference in retention time between RpG-RNA and pppRNA tends to shorten, but they were still separable even at 1078 nucleotides (Supplementary Figures S24–S30).

**Figure 9.**
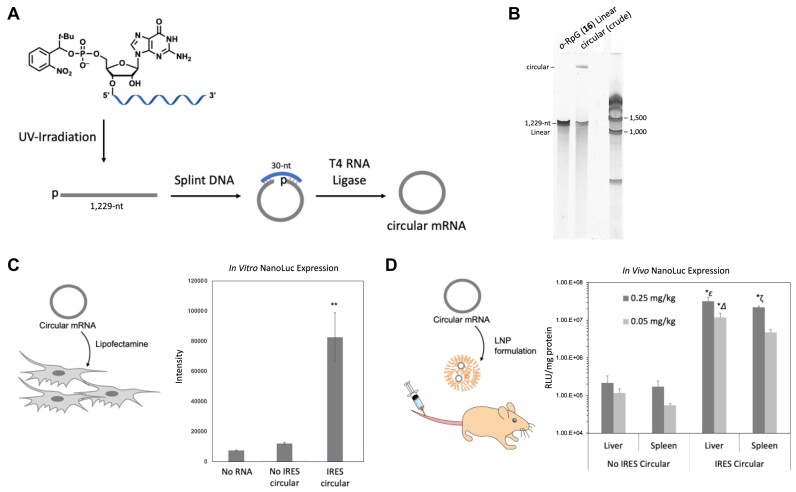
Synthesis and translational activities of circular mRNAs: (**A**) synthetic scheme of circular mRNA, (**B**) result of 5% dPAGE analysis: linear IRES-mRNA, *o*-RpG (**16**) without the hydrophobic tag was prepared by *in vitro* transcription and photo irradiation. The crude IRES circular mRNA was prepared from Linear IRES-mRNA, *o*-RpG (**16**) by ligation reaction using T4RNA ligase and splint DNA. Gel analysis condition: 1× TBE buffer, gel size: 8 cm × 8 cm, 26 mA, 120 min, SYBR Green II stain. The circularization efficiency was calculated to 53.0% from the band intensity ratio of linear and circular mRNAs. Gel purification was performed to obtain circular mRNA and used for translation experiments. (**C**) *In vitro* NanoLuc expression levels using HeLa Cells (seeding 1.0 × 10^4^ /100 μl 96-well plate, Lipofectamine^®^ Messenger MAX Reagent, 10 ng mRNA, 0.15 μl Lipofectamine/ 5 μl Opti-MEM. After 24 h incubation at 37°C, Nano-Glo^®^ luciferase assay (Promega N1110) was performed (ϵ; ***<0.005 versus no IRES circular, no RNA), (**D**) *In vivo* NanoLuc expression assay (ICR-4w-female mice, 0.25 or 0.05 mg/kg, *N* = 3). The LNP composition was SM102/DOPE/cholesterol/DMG-PEG2k of 50/10/38.5/1.5 molar ratio (lipid/RNA = 400 nmol/10 μg). 24 h after LNP injection, the liver and the spleen were collected and lysed. Nano-Glo^®^ luciferase assay (Promega N1110) was performed to quantify the expression level of NanoLuc (ϵ; *P< 0.05 versus IRES spleen 0.25 mg/kg, ****P< 0.0001 versus IRES liver 0.05 mg/kg, IRES spleen 0.05 mg/kg, or no IRES, ; *P< 0.05 versus IRES Spleen 0.25 mg/kg, no IRES Liver 0.05 or 0.25 mg/kg, spleen 0.25 mg/kg, **P< 0.01 versus No IRES spleen 0.05 mg/kg, ζ; ***P< 0.001 versus IRES spleen 0.05 mg/kg, ****P< 0.0001 versus no IRES).

Circular mRNA synthesis was performed using highly purified 5′-monophosphorylated RNA synthesized by transcription as the starting material. The 5′-phosphorylated RNA of 1229 bases shown in Figure 8D was cyclized using T4 RNA ligase 2 in the presence of splint DNA (Figure 9A and Supplementary Figure S31). The results of dPAGE before and after cyclization are shown in Figure 9B. It was confirmed that the desired circular mRNA was produced with 53% cyclization efficiency. Similarly, circular mRNA without IRES was also synthesized (Supplementary Figure S32).

The synthesized circular mRNA and linear mRNA prepared from CleanCap were introduced into HeLa cells by lipofection and cultured for 24 h. NanoLuc expression was quantified using the Nano-Glo Luciferase Assay System (Figure 9C and Supplementary Figure S33). The results showed that translational activity was significantly increased in circular mRNA containing IRES, and the expression level was 6.9-fold higher than that of circular mRNA without IRES. *In vivo* expression of NanoLuc was also evaluated. Circular mRNAs encapsulated in LNP were intravenously injected into ICR mice (female, 4-week-old) at 0.25 or 0.05 mg/kg (5 or 1 μg per mouse, 20 g of mouse weight). The same LNP formulation as in the HiBiT experiment was used. At 24 h after administration, the liver and the spleen were collected. Each tissue sample was homogenized in lysis buffer, centrifuged, and the supernatant was quantified for NanoLuc expression using the Nano-Glo^®^ Luciferase Assay System (Figure 9D). The results confirmed NanoLuc expression in both tissues, and circular mRNAs containing IRES showed more than 100-times higher translational activity than those without IRES. These results suggested that IRES recruits ribosomes and enhances NanoLuc expression.

## Conclusion

Novel phosphorylation reagents capable of synthesizing high-purity 5′-phosphorylated DNA and RNA were developed in this study. We demonstrated that these phosphorylation reagents allowed the isolation and purification of chemically synthesized phosphorylated DNA/RNA by RP-HPLC. Further detailed investigations of the deprotection conditions of the developed hydrophobic tags revealed that deprotection is feasible under UV irradiation and reductive conditions. Using chemically synthesized 5′-phosphorylated RNA in chemical capping reactions, we demonstrated the complete chemical synthesis of mRNA. RpG analogs have also been developed as substrates for *in vitro* transcription reactions to synthesize 5′-monophosphorylated RNA consisting of over 1000 bases with a hydrophobic tag at the 5′-end, which could be isolated by RP-HPLC. In addition, we used high-purity 5′-monophosphorylated RNA synthesized using the RpG method for the synthesis of circular mRNA by cyclization using T4 RNA ligase. This technology is expected to be applied to the efficient and high-purity synthesis of functional mRNA, transfer RNA, and other biologically important RNAs, paving the way for advances in RNA-based therapeutics and research applications.

## Supplementary Material

gkae847_Supplemental_File

## Data Availability

The data underlying this article will be shared upon reasonable request by the corresponding author.
