# Supplementary material for: Development of hydrophobic tag purifying monophosphorylated RNA for chemical synthesis of capped mRNA and enzymatic synthesis of circular mRNA
Source: Nucleic Acids Res. 2024 Oct 17;52(20):12141–57. doi: 10.1093/nar/gkae847 (PMC11551738; doi:10.1093/nar/gkae847)
Supplement: gkae847_Supplemental_File [file gkae847_supplemental_file.pdf]

Supporting Information

Table of Contents

1. **General Information and Instrument**

2. **Experimental Procedures**
    - 2-1. Synthesis of Phosphoramidites
    - 2-2. Synthesis of RpGs
    - 2-3. Oligo Synthesis, Purification, and Deprotection
    - 2-4. Chemical Synthesis of minimal mRNA
    - 2-5. Biological activity evaluation of chemically synthesized minimal mRNA
    - 2-6. *In Vitro* Transcribed Synthesis of RNA by Using RpGs
    - 2-7. Synthesis and Evaluation of Circular mRNA

3. **Compounds Spectral Data**

4. **HPLC Profiles, MALDI-TOF-MS Spectra, dPAGE Analysis of Synthesized DNAs and RNAs**

5. **Investigation of Deprotection Conditions of Nitrobenzyl Groups**

6. **Spectral Data of Chemically Synthesized RNAs**

7. **Preparation of IVT-mRNA Which Codes HiBiT Peptide**

8. **Analysis of 5'-phosphate RNAs Which Synthesized by *In Vitro* Transcription**

9. **dPAGE Analysis for Synthesis of Circular mRNA**

10. **Comparison of *In Vitro* Translational Activity of Circular and Linear mRNAs**

11. **DNA/RNA Sequences for In Vitro RNA Synthesis and Circular mRNA Synthesis**

12. **References**

1. General Information and Instrument

Standard abbreviations for the protecting groups are followed by the IUPAC-IUB Commission on Biochemical Nomenclature. All starting materials, reagents, and solvents of guaranteed grade, were purchased from FUJIFILM Wako Chemicals, Tokyo Chemicals, Sigma-Aldrich, or Kanto Chemicals and used without further purification. All reactions involving moisture sensitive reagents were performed under an argon atmosphere using oven dried glassware. Column chromatography was performed on silica gel (63–210 mesh) purchased from Kanto Chemicals. All solid-phase oligonucleotide synthesis reagents were purchased from Chem Genes or Glen Research. All solvent compositions are reported in volume % unless specified otherwise. Syntheses of oligonucleotides were performed on a DNA/RNA synthesizer NR-2A_7MX or NRs-4A_10R7NP (Nihon Techno Service). NMR spectra were taken on JOEL NMR-ECS 400 (400 MHz for ^{1}H NMR, 101 MHz for ^{13}C NMR, and 163 MHz for ^{31}P NMR), and JOEL NMR-ECS 600 (600 MHz for ^{1}H NMR, 151 MHz for ^{13}C NMR, and 243 MHz for ^{31}P NMR) instruments. The ^{1}H and ^{13}C{^{1}H} NMR chemical shifts (δ) are reported in parts per million (ppm) relative to residual solvents: $CHCl_3$ (7.26 ppm for ^{1}H NMR, 77.16 ppm for ^{13}C NMR), CD_3OD (3.31 ppm for ^{1}H NMR, 49.00 ppm for ^{13}C NMR), DMSO-d_6 (2.50 ppm for ^{1}H NMR, 39.52 ppm for ^{13}C NMR), and D_2O (4.79 ppm for ^{1}H NMR). The purity of the DNA and RNA was confirmed by denaturing polyacrylamide gel electrophoresis (dPAGE) containing 7.5 M urea as a denaturant (acrylamide:*N*,*N*'-methylenebis(acrylamide) = 19:1, electrophoresis buffer: 1x TBE, band visualization by SYBR Green II). ESI-TOF mass spectra were obtained on a micro TOF-QII (Bruker Daltonics) instrument. MALDI-TOF mass spectra were obtained on an UltrafleXtreme (Bruker Daltonics) with 3-hydroxypicolinic acid as a matrix to detect the peaks of the synthesized RNAs. LCMS analyses of the synthesized oligonucleotides were performed by using Agilent 1290 Infinity II - 6530 LC/Q-TOF system and Waters ACQUITY H-Class PLUS_LBNW - Xevo G2-XS Qtof System_NQTW.

2. Experimental Procedures

2-1. Synthesis of Phosphoramidites

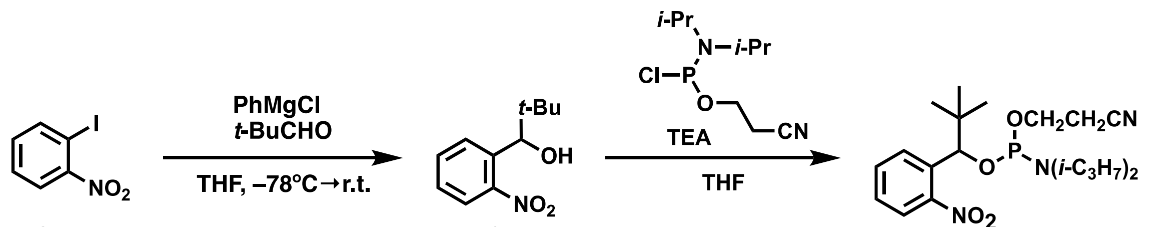

8 9 3

Scheme S1. Synthesis of compound **3**.

***t*-Bu-nitrobenzyl alcohol (9):** 1-iodo-2-nitrobenzene (**8**) (5.0 g, 20 mmol) was dissolved in THF (50 mL) and cooled at -78 °C. PhMgCl (11 mL, 22 mmol) was added dropwise and stirred for 30 minutes then, pivalaldehyde (2.2 mL, 20 mmol) was added dropwise and stirred for 3 hours. After stirring the mixture, NH_4Cl aq. (100 mL) was added for quench and extract with EtOAc (200 mL x3). The solution was washed with water (100 mL) and brine (100 mL), and organic layer was dried over Na_2SO_4. The filtrate was evaporated *in vacuo* and purified by column chromatography on silica gel (hexane/ethyl acetate = 20/1 to hexane/ethyl acetate = 4/1) to get desired compound **9** (134 g, 62 mmol, 65%) as brown solid. ^{1}H-NMR (400 MHz, $CDCl_3$) δ 7.80

(dd, J = 7.9, 1.4 Hz, 1H, Ph-CH), 7.74 (dd, J = 8.0, 1.2 Hz, 1H, Ph-CH), 7.61-7.57 (m, 1H, Ph-CH), 7.42-7.37 (m, 1H, Ph-CH), 5.37 (d, J = 3.6 Hz, 1H, CH of benzyl), 2.07 (d, J = 3.9 Hz, 1H, OH), 0.88 (s, 9H, CH_3 of *t*-Bu) ppm. HRMS (ESI) *m/z* calculated for, $[M+H]^+$: 210.113, found for $[M+H]^+$: 210.1094. All the spectral data are consistent with the literature reports (1,2).

***t*-Bu-nitrobenzyl phosphoramidite (3):** Compound **9** (1.0 g, 4.8 mmol) and triethylamine (5.3 mL, 38 mmol, 8.0 eq.) was dissolved in dry THF (19 mL). Cyanoethyl-*N*,*N*′-diisopropylchlorophosphoramidite (4.3 mL, 19 mmol) was added to the solution, and stirred at room temperature for 4 hours. After starting material disappeared on TLC, stopped the reaction and added 50 mL saturated $NaHCO_3$ *aq.* to the mixture. The mixture was extracted with EtOAc and washed 2-times with brine. The organic layer was dried over Na_2SO_4 and concentrated. The residue was purified by silica gel column chromatography on neutral silica gel column using the eluent gradient of (hexane/EtOAc = 3/1 + 1% triethylamine) to afford the target compound. Fractions containing the target product were collected, evaporated, and dehydrated under vacuum, afford the compound **3** (1.6 g, 83% yield). ^{1}H-NMR (400 MHz, $CDCl_3$) δ 7.70-7.77 (m, 2H, Ph-CH), 7.51-7.61 (m, 1H, Ph-CH), 7.36-7.41 (m, 1H, Ph-CH), 5.40-5.45 (m, 1H, CH of benzyl), 3.49-3.99 (m, 4H, -O$\underline{CH_2}\underline{CH_2}$CN), 2.62-2.76 (m, 1H, CH of *i*-Pr), 2.17-2.36 (m, 1H, CH of *i*-Pr), 1.27 (d, J = 7.0 Hz, 2H, CH_3 of *i*-Pr), 1.16 (dd, J = 10.3, 6.7 Hz, 6H, CH_3 of *i*-Pr), 0.99 (d, J = 6.7 Hz, 4H, CH_3 of *i*-Pr), 0.87 (d, J = 3.8 Hz, 9H, CH_3 of *t*-Bu); ^{13}C-NMR (101 MHz, $CDCl_3$) δ 149.78, 149.44, 135.62, 135.51, 131.78, 131.68, 131.07, 130.86, 128.09, 127.87, 123.86, 123.59, 117.75, 77.36, 77.21, 75.83, 75.71, 58.99, 58.76, 58.14, 57.91, 43.57, 43.42, 43.29, 37.23, 37.18, 37.12, 37.05, 25.89, 25.77, 24.83, 24.78, 24.73, 24.70, 24.67, 24.34, 24.27, 20.59, 20.53, 20.17, 20.10; ^{31}P-NMR (162 MHz, $CDCl_3$) δ 152.95, 148.78; HR-ESI-MS (m/z) calcd. for $C_{20}H_{32}N_3NaO_4P$ $[M+Na]^+$ 432.2028, found for 432.1818.

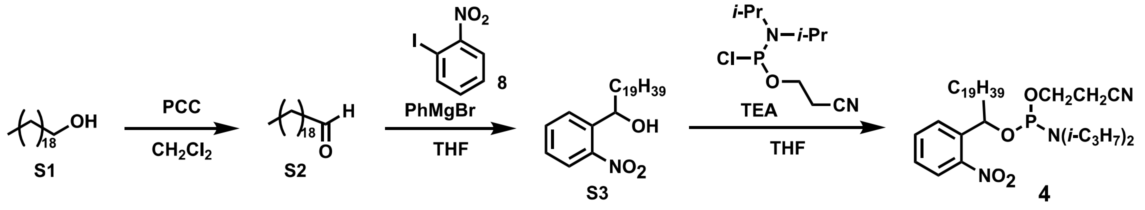

Scheme S2. Synthesis of Compound **4**.

C19-aldehyde (S2): 1-Eicosanol (**S1**) (3.00 g, 10.0 mmol) and Celite (4.00 g) were suspended in dehydrated dichloromethane (20.0 mL) at 0 °C. Pyridinium chlorochromate (3.23 g, 15.0 mmol) was added and the mixture was stirred at room temperature for 2 hours. After the reaction, the filtrate was collected by Celite filtration and concentrated. Purification by column chromatography (neutral flash silica, dichloromethane) afforded 2.80 g (9.50 mmol, 95% yield) of compound **S2** as a white solid. ^{1}H-NMR (400 MHz, $CDCl_3$) δ 9.76 (t, J = 1.6 Hz, 1H, C$\underline{H}$O of aldehyde), 1.29-1.25 (m, 36H, CH_2 x18), 0.88 (t, J = 7.2 Hz, 3H, CH_3) ppm. All the spectral data are consistent with the literature report (3).

C19-Nitrobenzyl alcohol (S3): 1-iodo-2-nitrobenzene (**8**) (2.65 g, 10.7 mmol) was dissolved in THF (25 mL). At -48 °C, phenylmagnesium chloride (2.0 M THF solution, 5.86 mL, 11.7 mmol) was added dropwise and the reaction was stirred at -35 °C for 10 min. A solution of compound **S2** (3.00 g, 10.1 mmol, 0.95 eq.) in THF (15 mL) was added dropwise to reaction mixture. The

reaction temperature was gradually raised to room temperature and stirred at room temperature for 15 min. Saturated aqueous ammonium chloride solution (10 mL) was added, and the mixture was extracted 2-times with ethyl acetate (100 mL), and the organic layer was washed with saturated brine (50 mL). The combined organic layer was concentrated. Purification by column chromatography (neutral flash silica, ethyl acetate/hexane = 1/15 → 1/10) afforded 3.44 g (8.65 mmol, 81% yield) of compound **S3** as a brown solid. ^{1}H-NMR (400 MHz, $CDCl_3$) *δ* 7.89 (dd, *J* = 8.2 Hz, 1.6 Hz, 1H, Ph-CH), 7.80 (dd, *J* = 6.4 Hz, 1.6 Hz, 1H, Ph-CH), 7.63 (td, *J* = 6.4 Hz, 1.2 Hz, 1H, Ph-CH), 7.41 (td, *J* = 6.0 Hz, 1.6 Hz, 1H, Ph-CH), 5.23 (q, *J* = 4.0 Hz, 1H, CH of benzyl), 2.09-1.78 (m, 1H, OH), 1.31-1.21 (m, 36H, $C\underline{H}_2$ x18), 0.88 (t, *J* = 7.2 Hz, 3H, CH_3) ppm. ^{13}C NMR (101 MHz, $CDCl_3$) *δ* 148.1, 140.3, 133.5, 128.1, 128.0, 124.4, 69.5, 38.3, 32.0, 29.8-29.4, 26.2, 22.8, 14.2 ppm. HR-ESI-MS calcd. for $C_{26}H_{45}NNaO_3^+$ (M + Na) $^+$ 442.3297, found 442.3421.

C19-nitrobenzyl phosphoramidite (4): C19-nitrobenzyl alcohol **S3** (100 mg, 0.24 mmol) was dissolved to anhydrous THF (1.0 mL) and triethylamine (2.5 mL, 1.9 mmol) was added to the mixture. 2-cyanoethyl diisopropylchlorophosphoramidite (0.21 mL, 4.5 g, 0.96 mmol) was added to the solution, and the mixture was stirred at room temperature for 4 hours. The reaction mixture was quenched by the addition of saturated $NaHCO_3$ *aq.* and extracted 3-times with EtOAc. The organic layer was washed with water and brine, and dried over Na_2SO_4. The filtrate was concentrated *in vacuo* and purified by column chromatography on silica gel (hexane/EtOAc=3/1 + 1% triethylamine). Fractions containing the target product were collected, evaporated, and dried under vacuum to afford compound **4** (162 mg, quant.) as yellow oil. ^{1}H-NMR (400 MHz, $CDCl_3$) δ 7.86 (dd, J = 8.3, 1.2 Hz, 1H, Ph-CH), 7.79 (qd, J = 8.0, 1.4 Hz, 1H, Ph-CH), 7.64-7.57 (m, 1H, Ph-CH), 7.40-7.35 (m, 1H, Ph-CH), 5.41-5.31 (m, 1H, CH of benzyl), 3.93-3.39 (m, 4H, -$OC\underline{H}_2C\underline{H}_2CN$), 2.71-2.58 (m, 1H, CH of *i*-Pr), 2.44-2.29 (m, 1H, CH of *i*-Pr), 1.84-1.68 (m, 2H, 2/3 CH_3 of *i*-Pr), 1.48-0.83 (m, 49H, CH_3 of *i*-Pr, -$C\underline{H}_2$- x18, and -$CH_2C\underline{H}_3$) ; ^{13}C-NMR (101 MHz, $CDCl_3$) *δ* 147.5, 140.0, 139.7, 133.2, 129.5, 129.3, 129.2, 127.9, 127.8, 124.1, 124.0, 117.7, 117.5, 71.8, 71.6, 70.6, 70.4, 58.9, 58.7, 58.2, 58.0, 43.4, 43.2, 43.1, 39.3, 39.1, 39.0, 32.0, 29.8, 29.7, 29.6, 29.5, 29.3, 25.9, 25.8, 24.8, 24.7, 24.6, 24.1, 24.1, 22.8, 20.5, 20.4, 20.2, 20.1, 14.2 ppm. ^{31}P-NMR (162 MHz, $CDCl_3$) δ 150.5, 148.8 ppm. HR-ESI-MS (m/z) calcd. for $C_{35}H_{62}KN_3O_4P$ [M+K]$^+$ 658.4115, found for 658.4246.

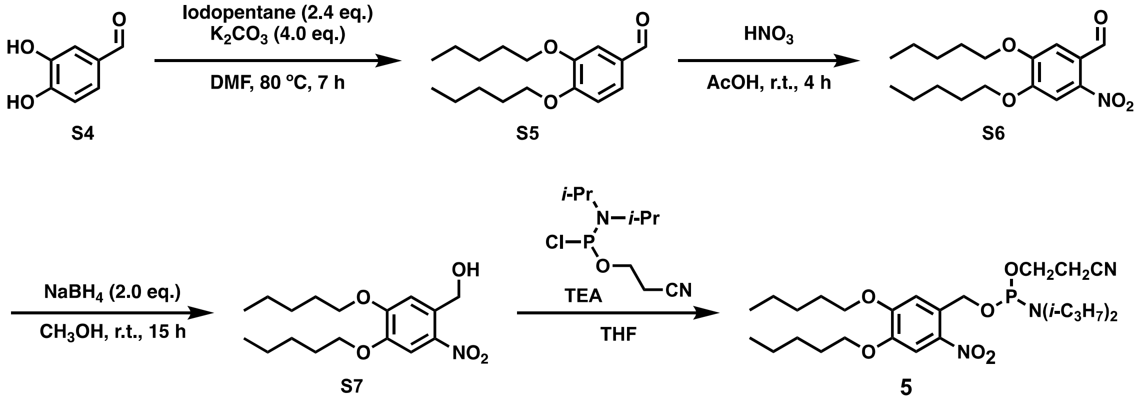

Scheme S3. Synthesis of Compound **5**.

3,4-Bis(pentyloxy)benzaldehyde (S5): To a mixture of 3,4-dihydroxybenzaldehyde (**S4**) (3.0

g, 22 mmol) and K_2CO_3 (12 g, 88 mmol) in dry DMF (50 mL) was added iodopentane (6.9 mL, 11 g, 53 mmol) and heated to 80 °C. After being stirred for 7 hours at 80 °C, the reaction mixture was cooled to room temperature. DMF was removed under reduced pressure, and the residue was dissolved in ethyl acetate and washed water. The organic layer was dried over Na_2SO_4 and concentrated. The crude material was purified by silica gel column chromatography eluted by 13→56% ethyl acetate/hexane, to afford compound **S5** (6.2 g, quant.) as brown solid. ^{1}H NMR (600 MHz, $CDCl_3$): δ 9.80 (s, 1H, CHO of aldehyde), 7.39–7.36 (m, 2H, Ph-CH x2), 6.92 (d, J = 8.4 Hz, 1H, Ph-CH), 4.09–4.01 (m, 4H, -OCH_2- x2), 1.86–1.79 (m, 4H, -CH_2- x2), 1.46–1.35 (m, 8H, -CH_2- x4), 0.92–0.89 (m, 6H, -CH_2CH_3 x2) ppm. ^{13}C NMR (151 MHz, $CDCl_3$): δ 191.05, 154.73, 149.49, 129.93, 126.69, 111.78, 110.94, 69.16, 60.44, 28.83, 28.74, 28.24, 28.20, 22.51, 22.49, 14.25, 14.09 ppm. ESI-TOF-MS calcd. for $C_{17}H_{26}NaO_3$, 301.1774 $[M + Na]^+$; found, 301.2054.

2-Nitro-4,5-bis(pentyloxy)benzaldehyde (S6): To a solution of compound **S5** (4.3 g, 16 mmol) in acetic acid (15 mL) was added dropwise HNO_3 (15 mL, ~70% purity) over 17 min. After being stirred for 4 hours at room temperature, the reaction mixture was quenched by the addition of ice. The resulting precipitate was collected by filtration and rinsed with water. The obtained solid was suspended in ethanol, and the resulting precipitate was collected by filtration and rinsed with ethanol to afford compound **S6** (3.1 g, 62% yield) as yellow powder. ^{1}H NMR (600 MHz, $CDCl_3$): δ 10.41 (s, 1H, CHO of aldehyde), 7.56 (s, 1H, Ph-CH), 7.36 (s, 1H, Ph-CH), 4.13–4.08 (m, 4H, -OCH_2- x2), 1.90–1.83 (m, 5H, -CH_2-), 1.49–1.36 (m, 11H, -CH_2-), 0.94–0.91 (m, 8H, -CH_2- x2 and -CH_2CH_3 x2) ppm. ^{13}C NMR (151 MHz, $CDCl_3$): δ 188.01, 153.27, 152.37, 151.85, 143.67, 125.34, 110.69, 108.20, 107.94, 70.26, 69.92, 69.78, 28.55, 28.52, 28.46, 28.11, 28.06, 22.42, 22.38, 14.05 ppm. ESI-TOF-MS calcd. for $C_{17}H_{25}NNaO_5$, 346.1630 $[M + Na]^+$; found, 346.1862.

(2-Nitro-4,5-bis(pentyloxy)phenyl)methanol (S7): To a suspension of aldehyde **S6** (2.5 g, 7.7 mmol) in dry methanol (40 mL) was slowly added $NaBH_4$ (0.58 g, 15 mmol). After being stirred for 15 hours at room temperature, the reaction mixture was concentrated. The residue was dissolved in dichloromethane and washed with water. The aqueous layer was extracted two-times with dichloromethane. The combined organic layer was dried over Na_2SO_4 and concentrated. The residue was purified by silica gel column chromatography eluted by hexane, followed by 12→24% ethyl acetate/hexane, to afford nitrobenzyl alcohol **S7** (2.1 g, 84% yield) as brown solid. ^{1}H NMR (600 MHz, $CDCl_3$): δ 7.65 (s, 1H, Ph-CH), 7.10 (s, 1H, Ph-CH), 4.89 (d, J = 6.0 Hz, 2H, -CH_2OH), 4.10–4.01 (m, 4H, -OCH_2- x2), 4.28 (br, 1H, OH), 1.87–1.81 (m, 4H, -CH_2- x2), 1.47–1.36 (m, 8H, -CH_2- x4), 0.93–0.90 (m, 6H, -CH_2CH_3 x2) ppm. ^{13}C NMR (151 MHz, $CDCl_3$): δ 154.23, 147.81, 139.52, 132.19, 112.21, 109.86, 69.65, 69.51, 62.97, 28.70, 28.67, 28.17, 28.14, 22.48, 22.45, 14.07 ppm. ESI-TOF-MS calcd. for $C_{17}H_{27}NNaO_5$, 348.1787 $[M + Na]^+$; found, 348.1994.

4,5-bis(pentyloxy)nitrobenzyl phosphoramidite (5): To a solution of nitrobenzylalcohol **S7** (350 mg, 1.1 mmol) in dry THF (5.0 mL) was added triethylamine (1.2 mL, 870 mg, 8.6 mmol) and 2-Cyanoethyl-*N*,*N*'-diisopropylchlorophosphoramidite (0.94 mL, 1.0 g, 4.3 mmol). After being stirred for 6 hours at room temperature, the reaction mixture was diluted with ethyl acetate and washed two-times with aqueous solution of *sat.* $NaHCO_3$. The organic layer was dried over Na_2SO_4 and concentrated. The residue was purified by silica gel column chromatography eluted by hexane containing 1% triethylamine, followed by 2→6% ethyl acetate/hexane

containing 1% triethylamine to afford corresponding phosphoramidite **5** (340 mg, 60% yield) as yellow oil. ^{1}H NMR (600 MHz, CD_3CN): δ 7.64 (s, 1H, Ph-CH), 7.28 (s, 1H, Ph-CH), 5.06–4.99 (m, 2H, -C$\underline{H}_2$OP), 4.10–4.00 (m, 4H, -OC$\underline{H}_2$- x2), 3.87–3.75 (m, 2H, -$OCH_2C\underline{H}_2CN$), 3.70–3.64 (m, 2H, -$OC\underline{H}_2CH_2CN$), 2.64–2.62 (m, 1H, CH of *i*-Pr), 1.92–1.91 (m, 1H, CH of *i*-Pr), 1.80–1.73 (m, 4H, -CH_2- x2), 1.44–1.33 (m, 8H, -CH_2- x4), 1.19–1.17 (m, 12H, C$\underline{H}_3$ of *i*-Pr), 0.91–0.86 (m, 6H, -$CH_2C\underline{H}_3$ x2) ppm. ^{13}C NMR (151 MHz, CD_3CN): δ 153.78, 147.33, 138.93, 130.88, 130.82, 118.52, 110.90, 109.64, 69.39, 69.23, 62.69, 62,56, 58.69, 58.55, 43.23, 43.16, 28.53, 28.46, 27.97, 27.90, 24.16, 24.11, 23.96, 23.91, 22.20, 20.16, 20.11, 13.42 ppm. ^{31}P NMR (243 MHz, CD_3CN): δ 149.03 ppm. ESI-TOF-MS calcd. for $C_{26}H_{45}N_3O_6P$, 526.3041 $[M + H]^+$; found, 526.3320; calcd. for $C_{26}H_{44}N_3NaO_6P$, 548.2865 $[M + Na]^+$; found, 548.3153.

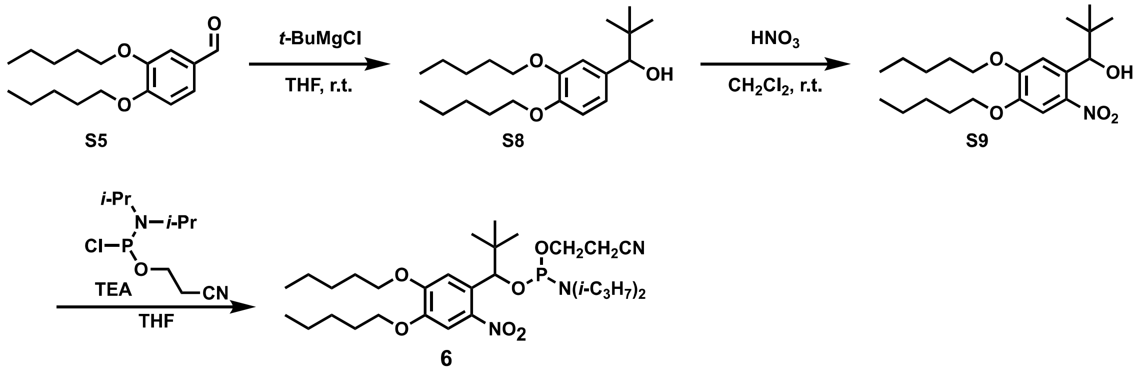

Scheme S4. Synthesis of Compound **6**.

1-(3,4-bis(pentyloxy)phenyl)-2,2-dimethylpropan-1-ol (S8): *tert*-Butylmagnesium chloride solution 1.0 M in THF (23.71 mL, 23.71 mmol) was diluted with 60 mL super dehydrated THF and keep stirring for around 20 minutes at around 0 °C using an ice-salt bath under Ar. Then 3,4-bis(pentyloxy)benzaldehyde (**S5**) (6.00 g, 21.55 mmol) dissolved in 10 mL super dehydrated THF solution was added into the reaction mixture dropwise and keep stirring for around 45 minutes at around 0 °C. Then took off the ice-salt bath, warmed up the experiment to room temperature and continue stirring for 5 hours. After starting material disappeared on TLC, stopped the reaction and added 60 mL aqueous solution *sat.* NH_4Cl. The mixture solvent was extracted by ethyl acetate and washed two times with brine. The organic layer was dried over Na_2SO_4 and concentrated. The residue was purified by silica gel column chromatography on neutral silica gel column using the eluent gradient of hexane/toluene [from 3:1 (v/v) to 1:1 (v/v)] to afford the target compound. Finally, fractions containing the target product were collected, evaporated, and dehydrated under vacuum. Target product 1-(3,4-bis(pentyloxy)phenyl)-2,2-dimethylpropan-1-ol (**S8**) was obtained with yield of 51%, 3.70 g. ^{1}H NMR (400 MHz, $CDCl_3$): δ 6.85-6.76 (m, 3H, Ph-CH), 4.30 (s, 1H, CH of benzyl), 4.02-3.91 (m, 4H, -OC$\underline{H}_2$- x2), 1.84-1.76 (m, 4H, -C$\underline{H}_2$- x2), 1.54-1.19 (m, 9H, -C$\underline{H}_2$- x2 and OH), 1.01-0.85 (m, 15H, -$CH_2C\underline{H}_3$ x2, CH_3 of *t*-Bu x2) ppm. ^{13}C NMR (101 MHz, $CDCl_3$): δ 148.45, 148.33, 135.13, 120.20, 113.70, 112.91, 82.23, 77.47, 76.83, 69.39, 69.32, 35.75, 29.13, 28.33, 26.09, 22.59, 14.14 ppm. ESI-TOF-MS calcd. for $C_{21}H_{36}NaO_3^+$, 359.2562 $[M + Na]^+$; found, 359.2709.

2,2-dimethyl-1-(2-nitro-4,5-bis(pentyloxy)phenyl)propan-1-ol (S9): 1-(3,4-bis(pentyloxy)phenyl)-2,2-dimethylpropan-1-ol (**S8**) (4.50 g, 13.37 mmol) was first mixed with 100 mL CH_2Cl_2 solution and keep stirring for around 20 minutes at around 0 °C using an ice-salt bath under Ar. HNO_3 solution (~70% purity, 2.23 mL, 53.49 mmol) was added into the reaction

mixture dropwise and then took off the ice-salt bath. Kept the reaction mixture stirring at room temperature for 21.5 hours. After starting material disappeared on TLC, stopped the reaction and added 50 mL water. The mixture solvent was extracted by CH_2Cl_2 and washed two times with brine. The organic layer was dried over Na_2SO_4 and concentrated. The residue was purified by silica gel column chromatography on neutral silica gel column using the eluent gradient of toluene/CH_2Cl_2 [from 3:1 (v/v) to 1:1 (v/v)] to afford the target compound. Finally, fractions containing the target product were collected, evaporated, and dehydrated under vacuum. Target product 2,2-dimethyl-1-(2-nitro-4,5-bis(pentyloxy)phenyl)propan-1-ol (**S9**) was obtained with yield of 77%, 3.95 g. ^{1}H NMR (400 MHz, $CDCl_3$): δ 7.41 (s, 1H, Ph-CH), 7.18 (s, 1H, Ph-CH), 5.59 (s, 1H, CH of benzyl), 5.29 (s, 1H, OH), 4.12-4.00 (m, 4H, -OC$\underline{H}_2$- x2), 1.87-1.78 (m, 4H, -C$\underline{H}_2$- x2), 1.49-1.34 (m, 8H, -C$\underline{H}_2$- x4), 0.94-0.84 (m, 15H, -$CH_2C\underline{H}_3$ x2, CH_3 of *t*-Bu x2) ppm. ^{13}C NMR (101 MHz, $CDCl_3$): δ 152.23, 147.39, 141.43, 131.67, 112.35, 109.01, 77.48, 76.83, 74.08, 69.47, 69.31, 53.54, 37.05, 28.73, 28.19, 25.72, 22.49, 14.22, 14.06 ppm. ESI-TOF-MS calcd. for $C_{21}H_{35}NNaO_5^+$, 404.2413 [M + Na]$^+$; found, 404.2754.

4,5-bis(pentyloxy) *t*-Bu-nitrobenzyl phosphoramidite (6): 2,2-dimethyl-1-(2-nitro-4,5-bis(pentyloxy)phenyl)propan-1-ol (**S9**) (1.00 g, 2.62 mmol) was first mixed with 20 mL super dehydrated THF solution and keep stirring under Ar. Then triethylamine (TEA) (3.29 mL, 23.59 mmol) and 2-cyanoethyl *N*,*N*-diisopropylchlorophosphoramidite chloride (2.63 mL, 11.8 mmol) were added into the reaction mixture in turn. Kept the reaction mixture stirring at room temperature for 6 hours. After starting material disappeared on TLC, stopped the reaction. The mixture solvent was extracted by ethyl acetate and washed two times with aqueous solution of *sat.* $NaHCO_3$. The organic layer was dried over Na_2SO_4 and concentrated. The residue was purified by silica gel column chromatography on neutral silica gel column using the eluent gradient of hexane/toluene [from 5:1 (v/v) to 3:1 (v/v)] to afford the target compound. Finally, fractions containing the target product were collected, evaporated, and dehydrated under vacuum. Target product 2-cyanoethyl (2,2-dimethyl-1-(2-nitro-4,5-bis(pentyloxy)phenyl)propyl) diisopropylphosphoramidite (**6**) was obtained with yield of 65%, 0.99 g. ^{1}H NMR (400 MHz, $CDCl_3$): δ 7.42 (s, 1H, Ph-CH), 7.13 (d, J = 9.5 Hz, 1H Ph-CH), 5.57-5.52 (m, 1H, CH of benzyl), 4.09-3.98 (m, 4H, -OC$\underline{H}_2$- x2), 3.91-3.74 (m, 1H, -$OCH_2C\underline{H}_2CN$), 3.69-3.47 (m, 3H, -$OCH_2C\underline{H}_2CN$ and -$OC\underline{H}_2CH_2CN$), 2.68-2.61 (m, 1H, CH of *i*-Pr), 2.37-2.22 (m, 1H, CH of *i*-Pr), 1.92-1.90 (m, 6H, -C$\underline{H}_2$-), 1.79-1.71 (m, 4H, -C$\underline{H}_2$-), 1.47-1.31 (m, 10H, -C$\underline{H}_2$-), 1.17 (t, J = 7.1 Hz, 12H, CH_3 of *i*-Pr), 0.91-0.84 (m, 15H, -$CH_2C\underline{H}_3$ x2, CH_3 of *t*-Bu x2) ppm. ^{13}C NMR (101 MHz, $CDCl_3$): δ 170.70, 152.12, 147.68, 130.39, 129.97, 117.34, 113.51, 113.25, 109.02, 76.71, 75.57, 75.42, 69.31, 69.12, 69.04, 60.02, 59.04, 58.83, 43.35, 43.22, 43.17, 43.04, 37.13, 28.46, 27.95, 27.92, 25.40, 25.25, 24.22, 24.14, 23.98, 23.91, 23.85, 23.78, 22.17, 20.22, 13.59, 13.41 ppm. ^{31}P NMR (243 MHz, CD_3CN): δ 151.77, 148.11 ppm. ESI-TOF-MS calcd. for $C_{30}H_{52}N_3PNaO_6^+$, 604.3491 [M + Na]$^+$; found, 604.3533.

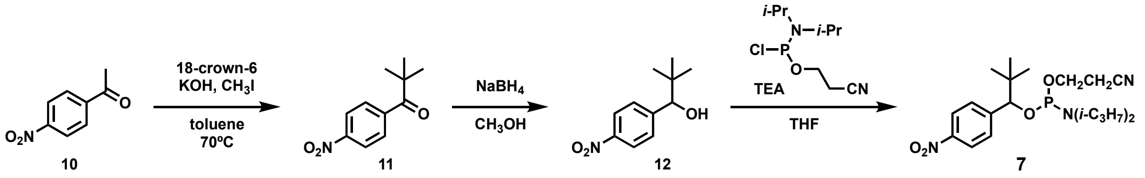

Scheme S5. Synthesis of Compound **7**.

2,2-Dimethyl-1-(4-nitrophenyl)propan-1-ol (11): To a solution of 4-nitroacetophenone (**10**)

(1.00 g, 6.06 mmol) in dry toluene (15.1 mL) was added 18-crown-6 (160 mg, 0.606 mmol) under an argon atmosphere. Potassium hydroxide (2.72 g, 48.4 mmol) and iodomethane (3.02 mL, 48.4 mmol) were added successively to the mixture. After being stirred at 70 °C for 19 hours, the reaction mixture was cooled to room temperature, and quenched by *sat.* NH_4Cl *aq*. (50 mL). The mixture was extracted with EtOAc (50 mL). The organic layer was dried over Na_2SO_4 and concentrated. The residue was purified by silica gel column chromatography (Column size: Φ =2.7 cm, h = 14.5 cm, Gradient: EtOAc/hexane = 1% (5 min.), 1-4% (60 min.), 4% (25 min.), Flow rate: 20 mL/min., Sample load: dry method) to afford compound **11** (659 mg, 3.18 mmol, 53%) as yellow solid. ^{1}H-NMR (400 MHz, $CDCl_3$) δ 8.25 (dt, J = 9.1, 2.1 Hz, 2H, Ph-CH), 7.73 (dt, J = 9.1, 2.1 Hz, 2H, Ph-CH), 1.33 (s, 9H, CH_3 of *t*-Bu) ppm. ^{13}C-NMR (151 MHz, $CDCl_3$) δ 208.6, 148.8, 144.7, 128.4, 123.5, 44.6, 27.6 ppm HRMS (ESI) calcd. for $C_{11}H_{13}NNaO_3^+$, 230.0788 [M + Na]$^+$; found, 230.0784 .

2,2-dimethyl-1-(4-nitrophenyl)propan-1-ol (12): Compound **11** (1.05 g, 5.00 mmol) was dissolved in MeOH (15.0 mL). $NaBH_4$ (189 mg, 5.00 mmol) was added to the mixture and stirred at room temperature for 3 hours. After completion of the reaction, saturated NH_4Cl aq. was added to the reaction mixture and extract with EtOAc. The solution was washed with water and brine, and the organic layer was dried over Na_2SO_4. The filtrate was evaporated and purified by column chromatography on silica gel (hexane/ethyl acetate = 15/1 to hexane/ethyl acetate = 4/1) to get compound **12** (155 mg, 0.739 mmol, 56.1%) as yellow soild. ^{1}H-NMR (600 MHz, $CDCl_3$) δ 8.19 (dt, J = 9.1, 2.2 Hz, 2H, Ph-CH), 7.50 (dt, J = 9.1, 2.0 Hz, 2H, Ph-CH), 4.52 (s, 1H,CH of benzyl), 2.06 (d, J = 1.7 Hz, 1H, OH), 0.94 (s, 9H, CH_3 of *t*-Bu) ppm. ^{13}C-NMR (151 MHz, $CDCl_3$) δ 149.5, 147.3, 128.5, 122.8, 81.6, 36.0, 25.8 ppm. HRMS (ESI) *m/z* calculated for, [M-H]$^-$: 208.0974, found for [M-H]$^-$:208.0978.

***t*-Bu-*p*-nitrobenzyl phosphoramidite (7):** Compound **12** (100 mg, 0.480 mmol) was azeotroped with benzene and dissolved to anhydrous THF (1.90 mL). Triethylamine (0.540 mL, 3.89 mmol) was added 2-cyanoethyl diisopropylchlorophosphoramidite (0.440 mL, 1.92 mmol) was added to the solution, and the mixture was stirred at room temperature for 2 hours. After stirring the mixture, $NaHCO_3$ aq. was added for quench and extract with EtOAc. The solution was washed with water and brine, and organic layer was dried over Na_2SO_4. The filtrate was evaporated *in vacuo* and purified by column chromatography on silica gel (hexane/ethyl acetate = 20/1 +1% TEA to hexane/ethyl acetate = 4/1 +1% TEA) to get desired compound **7** (0.1621 g, 0.3959 mmol, 82.8%) as yellow oil. ^{1}H-NMR (400 MHz, $CDCl_3$) δ 8.14-8.19 (m, 2H, Ph-CH), 7.43-7.49 (m, 2H, Ph-CH), 4.52-4.56 (m, 1H, CH of benzyl), 3.42-3.91 (m, 4H, -O$\underline{CH_2}\underline{CH_2}$CN), 2.19-2.68 (m, 2H, CH of *i*-Pr), 0.98-1.27 (m, 12H, CH_3 of *i*-Pr), 0.91 (d, J = 6.8 Hz, 9H, CH_3 of *t*-Bu), ^{13}C-NMR (101 MHz, $CDCl_3$) δ 148.77, 148.53, 147.26, 147.11, 129.26, 129.16, 122.65, 122.50, 117.77, 117.50, 84.38, 84.24, 83.01, 82.89, 58.33, 58.12, 57.80, 57.58, 43.44, 43.31, 43.27, 43.15, 36.34, 36.29, 36.20, 26.18, 26.08, 24.81, 24.71, 24.64, 24.42, 24.34, 20.63, 20.55, 20.30, 20.23, ^{31}P-NMR (162 MHz, $CDCl_3$) δ 152.18, 147.97. ESI-TOF-MS calcd. for $C_{20}H_{32}N_3NaO_4P^+$, 432.2023 [M + Na]$^+$; found, 432.2037.

2-2. Synthesis of RpGs

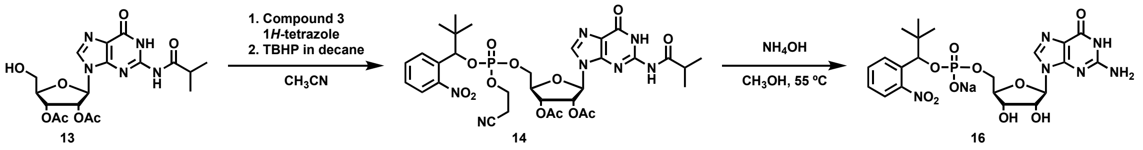

Scheme S6. Synthesis of RpG **16**.

Protected-(*o*-nitrobenzyl)RpG (14): Compound **3** (1.08 mg, 2.63 mmol), Compound **13** (4) (690 mg, 1.58 mmol), and Molecular Sieves 3A (2.00 g) were suspended in 15.0 mL acetonitrile. After being stirred at room temperature for 20 minutes, 1*H*-tetrazole (373 mg, 3.95 mmol) was added, and the solution was stirred for 4 hours. Then, 5 M TBHP in decane (0.789 mL, 3.95 mmol) was added to the reaction mixture. After being stirred at room temperature for 1 hour, the reaction mixture was quenched by the addition of mili-Q water and extracted with dichloromethane. The organic layer was washed with water and brine, dried over Na_2SO_4. The filtrate was evaporated *in vacuo* and purified by column chromatography on silica gel (Dichloromethane/methanol = 99/1 to 10/1) to afford compound **14** (846 mg, 1.11 mmol, 42.2%) as yellow solid. ^{1}H-NMR (600 MHz, DMSO-D6) δ 12.12-12.07 (m, 1H, NH), 11.61-11.48 (m, 1H, NH), 8.24-8.16 (m, 1H, Ph-CH), 7.95-7.18 (m, 4H, Ph-CH and CH-8), 6.13-6.03 (m, 1H, CH-1′), 5.90-5.84 (m, 1H, CH-2′), 5.82-5.70 (m, 1H, CH-3′), 5.52-5.35 (m, 1H, CH-4′), 4.53-3.93 (m, 5H, 5′, -OC$\underline{H}_2$CH$_2$CN and CH of benzyl), 2.96-2.65 (m, 3H, -OCH$_2$C$\underline{H}_2$CN, CH of *i*-Pr), 2.24-1.89 (m, 6H CH_3 of acetyl), 1.13-1.09 (m, 6H, CH_3 of *i*-propyl), 0.89-0.77 (m, 9H, CH_3 of *t*-Bu) ppm. ^{13}C-NMR (151 MHz, DMSO-D6) δ 180.6, 170.0, 169.9, 169.9, 169.7, 169.7, 169.6, 155.2, 149.1, 149.0, 149.0, 148.9, 148.8, 138.3, 133.4, 133.3, 131.7, 130.5, 130.3, 130.1, 130.0, 129.9, 129.9, 126.0, 124.9, 124.9, 124.8, 121.0, 120.4, 118.8, 118.5, 118.5, 118.3, 84.9, 81.2, 81.2, 81.1, 80.3, 72.5, 72.4, 70.5, 67.4, 67.2, 63.4, 63.3, 63.3, 63.1, 59.3, 58.7, 37.0, 37.0, 35.4, 29.8, 29.4, 25.5, 25.0, 20.9, 20.7, 19.6, 19.5, 19.4, 19.4, 19.4, 19.3, 19.2 ppm. ^{31}P-NMR (243 MHz, DMSO-D6) δ -1.6, -1.8, -5.9, -6.5 ppm. HRMS (ESI) *m/z* calculated for, $[M – H]^-$: 761.2422, found for $[M – H]^-$:760.2370.

***o*-nitrobenzyl-RpG (16):** Compound **14** (846 mg, 1.11 mmol) was dissolved in MeOH (11 mL) and 28% NH_3 aq. (11 mL) was added to the solution. The mixture was stirred for 16 hours at 55 °C. The solvent was evaporated *in vacuo* and dissolved in water. The solution was washed with CH_2Cl_2. The aqueous layer was purified by DEAE-Sephadex TM A-25 column (particle size: 40–100 μm, column size: φ = 4.5 cm, *h* = 8.4 cm (140 cm^3), flow rate: 12 mL/min, solvent A: miliQ-water, solvent B: 1.5 M TEAB buffer + 10% acetonitrile, gradient: 0–40%B over 180 minutes). After purification, the fractions including desired compound was purified by Wakosil® 25C18 column (particle size: 15–30 μm (spherical), column size: φ = 4.80 cm, *h* = 10.2 cm (184 cm^3), flow rate: 12 mL/min, solvent A: 50 mM TEAA + 5% acetonitrile, solvent B: acetonitrile, gradient: 0–90% over 100 minutes). After purification, the solution was evaporated *in vacuo* to get desired compound as yellow solid (Counter cation: triethylammonium). The compound was dissolved in 2mL MeOH, and 40 mL 0.14 M $NaClO_4$ in acetone was added, and centrifuged at 4,500 rpm for 10 minutes. This operation was repeated three times. Compound **16** as sodium salt was obtained as yellow solid (512 mg, 0.803 mmol, 72.3% yield). ^{1}H-NMR (600 MHz, DMSO-D6) δ 10.66 (s, 1H, NH), 7.89-7.43 (m, 5H, Ph-CH and CH-8), 6.65 (2s, 2H, NH_2), 5.64 (d, J = 9.2 Hz, 1H, CH of *t*-Bu), 5.59 (dd, J = 5.9, 1.2 Hz, 1H, CH-1), 5.45 (m, 1H, OH-2′), 5.34 (t, J = 5.3 Hz, 1H, OH-3′), 4.37 (m, 1H, CH-2′), 3.97 (m, 1H, CH-3′), 3.72 (m, 1H, CH-4′), 3.64-3.45 (m, 2H, CH_2-5′), 0.80 (s, 9H, CH_3

of *t*-Bu) ppm. ^{13}C-NMR (151 MHz, DMSO-D6) δ 207.1, 157.4, 154.2, 151.9, 149.3, 136.4, 136.2, 132.5, 130.9, 128.3, 124.1, 117.1, 87.0, 76.3, 74.2, 74.1, 71.4, 71.3, 64.8, 36.9, 31.2, 26.3 ppm. ^{31}P-NMR (243 MHz, DMSO-D6) δ -0.2, -0.3, -0.4 ppm. HRMS (ESI) *m/z* calculated for, $[M-H]^-$: 553.1454, found for $[M-H]^-$:553.1481.

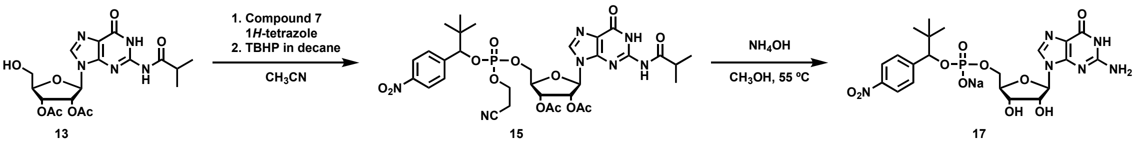

Scheme S7. Synthesis of RpG **17**.

Protected-(*p*-nitrobenzyl)RpG (15): Compound **7** (1.08 mg, 2.63 mmol), Compound **13** (4) (690 mg, 1.58 mmol), and Molecular Sieves 3A (2 g) were suspended in acetonitrile (15.0 mL). The mixture was stirred for 20 minutes at room temperature. 1*H*-Tetrazole (373 mg, 3.95 mmol) was added to the mixture and stirred for 4 hours. Then, 5 M TBHP in decane (0.789 mL, 3.95 mmol) was added to the mixture. After being stirred at room temperature for 1 hour, the reaction mixture was quenched by the addition of mili-Q water and extracted with dichloromethane. The organic layer was washed with water, brine, and dried over Na_2SO_4. The filtrate was evaporated *in vacuo* and purified by column chromatography on silica gel (Dichloromethane/methanol = 99/1 to 10/1) to afford compound **15** (846 mg, 1.11 mmol, 42.2%) as yellow soild. ^{1}H-NMR (600 MHz, DMSO-D6) δ 12.10 (d, J = 12.6 Hz, 1H, NH), 11.57-11.53 (m, 1H, , NH), 8.24-8.09 (m, 3H, Ph-CH, and CH-8), 7.61-7.53 (m, 2H, Ph-CH), 6.12-6.01 (m, 1H, CH-1′), 5.80-5.69 (m, 2H, CH-1′, CH of *t*-Bu), 5.47-5.33 (m, 1H, CH-2′), 5.26-5.20 (m, 1H, CH-3′), 4.40-3.94 (m, 5H, CH-4′, CH_2-5′and -OC<u>H</u>$_2$$CH_2$CN), 2.90-2.70 (m, 3H, -$OCH_2$C<u>H</u>$_2$CN and, CH of *i*-Pr), 2.13-2.00 (m, 6H), 1.14-1.11 (m, 6H, CH_3 of acetyl), 0.91-0.83 (m, 9H, CH_3 of *t*-Bu) ppm. ^{13}C-NMR (101 MHz, DMSO-D6) δ 180.6, 170.0, 169.9, 169.7, 155.2, 149.2, 148.9, 147.6, 147.5, 145.6, 145.5, 138.3, 138.1, 129.3, 129.3, 123.3, 123.2, 123.1, 121.0, 118.6, 118.5, 86.5, 86.5, 86.4, 84.9, 84.8, 84.6, 81.3, 81.2, 81.1, 72.5, 72.4, 72.4, 70.6, 70.5, 67.3, 63.2, 63.0, 36.2, 36.1, 35.3, 25.7, 20.9, 20.6, 19.5, 19.4, 19.4, 19.3, 19.3 ppm. ^{31}P-NMR (243 MHz, DMSO-D6) δ -1.7, -1.8, -1.8 ppm. $[M-H]^-$: 761.2422, found for $[M-H]^-$:760.2370

***p*-nitrobenzyl-RpG (17):** Compound **15** (846 mg, 1.11 mmol) was dissolved in MeOH (11 mL) and 28% NH_3 aq. (11 mL) was added to the solution. The mixture was stirred for 16 hours at 55 °C. After stirring, the mixture was evaporated *in vacuo* and washed by CH_2Cl_2. The water layer was purified by DEAE-Sephadex TM A-25 column (particle size: 40–100 μm, column size: φ = 4.5 cm, *h* = 8.4 cm (140 cm^3), flow rate: 12 mL/min, solvent A: miliQ-water, solvent B: 1.5 M TEAB buffer + 10% acetonitrile, gradient: 0–40%B over 180 minutes). After purification, the fractions including desired compound was purified by Wakosil® 25C18 column (particle size: 15–30 μm (spherical), column size: φ = 4.80 cm, *h* = 10.2 cm (184 cm^3), flow rate: 12 mL/min, solvent A: 50 mM TEAA + 5% acetonitrile, solvent B: acetonitrile, gradient: 0–90% over 100 minutes). After purification, the solution was e*vaporated in vacuo* to get desired compound as yellow solid (Counter cation: triethylammonium). The compound was dissolved in 2mL MeOH, and 40 mL 0.14 M $NaClO_4$ in acetone was added, and centrifuged at 4,500 rpm for 10 minutes. This operation was repeated three times. Compound **17** was obtained as yellow solid (512 mg, 0.803 mmol, 72.3%). ^{1}H-NMR (600 MHz, DMSO-D6) δ 10.72 (s, 1H, NH), 8.15-8.08 (m, 2H, Ph-CH), 7.91-7.86 (m, 1H, CH-8), 7.52 (dd, J = 11.6, 8.8 Hz, 2H, , Ph-CH), 6.60 (s, 2H, NH_2 of 2), 5.61 (m,

1H, OH-2′), 5.38-5.28 (m, 2H, OH-3′and CH of *t*-Bu), 4.86 (dd, J = 9.3, 3.2 Hz, 1H, CH-1′), 4.39-4.32 (m, 1H, CH-2′), 3.88 (m, 1H, CH-3′), 3.79-3.58 (m, 3H, CH-4′, CH_2-5′), 0.84 (d, J = 4.4 Hz, 9H, CH of *t*-Bu) ppm. ^{13}C-NMR (151 MHz, DMSO-D6) δ 207.1, 157.4, 154.2, 151.9, 149.3, 136.4, 136.2, 132.5, 130.9, 128.3, 124.1, 117.1, 87.0, 76.3, 74.2, 74.1, 71.4, 71.3, 64.8, 36.9, 31.2, 26.3 ppm. ^{31}P-NMR (243 MHz, DMSO-D6) δ -0.2, -0.3, -0.4 ppm. HRMS (ESI) *m/z* calculated for, $[M-H]^-$: 553.1454, found for $[M-H]^-$:553.1484.

2-3. Oligo Synthesis, Purification, and Deprotection

Chemical Synthesis of 5’-Phosphorylated DNAs

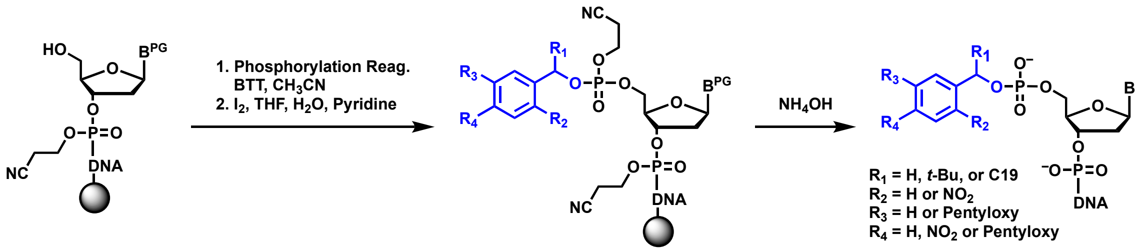

DNAs with hydrophobic protecting group were synthesized on a DNA/RNA synthesizer NR-2A_7MX or NRs-4A_10R7NP (Nihon Techno Service), using DNA phosphoramidites and CPG (Chemgenes; deoxy Guanosine (n-ibu) 3'-lcaa CPG, 1000A T.V. 47.3 µmol/g) and CPR**1–7**. 50 mM DNA phosphoramidite and 100 mM CPR**1–7** solution in acetonitrile were prepared to use these for the DNA synthesis. Reagents for the synthesizer were used as follows: 3 w/v % trichloroacetic acid in dichloromethane for deblocking; 0.25 M 5-benzylthio-1H-tetrazole in acetonitrile (Wako) for coupling; a mixture of acetic anhydride/ tetrahydrofuran/ pyridine (1: 8: 1, Wako) and 10 (v/v)% 1-methylimidazole in tetrahydrofuran (Wako) for capping; 0.01 M iodine in 64% acetonitrile, 6% pyridine, 30% water for oxidation (Honeywell). After the synthesis, DNAs were cleaved from the support and deprotected using a 1: 1 mixture of 40% aqueous methylamine-28% ammonium hydroxide at 65 °C for 15 min or 28% ammonium hydroxide at room temperature for overnight. After cleavage and deprotection, the CPG was removed by filtration, and the filtrate was concentrated. The crude DNAs were purified by reversed-phase HPLC. The purification conditions were as follows: column, YMC Hydrosphere C18, 250 × 10 mm I.D., S-5 µm, 12 nm; Solution A, 50 mM triethylammonium acetate (pH 7.0) containing 5% acetonitrile; Solution B, acetonitrile; typical gradient, 0 to 100% Solution B over 20 min; column temperature, 50 °C; flow rate, 1 mL/min; detection wavelength, 260 nm. After the purification, the DNA was precipitated by isopropanol precipitation (DNA solution: 1.00 mL, 3 M NaOAc aq.: 125 µL, isopropanol: 1.25 mL, 20 mg/mL glycogen aqueous solution: 12.5 µL). The mixture was cooled to –80 ºC for 30 minutes and centrifuged (15,000 rpm, 4 ºC, 15 minutes). The supernatant was removed, and 80% aqueous ethanol (1.0 mL) was added to the pellet, and centrifuged (15,000 rpm, 4 °C, 15 min). The supernatant was removed, and the pellet was dried under reduced pressure. The resulting white solid was dissolved in water. The DNA concentration was determined using the extinction coefficient calculated by the nearest neighbor method using the Oligoanalyzer software from Integrated DNA Technologies, based on the absorbance at 260 nm measured on a NanoDrop2000 spectrometer (Thermo Fisher Scientific). The purity of the DNAs was confirmed by 20% denaturing polyacrylamide gel electrophoresis (dPAGE) containing 7.5 M urea as a denaturant and reverse-phase HPLC. For dPAGE analysis, the ratio of acrylamide and *N*, *N*'-methylenebis(acrylamide) was 19 :1. After

electrophoresis, the gel was stained by SYBR Green II (Lonza) and visualized on the ChemiDoc XRS Plus system (Bio-Rad).

Chemical Synthesis of 5'-Phosphorylated RNAs

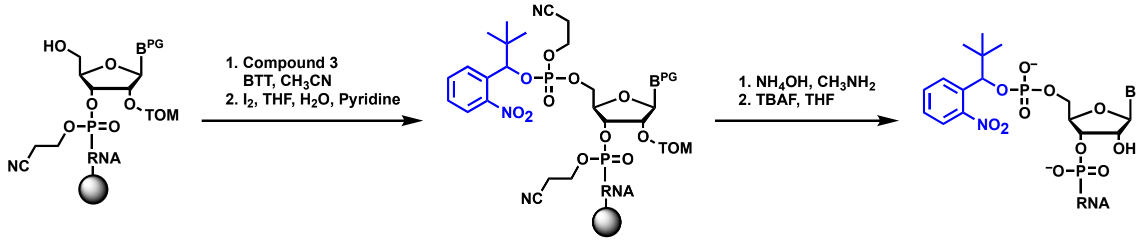

RNAs with nitrobenzyl tag were synthesized in the same way as DNA described above. Cleavage from the support and deprotection were performed by the treatment with a 1:1 mixture of 28% aqueous ammonia and 40% methylamine (1.00 mL) at 65 °C for 15 minutes. After filtration with a membrane filter and centrifugal evaporation, the RNAs were treated with 1 M tetrabutylammonium fluoride in THF (1.0 mL) at room temperature overnight. The reaction mixture was quenched by the addition of 1 M Tris-HCl buffer (pH 7.5, 1.0 mL) and concentrated to remove THF. The solution was desalted using an NAP25 column (Merck), and the RNA was precipitated by isopropanol precipitation (RNA solution: 1.00 mL, 3 M NaOAc aq.: 125 μL, isopropanol: 1.25 mL, 20 mg/mL glycogen aqueous solution: 12.5 μL). The mixture was cooled to –80 ºC for 30 minutes and centrifuged (15,000 rpm, 4 ºC, 15 minutes). The supernatant was removed, and 80% aqueous ethanol (1.0 mL) was added to the pellet, and centrifuged (15,000 rpm, 4 °C, 15 min). The supernatant was removed, and the pellet was dried under reduced pressure. The resulting white solid was dissolved in water. Crude RNAs was purified by RP-HPLC. The purification conditions were as follows: column, YMC Triart Bio C4, 250 × 10 mm I.D., S-5 μm, 12 nm; Solution_A, 50 mM triethylammonium acetate (pH 7.0) containing 5% acetonitrile; Solution_B, acetonitrile; typical gradient, 0 to 20% Solution_B over 20 min; column temperature, 50 °C; flow rate, 1 mL/min; detection wavelength, 260 nm. The RNA concentration and purity were confirmed in the same way as DNA described above.

Deprotection of *o*-Nitrobenzyl Groups by UV-Irradiation

5'-Nitrobenzylphosphate DNA/RNA solutions were added to a transparent 96-well multi-well plate (75 μL/well) and irradiated with 365 nm light at 4 mW/cm^2 for 10 minutes by a MAX-305 light source device (Asahi spectroscopy). After deprotection, the DNA/RNA was precipitated by isopropanol precipitation (DNA/RNA solution: 1.00 mL, 3 M NaOAc aq.: 125 μL, isopropanol: 1.25 mL, 20 mg/mL glycogen aqueous solution: 12.5 μL). The mixture was cooled to –80 ºC for 30 minutes and centrifuged (15,000 rpm, 4 ºC, 15 minutes). The supernatant was removed, and 80% aqueous ethanol (1.0 mL) was added to the pellet, and centrifuged (15,000 rpm, 4 °C, 15 min). The supernatant was removed, and the pellet was dried under reduced pressure. The resulting white solid was dissolved in water. DNA/RNA was analyzed using reverse-phase HPLC and dPAGE. HPLC condition was as follows: case of DNA, column, YMC Hydrosphere C18, 250 × 10 mm I.D., S-5 μm, 12 nm; Solution A, 50 mM triethylammonium acetate (pH 7.0) containing 5% acetonitrile; Solution B, acetonitrile; typical gradient, 0 to 60% Solution B over 20 min; column temperature, 50 °C; flow rate, 1 mL/min; detection wavelength, 260 nm. In Case of RNA, column, YMC Triart Bio C4: Solvent A, 50 mM TEAA (pH 7.0), 5 % ACN; Solvent B, ACN; Gradient 0-100 % B/ 0-20 min; Flow rate, 1 mL/min; Column temperature, 50 °C; Column size 250 x 4.6 mmI.D, S-5 μm, 12 nm; Detection wavelength, 260 nm.

Deprotection of *o*-/*p*-Nitrobenzyl Groups by Reductive Condition

For the deprotection of *o*-nitrobenzyl groups, the 10 μM 5'-nitrobenzylphosphate DNA/RNA solutions (1.0 nmol) in 20 mM Tris-HCl buffer (pH 8.0) containing 100 mM sodium dithionite were incubated at 37 °C for 30 minutes and then 65–95 °C for 10–20 minutes. The reaction time varies depending on the substituents of benzene position (See Table 2). For the deprotection of *p*-nitrobenzyl group, the 10 μM 5'-nitrobenzylphosphate DNA/RNA solutions (1.0 nmol) in 20 mM Tris-HCl buffer (pH 8.0) containing 100 mM sodium dithionite were incubated at 37 °C for 30 minutes and then 55 °C for 2 hours. After deprotection, DNA/RNA were analyzed by reversed-phase HPLC. HPLC condition was as follows: In the case of 19 mer DNA, column: YMC Hydrosphere C18: Solvent A, 50 mM TEAA (pH 7.0), 5 % acetonitrile; Solvent B, ACN; Gradient 0-60 % B/ 0-20 min; Flow rate, 1 mL/min; Column temperature, 50 °C; Column size 250 × 4.6 mmI.D, S-5 μL, 12 nm; Detection wavelength, 260 nm. In the case of transcribed RNA, column: YMC Triart Bio C4: Solvent A, 100 mM TEAA (pH 7.0), 5 % ACN; Solvent B, 100 mM TEAA (pH 7.0), 50% ACN; Gradient 15-30 % B/ 0-25 min; flow rate, 1 mL/min; column temperature, 50 °C; column size 250 x 4.6 mmI.D, S-5 μm, 30 nm; detection wavelength: 260 nm.

2-4. Chemical Synthesis of Minimal mRNA

5'-Phosphate RNA was synthesized using phosphoramidite **3** and purified by reverse-phase HPLC (Column: Hydrosphere C18 (250 x 4.6 mmI.D., S-5 μm, 12 nm), Solvent A 50 mM TEAA buffer (pH 7.0) + 5% CH_3CN, Solvent B: CH_3CN, Gradient: 5-50%B over 20 min, column temperature 50 °C, flow rate: 1 mL/min, detection: 254 nm, loop size: 2.0 mL). After HPLC purification, the 5'-Nitrobenzylphosphate RNA solution was added to a transparent 96-well multi-well plate (75 μL/well) and irradiated with 365 nm light at 4 mW/cm^2 for 10 minutes by a MAX-305 light source device (Asahi spectroscopy). After removal of the nitrobenzyl group, RNA was precipitated by isopropanol precipitation (RNA solution: 1.00 mL, 3 M NaOAc aq.: 125 μL, isopropanol: 1.25 mL, 20 mg/mL glycogen aqueous solution: 12.5 μL). The mixture was cooled to −80 ºC for 30 minutes and centrifuged (15,000 rpm, 4 ºC, 15 minutes). The supernatant was removed, and 80% aqueous ethanol (1.0 mL) was added to the pellet, and centrifuged (15,000 rpm, 4 °C, 15 min). The supernatant was removed, and the pellet was dried under reduced pressure. The resulting white solid was dissolved in water. The RNA pellet was dissolved in water and quantified by NanoDrop. The RNA solution was lyophilized and dissolved in dry dimethyl sulfoxide (DMSO, 127 μL/5 nmol RNA). A 15 mM DMSO solution of 7-methylguanosine 5'-diphosphate-imidazolide (chemical capping reagent, 333 μL/5 nmol RNA) and 1-methylimidazole (40 μL/5 nmol RNA) was added to the RNA solution (5). The mixture was incubated at 55 °C for 4 h, and then RNA was precipitated by isopropanol precipitation (reaction mixture: 500 μL, water: 500 μL, 3 M NaOAc aq.: 125 μL, isopropanol: 1.25 mL, 20 mg/mL glycogen aqueous solution: 12.5 μL). The mixture was cooled to −80 ºC for 30 minutes and centrifuged (15,000 rpm, 4 ºC, 15 minutes). The supernatant was removed, and 80% aqueous ethanol (1.0 mL) was added to the pellet, and centrifuged (15,000 rpm, 4 °C, 15 min). The supernatant was removed, and the pellet was dried under reduced pressure. The resulting white solid was dissolved in water. The crude capped-RNA was purified by RP-HPLC (Column: Hydrosphere C18 (250 x 4.6 mmI.D., S-5 μm, 12 nm), Solvent A: 50 mM TEAA buffer (pH 7.0) + 5% CH_3CN, Solvent B: CH_3CN, Gradient: 5-50%B over 20 minutes, column temperature 50 ºC, Flow rate: 1 mL/min, detection: 254 nm, loop size: 2.0 mL). Characterization of the product was carried out by dPAGE (20 × 20 cm gel, 30 W, 3 h, 2 pmol RNA sample loading, SYBR Green II Stain,

Low range ssRNA Ladder (NEB) as a size marker), and LC-MS.

2-5. Biological activity evaluation of chemically synthesized mRNA

WST8 assay: HeLa cells were cultured and maintained at 37 °C and 5% CO_2 in a humidified incubator in D-MEM medium containing 10% FBS. One day before transfection, the cells were trypsinized and seeded into a 96-multiwell cell culture plates at 6,000 cells per well. The cells were then incubated at 37 °C for 24 h in a 5% CO_2 atmosphere. The mRNA transfection was performed using Lipofectamine® messengerMAX. After incubation at 37 °C for 24 h in a 5% CO_2 atmosphere, the WST8 assay was performed according to general procedures.

Preparation of IVT Template DNA which codes HiBiT peptide: IVT template was prepared by primer extension. The mixture of forward and reverse primers (0.3 µM each), 0.2 mM dNTPs, 1,5 mM $MgSO_4$, 1×PCR Buffer for KOD -Plus- Neo and 0.02 units/µL KOD -Plus- Neo (Toyobo) was subjected to the following thermal conditions: 95 °C for 2 min→65 °C for 10 min. The reaction was analyzed by native PAGE. After that, reaction mixture was purified using Wizard Plus SV Minipreps DNA Purification system (Promega).

Sequence of Forward Primer:

5'-GGATCCTAATACGACTCACTATAAGAGCCACCATGGTGAGCGGCTGGCGGCTG-3'

Sequence of Reverse Primer:

5'-TTTTTTTTTTTTTTTTTTTTTCAGCTAATCTTCTTGAACAGCCGCCAGCCGCTCAC-3'

Preparation of IVT-mRNA which codes HiBiT peptide: The transcription solution was consisted of 5 ng/µL of DNA template (PCR product), 1× buffer, 5 mM of DTT, 2 mM of NTPs, 2 mM of CleanCap-AG (house-made), 0.0002 U/µL of pyrophosphatase (house-made), and 5 U/µL of T7 RNA polymerase (house-made). The transcription solution was incubated at 37 °C for 2 hours, and then DNase I (Takara) was added to the solution and incubated at 37 °C for 30 min. After that, mRNA was purified using Monarch column (New England Biolab, T2040L).

Measurement of translation activity of HiBiT-mRNA in HeLa cells: HeLa cells were cultured and maintained at 37 °C with 5% CO_2 in a humidified incubator in DMEM containing 10% FBS. One day before transfection, the cells were trypsinized and seeded into a 96-multiwell cell culture plate at 1×10^4 cells per well. To transfect RNA into HeLa cells, a mixture of RNA (20, 100 or 200 ng), Lipofectamine MessengerMAX (0.15 µL vs 100 ng of mRNA), and Opti-MEM I (total 10 µL) per well was added to the cells. After four hours of incubation, the medium was removed from the well, and the cells were lysed with 20 µL of Nano-Glo HiBiT Lytic Buffer containing LgBiT Protein solution (0.2 µL) and Nano-Glo® HiBiT Lytic Substrate solution (0.4 µL) which were obtained as a kit Nano-Glo HiBiT Lytic Detection System (Promega). After 10 min incubation, the lysates were transferred to a white 96-multiwell plate and chemiluminescence was measured on plate reader (Berthold).

Preparation of LNP: LNPs were prepared by the vortex mixing method. Briefly, 10 µg of RNA was dissolved in 600 µL of 5 mM Citrate buffer (pH 4). Lipids were dissolved in 200 µL of ethanol with the following lipid composition: SM102/DOPE/Cholesterol/DMG-PEG2k = 50/10/38.5/1.5 molar ratio (Lipid/RNA = 400 nmol/10 µg). Under vortex mixing, the lipid ethanol solution was added into the buffer containing RNA, and then 1 mL of PBS was added. The LNP suspension was centrifuged (25 °C, 1500×g, 30 min) with an Amicon Ultra 100k. LNPs were recovered from the tubes with PBS.

Measurement of translation activity of HiBiT-mRNA in Mice: LNP-formulated mRNAs were injected into C57BL6N-5w-female mice (10 µg/mouse) via the tail vein. Six hours after the

injection, the liver and the spleen were collected. These tissues were homogenized using a Micro Smash MS-100R (TOMY, Japan) in 1 mL of lysis buffer (100 mM Tris-HC, 2 mM EDTA, 0.1% Triton X-100, pH = 7.8). The homogenate was centrifuged (15,000 rpm, 4 °C, 10 min) and the supernatant was collected. HiBiT expression was measured using a HiBiT Lytic assay system (Promega). Briefly, a 30 μL of the supernatant was mixed with 60 μL of the solution containing LgBiT protein and the substrate, and the luminescence was measured using a luminometer (GloMax, Promega). The total protein amount in the supernatant was quantified by BCA assay. HiBiT expressions were expressed as RLU per mg of protein.

2-6. *In Vitro* Transcribed Synthesis of RNA by Using RpGs

2-6-1. Preparation of EMCV-IRES-pNL1.1TK plasmid

Preparation of EMCV-IRES Containing DNA Fragment: To 500 ng of pIRES Vector (Takara) containing encephalomyocarditis virus (EMCV)-derived IRES, 1 μL of 10 × K buffer, 0.5 μL of EcoR I (Takara), and 0.5 μL of Bam HI (Takara) were added, and MQ-water was added to make the total volume 10 μL. It was then incubated at 37°C for 1 hour. Progress of the restriction enzyme reaction was confirmed by 1% agarose electrophoresis (100 V, 30 minutes, 1×TAE). Since the 645 bp sequence fragment contained IRES, that band was isolated and purified using the Wizard Plus SV Minipreps DNA Purification system (Promega).

Preparation of NanoLuc-Coding DNA Fragment: To the PCR reaction mixture (5 μL of 10x buffer, 3 μL of 25 mM $MgSO_4$, 5 μL of 2 mM dNTPs, 1 μL of KOD plus neo), 10 ng of pNL1.1TK vector (Nluc/CMV, Promega), 10 μM forward PCR primer (5'-CCACAACCCGGCCACCATGGTCTTCACACTCG-3', 25 pmol) and 10 μM reverse PCR primer (5'-CTCGACGCGTGAATTACGCGTCACCTTAATATGCG-3', 25 pmol) were added (prepared the solution to 50 μL total volume). PCR was performed under the following conditions: 94°C for 2 minutes → (98°C for 10 seconds, 55°C for 30 seconds, 68°C for 3 minutes) × 25 → 68°C for 3 minutes → 4°C. The amplification product was confirmed by 1% agarose electrophoresis (100V, 30 minutes, 1×TAE). To digest the plasmid, 0.5 μL of the restriction enzyme Dpn I (Takara) was added to 48 μL of the PCR reaction mixture, and incubated at 37° C for 1 hour. The reaction solution was purified using the Wizard Plus SV Minipreps DNA Purification system (Promega).

In-Fusion Reaction to Prepare EMCV-IRES-pNL1.1TK Plasmid: 2.5 μL of IRES fragment (13.5 ng/μL), 1.5 μL of the linear vector containing NanoLuc luciferase (31.1 ng/μL), and 1 μL of 5 × In-fusion HD Enzyme Premix (Takara), were mixed. As a negative control sample, a sample was also prepared by taking 1.5 μL of a linear vector containing NanoLuc luciferase (31.1 ng/μL), 1 μL of 5 × In-fusion HD Enzyme Premix (Takara), and 2.5 μL of MQ-water. These mixtures were incubated at 50°C for 15 minutes. After performing the ECOS 6 minutes protocol, 50 μL of ECOS JM109 competent cells was added. It was applied to an LB/agar medium (containing 100 μL/mL ampicillin) and incubated overnight in a 37°C incubator. After 15 hours, it was confirmed that colonies had formed, and a single colony was added to 10 mL of LB/ampicillin liquid medium. Then, it was shaken overnight at 37°C. Thereafter, plasmid extraction was performed using the Wizard Plus SV Minipreps DNA Purification system (Promega). Concentration was measured by absorbance at 260 nm.

2-6-2. Preparation of Template DNA by PCR

The PCR mixture was consisted of 0.3 μM primers, 1 ng/μL pNL1.1TK vector (for circular

mRNA without IRES) or EMCV-IRES-pNL1.1TK plasmid (for circular mRNA with IRES), 0.2 mM dNTPs, 1.5 mM $MgSO_4$, 1×PCR Buffer for KOD -Plus- Neo, 0.02 units/μL KOD -Plus- Neo (Toyobo). The mixture was subjected to the following thermal cycling conditions: 95 °C for 2 min → (98 °C for 10 s → 55 °C for 30 s → 72 °C for 1 min) × 30 cycles → 72 °C for 5 min. The reaction was analyzed by 1% agarose gel electrophoresis (100 V, 30 minutes, 1x TAE). After the gel electrophoresis, The PCR product was purified using a wizard® SV gel and PCR Clean-up System (Promega) according to the manufacturer's recommended protocol.

2-6-3. *In vitro* transcription by using RpGs

The transcription solution was consisted of 20 ng/μL DNA template (PCR product), 10 mM DTT, 24 mM $MgCl_2$, 7.5 mM ATP, CTP, UTP, 1.9 mM, GTP, 7.5 mM *o*-Nb-RpG (**16**) or *p*-Nb-RpG (**17**), 25 U/μL T7 RNA polymerase (house-made), 0.004 U/μL pyrophosphatase (New England Biolab, N2403L), 0.2 U/μL RNase inhibitor). The transcription solution was incubated at 37 °C for 2 hours, and then DNase I (Takara) was added to the transcript solution and incubated at 37°C for 30 min to remove any remaining DNA transcription template. Next, a 1:1 mixture of TE-saturated phenol and chloroform was added, and the mixture was centrifuged. Chloroform was then added to the water layer and centrifuged. The water layer was isolated and RNA was precipitated by isopropanol precipitation (RNA solution: 1.00 mL, 3 M NaOAc aq.: 125 μL, isopropanol: 1.25 mL, 20 mg/mL glycogen aqueous solution: 12.5 μL). The mixture was cooled to –80 ºC for 30 minutes and centrifuged (15,000 rpm, 4 ºC, 15 minutes). The supernatant was removed, and 80% aqueous ethanol (1.0 mL) was added to the pellet, and centrifuged (15,000 rpm, 4 °C, 15 min). The supernatant was removed, and the pellet was dried under reduced pressure. The resulting white solid was dissolved in water. The RNA solution was purified using reverse-phase HPLC. The condition was as follows: YMC-Triart C4: Solvent A, 100 mM TEAA (pH 7.0), 5 % acetonitrile; Solvent B, 100 mM TEAA (pH 7.0), 50 % acetonitrile; Gradient 15-30% B/ 0-20 min; Flow rate, 1 mL/min; Column temperature, 50 °C; Column size 250 x 4.6 I. D., S-5 μm, 30 nm; Detection wavelength, 260 nm.

2-7. Synthesis and Evaluation of Circular mRNA

2-7-1. Circularization reaction

The annealing solution was consisted of 1.0 μM Splint DNA, 0.5 μM linear RNA, T4 RNA ligase 2 buffer (50 mM Tris-HCl, 2 mM $MgCl_2$, 1 mM DTT, 400 μM ATP). The solution was heated at 90 °C for 3 min and then gradually cooled to room temperature. PEG 8000 was added to the solution (10% final concentration), followed by T4 RNA ligase 2 (house-made) at a final concentration (25 pg/μL final concentration). The mixture was then incubated at 37°C for 1 h. RNA products were collected using the LiCl precipitation technique. The RNA pellet was dissolved in water and subjected to 5% dPAGE, and The RNA bands were visualized by handy UV ramp. The circular RNA bands were cut and the gel pieces were immersed in water (1.0 mL). The gel pieces were thoroughly clashed and shaken at room temperature overnight to extract the circular mRNA. 3 M Sodium acetate aq. (pH 5.2, 125 μL), isopropanol (1.25 mL), and 20 mg/mL glycogen aqueous solution (12.5 μL), were added to the extracted RNA solution to precipitate RNA (1.00 mL). The mixture was cooled to –80 ºC for 30 minutes and centrifuged (15,000 rpm, 4 ºC, 15 minutes). The supernatant was removed, and 80% aqueous ethanol (1.0 mL) was added to the pellet, and centrifuged (15,000 rpm, 4 °C, 15 min). The supernatant was removed, and the pellet was dried under reduced pressure. The resulting white solid was dissolved in water and quantified by absorption at 260 nm using a NanoDrop spectrophotometer. The RNA solution was

analyzed using 5% dPAGE (26 mA, 2 h, SYBR Green II staining). Gel images were obtained using a ChemiDoc (Bio-Rad).

2-7-2. Evaluation of translation activities of circular mRNAs

Measurement of translation activity of circular mRNAs in HeLa cells: HeLa cells cultured in a dish were washed twice with 3 mL PBS. Cells were trypsinized and seeded into a 96-well cell culture plate at 10 × 10E4 cells per well. Thereafter, the cells were incubated for 24 h at 37°C in a 5% CO_2 atmosphere. The medium was removed and the cells were washed once with 150 µL of PBS. Lipofectamine® MessengerMAX transfection reagent (Invitrogen, 0.15 µL/well) and Opti-MEM (Thermo Fisher Scientific, 10 µL/well) were mixed, and 32.48 µL of each solution was added to 32 ng of mRNA. After adding 89 µL Opti-MEM to each well, 11.3 µL of the prepared mRNA/Lipofectamine mixture was added to each well. After incubating for 3 h at 37°C in a 5% CO_2 atmosphere, the supernatant was removed, and the medium was replaced with DMEM containing 10% feral bovine serum. 24 hours after transfection, the medium was removed from the 96-well plate and washed once with 150 µL PBS. Luminescence measurements were performed using a NanoGlo® Luciferase Assay System (Promega). The measurements were performed using a plate reader (Berthold).

Measurement of translation activity of circular mRNAs in Mice: LNP-formulated circular mRNAs were injected into ICR45w-female mice (0.25 or 0.05 mg/kg) from the tail vein. 24 hours after the injection, the liver and the spleen were collected. Tissue lysate was prepared as in the previous section for "HiBiT-expression in mice". NanoLuc expression was measured using NanoGlo® Luciferase Assay System (Promega). Briefly, a 20 µL of the lysate was mixed with 20 µL of the substrate solution, and the luminescence was measured using a luminometer (GloMax, Promega). The total protein amount in the lysate was quantified by BCA assay. NLuc expressions were expressed as RLU per mg of protein.

Statistical analysis: Pair-wise comparisons between treatments were made using a two-tailed Student t-test. For comparisons among three or more groups, the one-way analysis of variance (ANOVA), followed by Bonferroni or Tukey-Kramer test, was used. A P-value of <0.05 was considered to be significant (*; $P<0.05$, **; $P<0.001$, ****; $P<0.0001$).

3. Compounds Spectral Data

^{1}H NMR Spectrum of Compound **9** (400 MHz, $CDCl_3$)

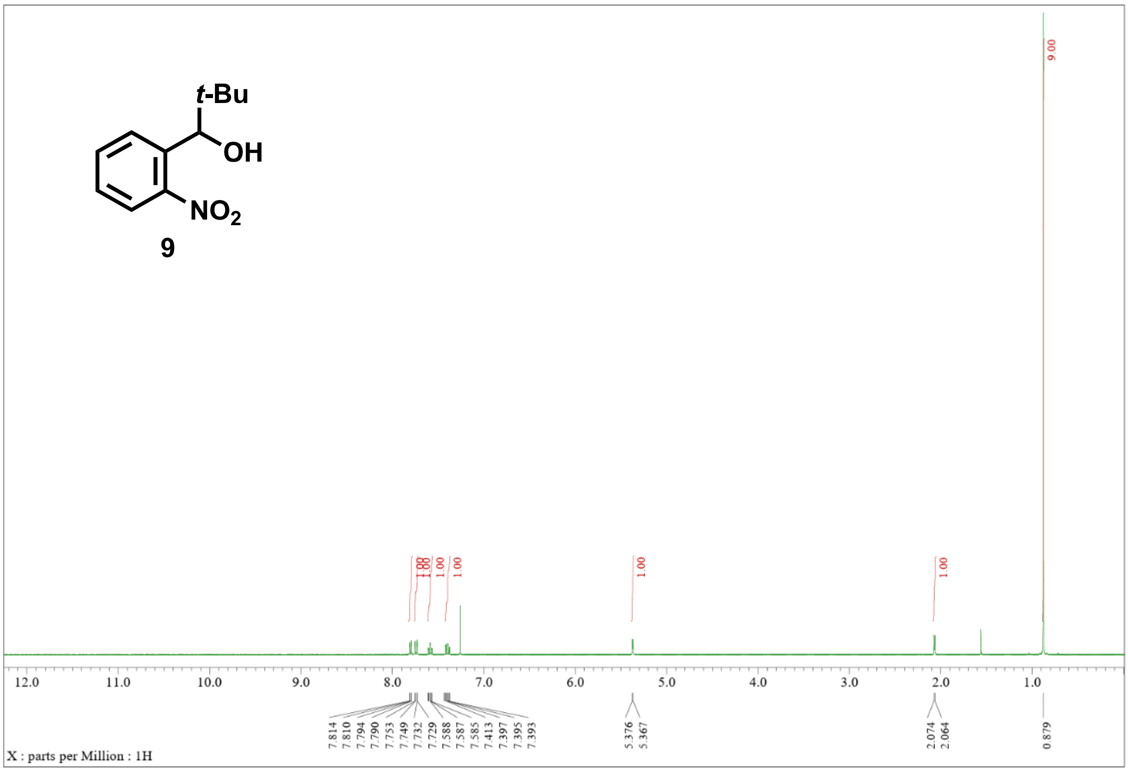

^{1}H NMR Spectrum of Compound **3** (400 MHz, $CDCl_3$)

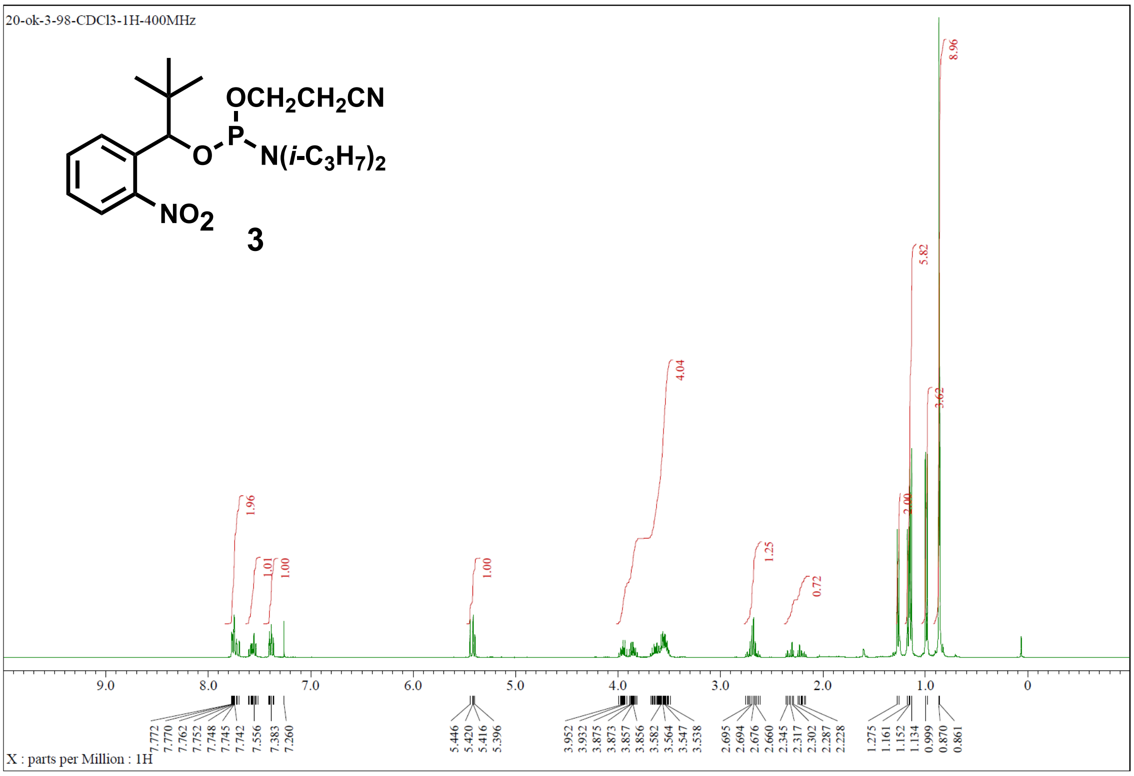

^{13}C NMR Spectrum of Compound **3** (101 MHz, $CDCl_3$)

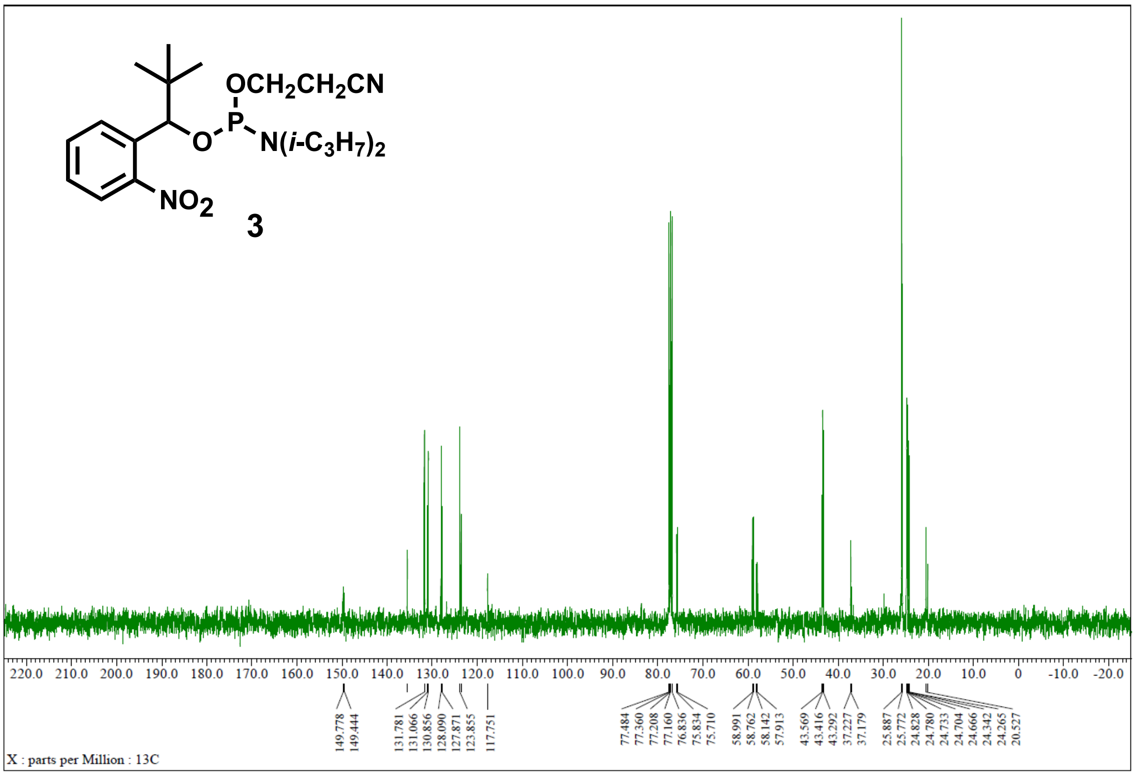

^{31}P NMR Spectrum of Compound **3** (162 MHz, $CDCl_3$)

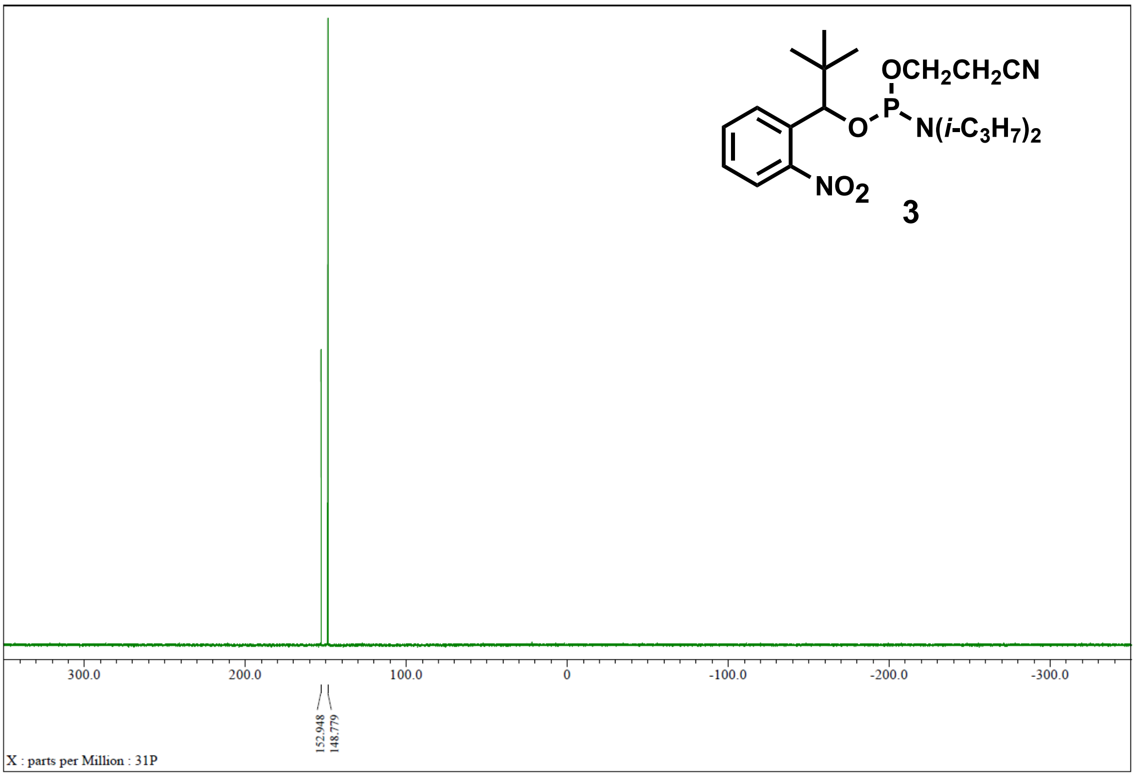

^{1}H NMR Spectrum of Compound **S2** (400 MHz, $CDCl_3$)

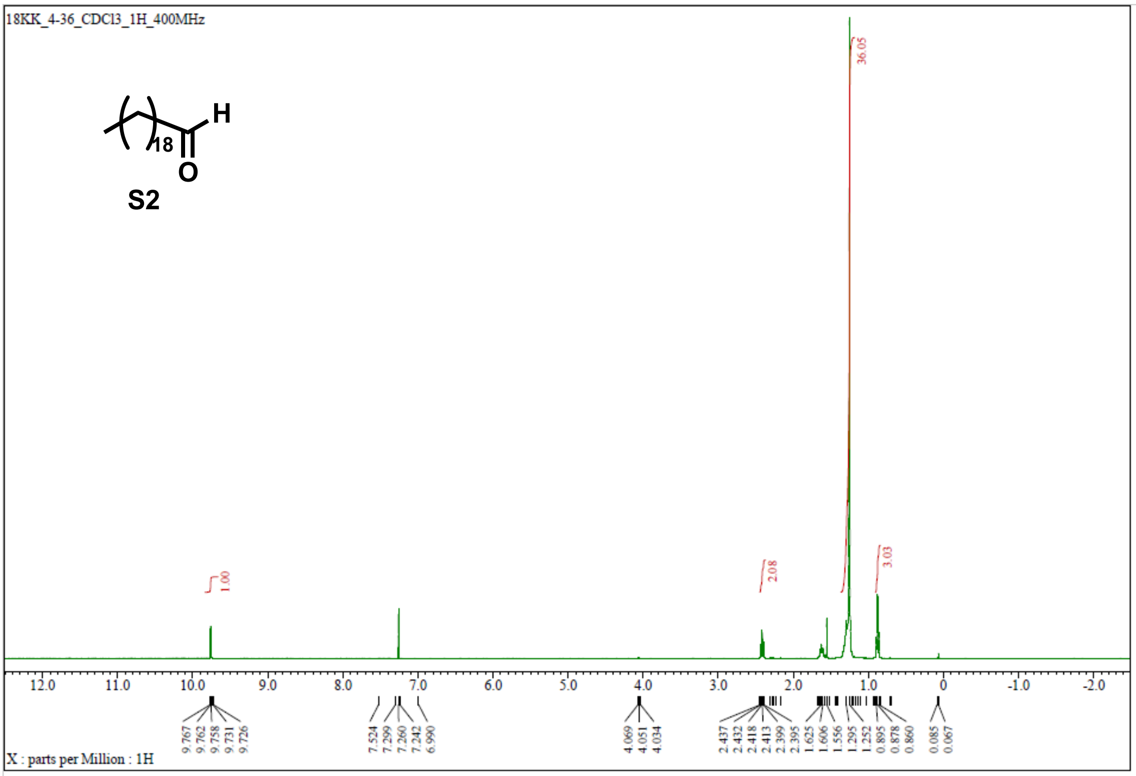

^{1}H NMR Spectrum of Compound **S3** (400 MHz, $CDCl_3$)

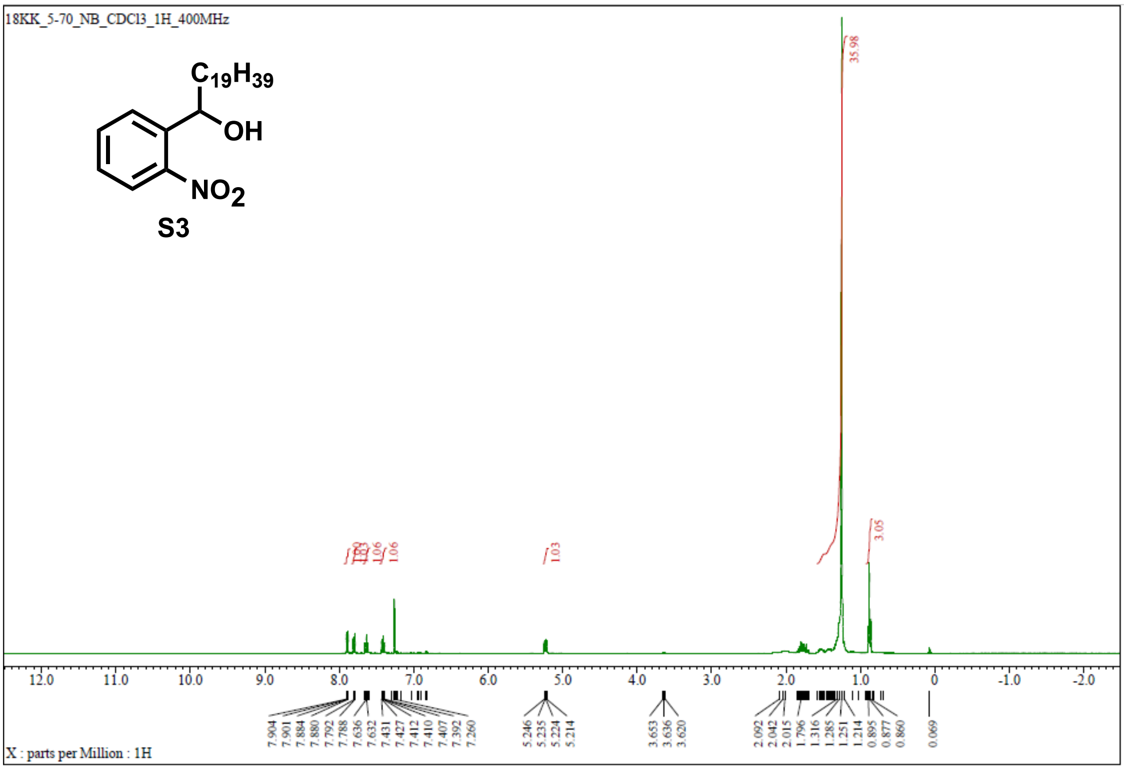

^{13}C NMR Spectrum of Compound **3** (101 MHz, $CDCl_3$)

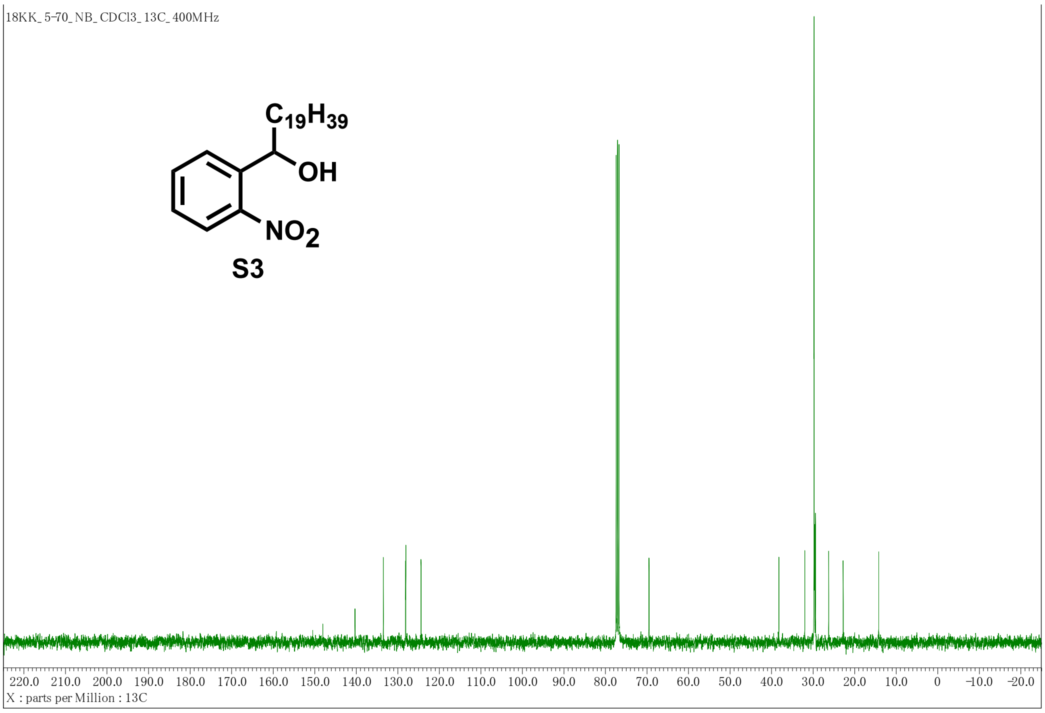

^{1}H NMR Spectrum of Compound **4** (400 MHz, $CDCl_3$)

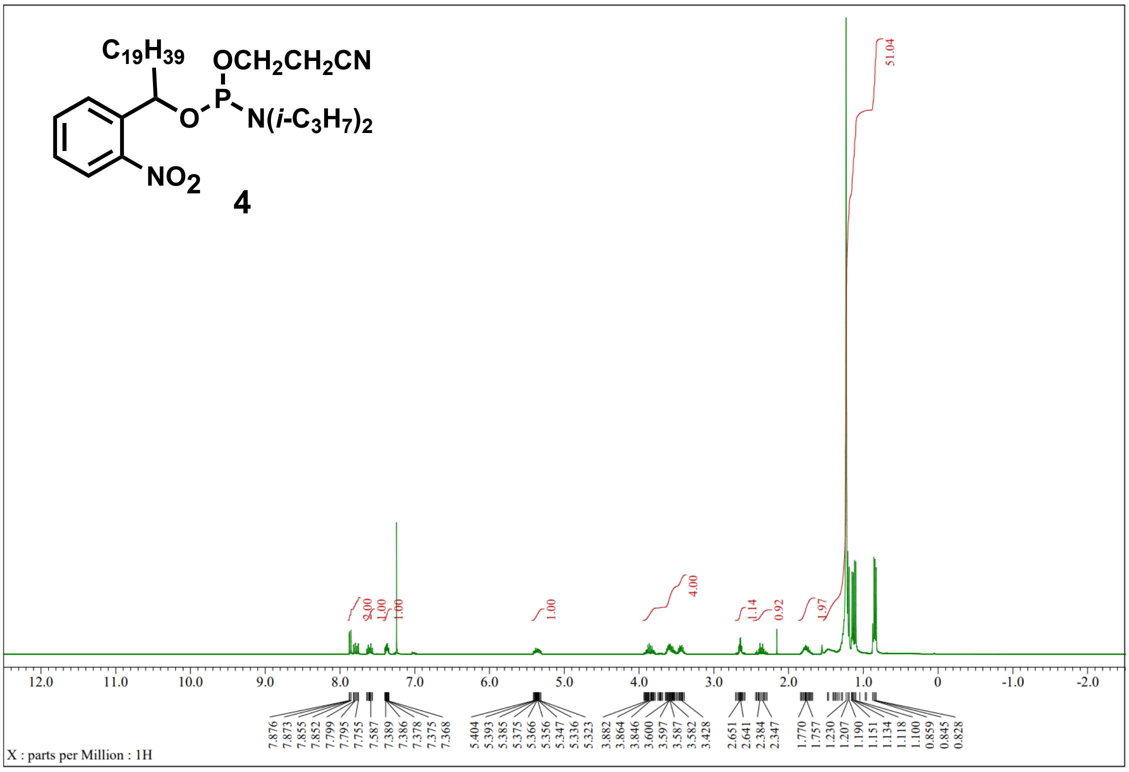

^{13}C NMR Spectrum of Compound **4** (101 MHz, $CDCl_3$)

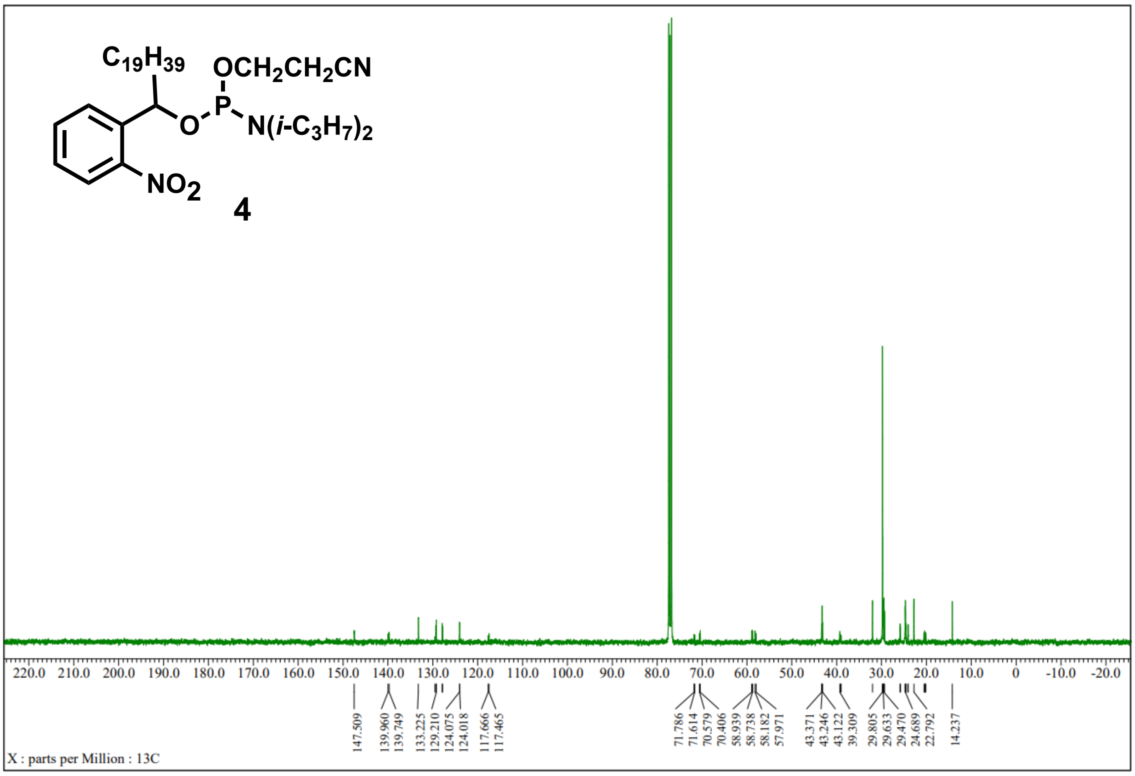

^{31}P NMR Spectrum of Compound **4** (162 MHz, $CDCl_3$)

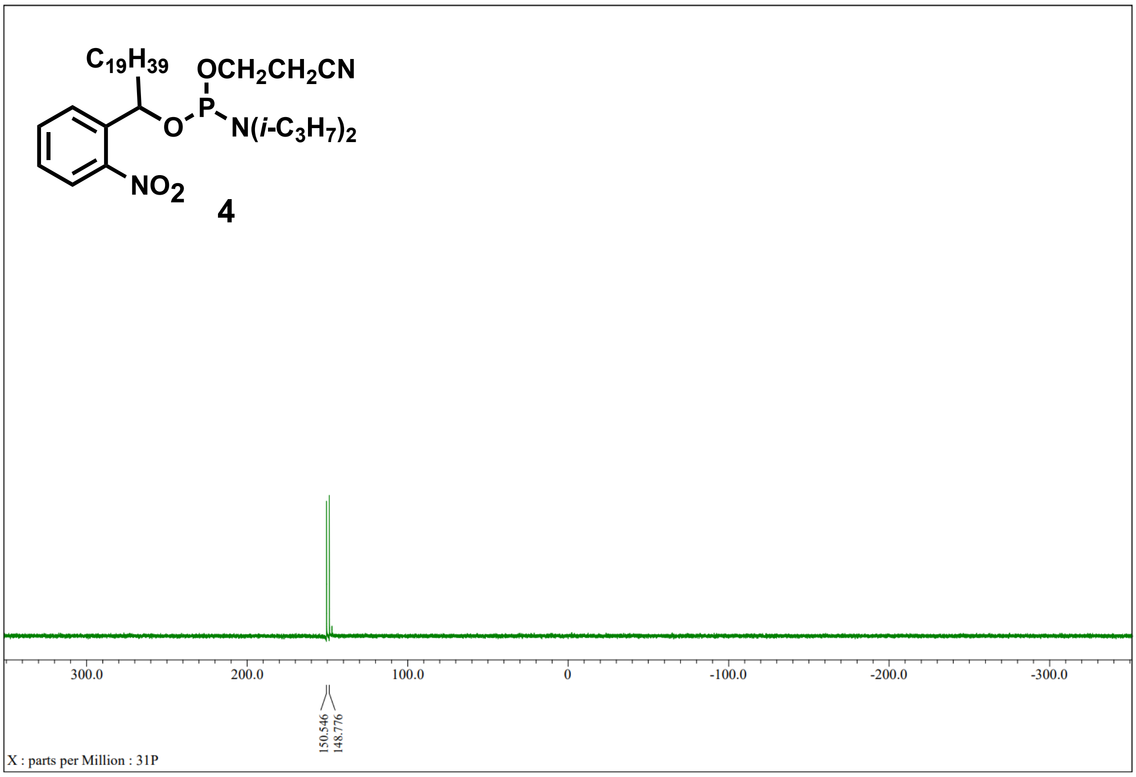

^{1}H NMR Spectrum of Compound **S5** (600 MHz, $CDCl_3$)

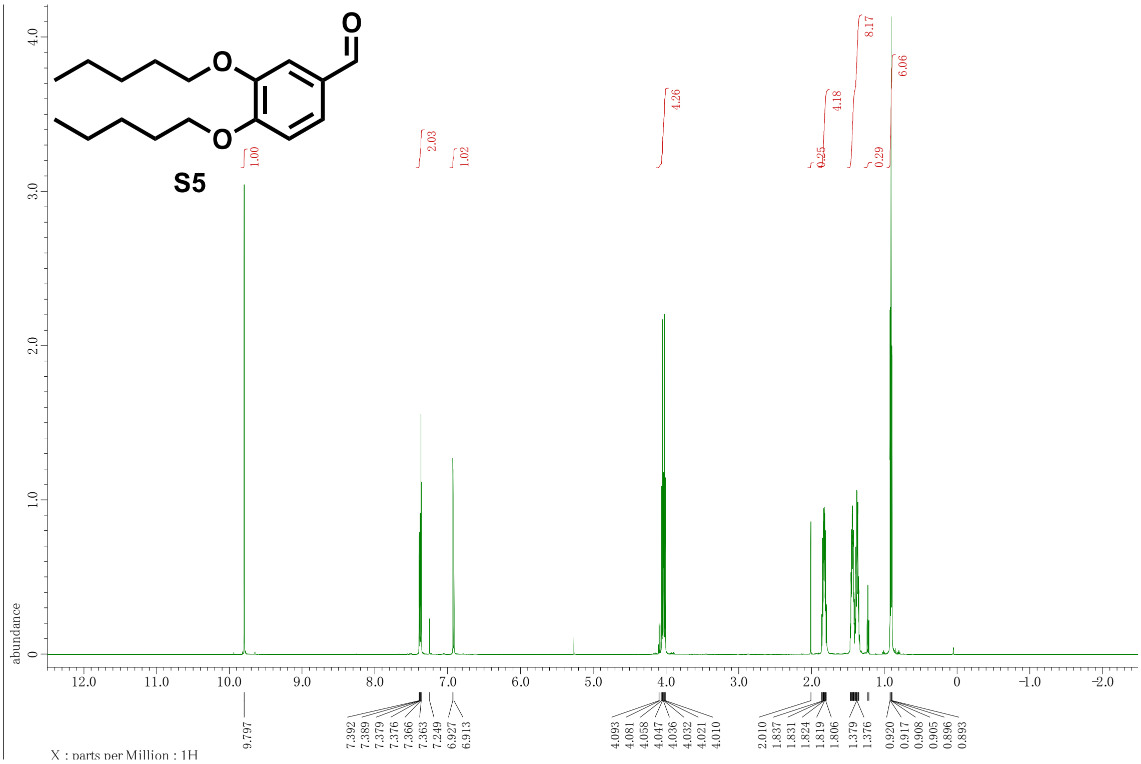

^{13}C NMR Spectrum of Compound **S5** (151 MHz, $CDCl_3$)

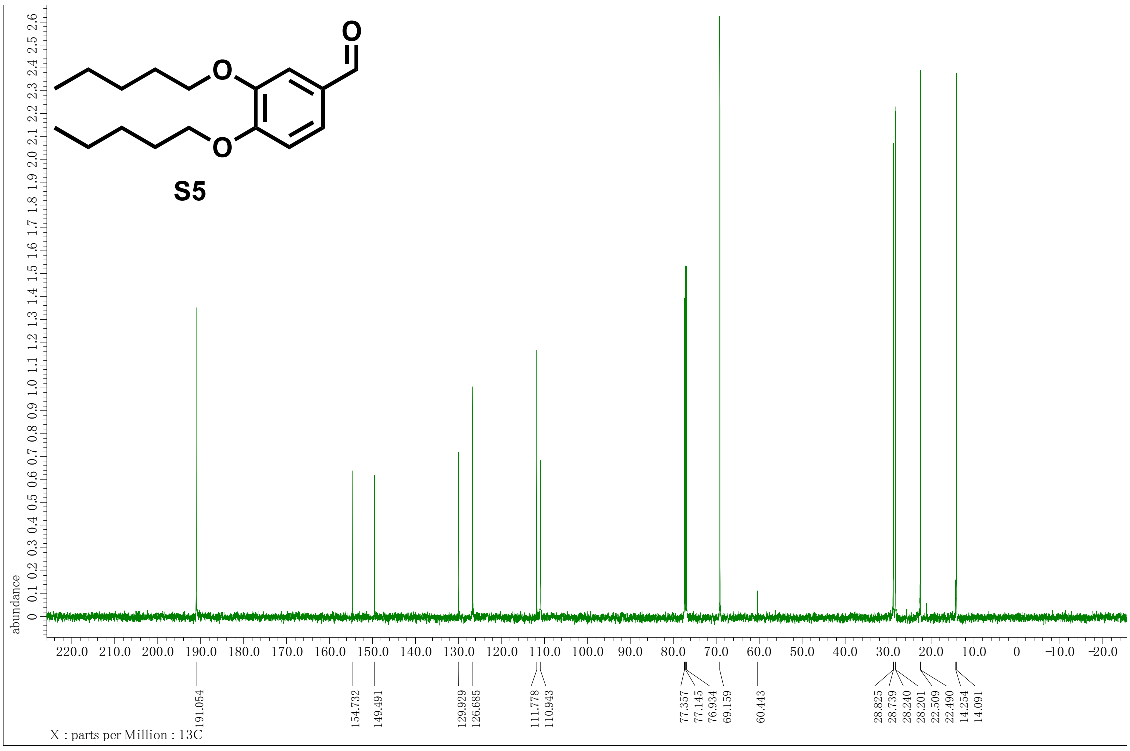

^{1}H NMR Spectrum of Compound **S6** (600 MHz, $CDCl_3$)

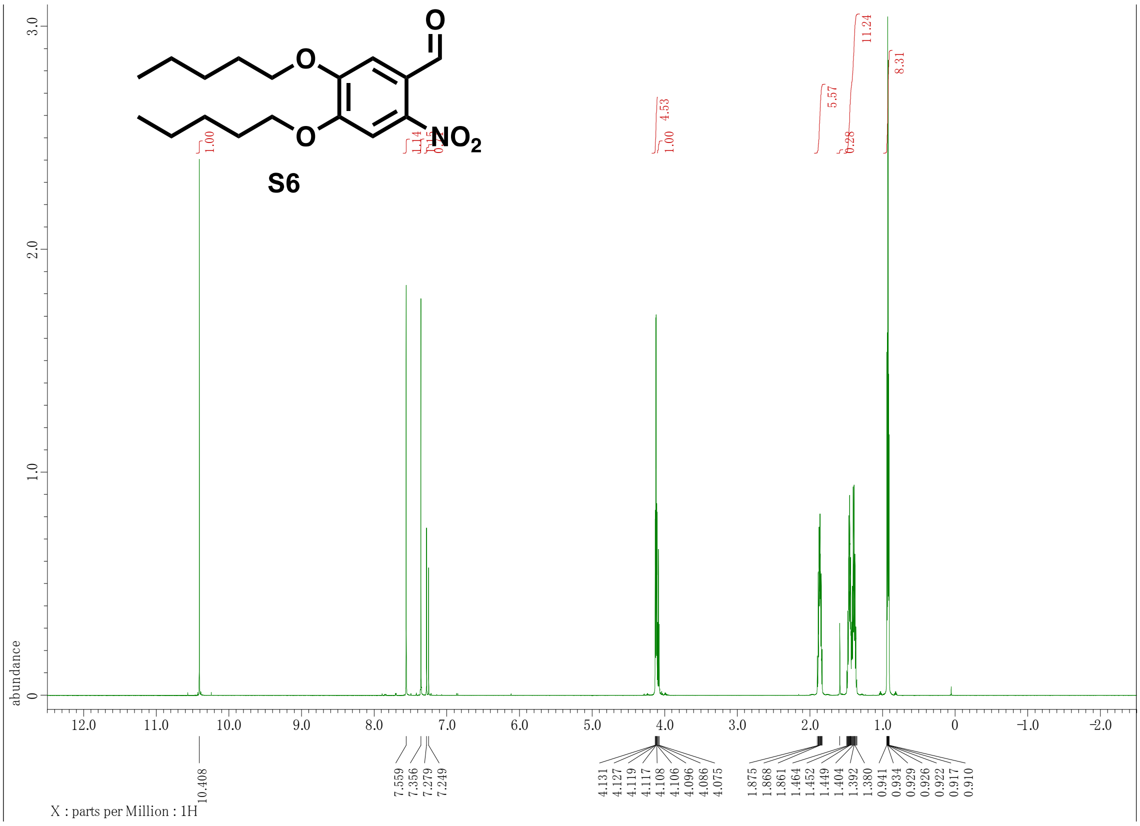

^{13}C NMR Spectrum of Compound **S6** (151 MHz, $CDCl_3$)

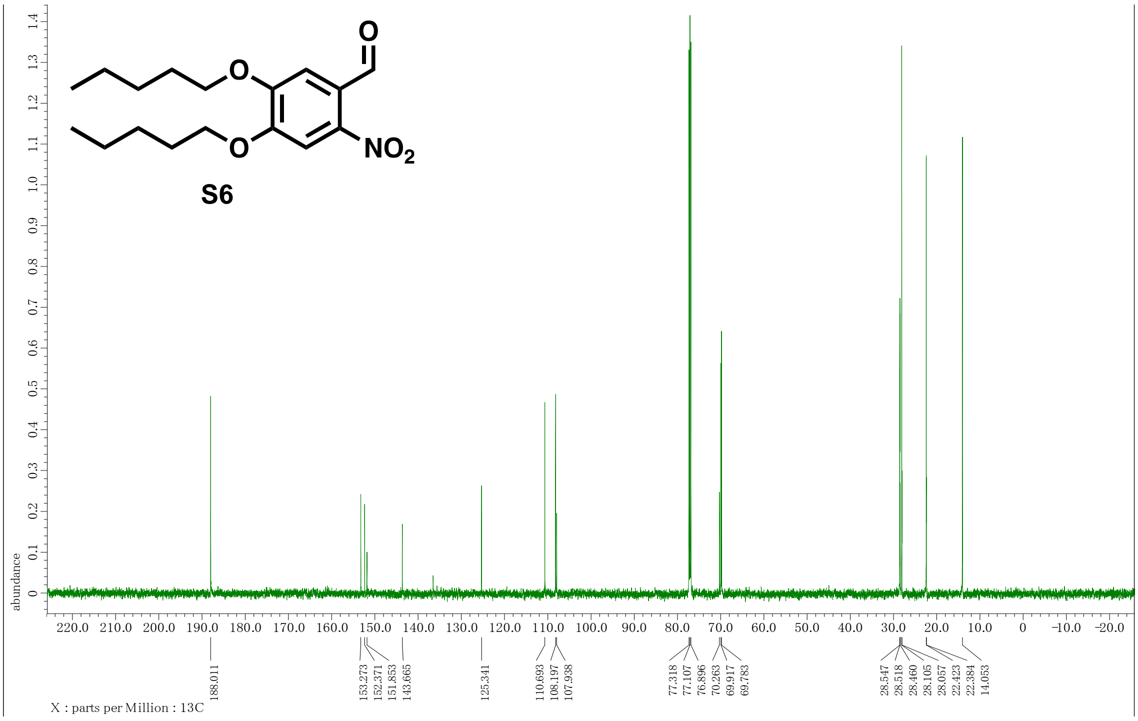

^{1}H NMR Spectrum of Compound **S7** (600 MHz, $CDCl_3$)

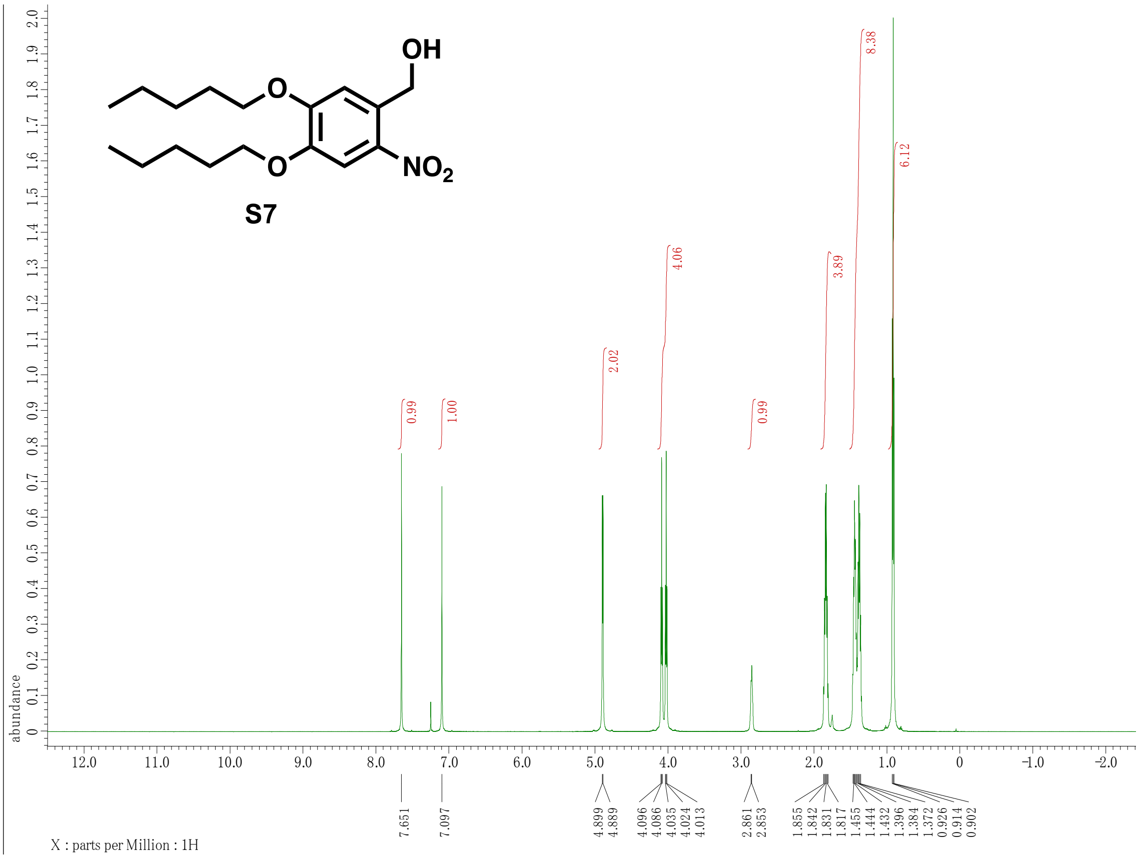

^{13}C NMR Spectrum of Compound **S7** (151 MHz, $CDCl_3$)

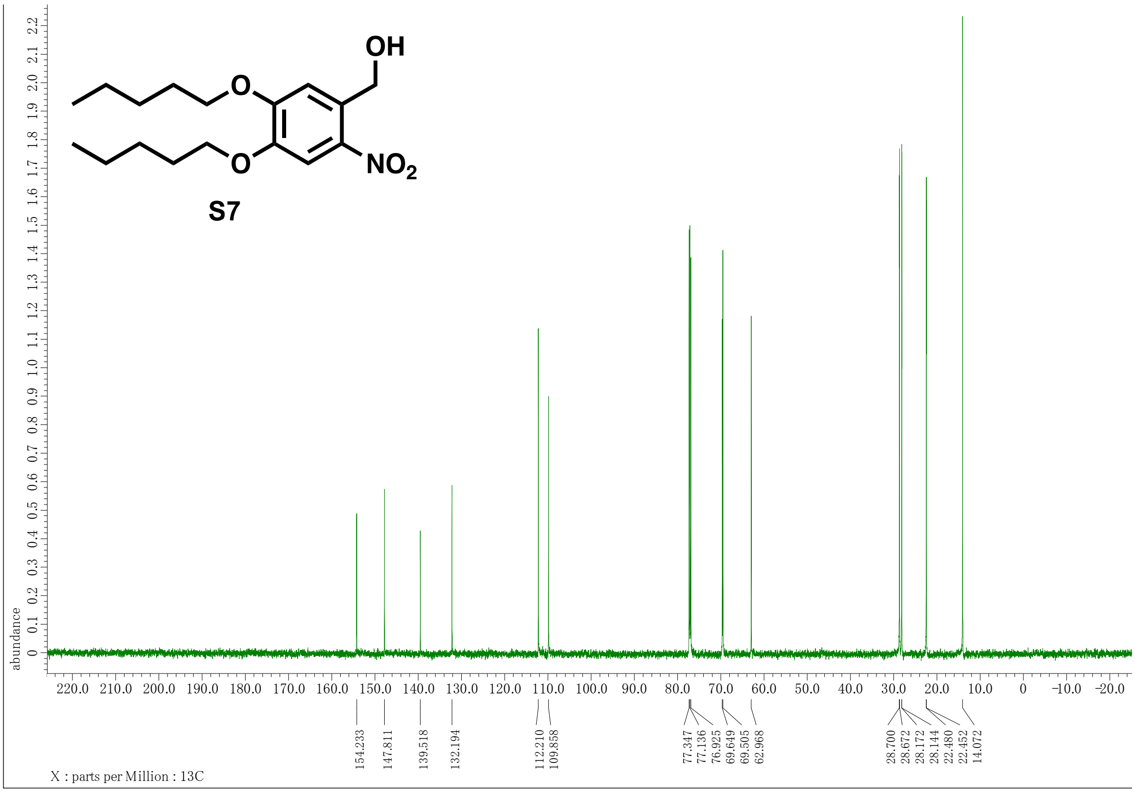

^{1}H NMR Spectrum of Compound **5** (600 MHz, CD_3CN)

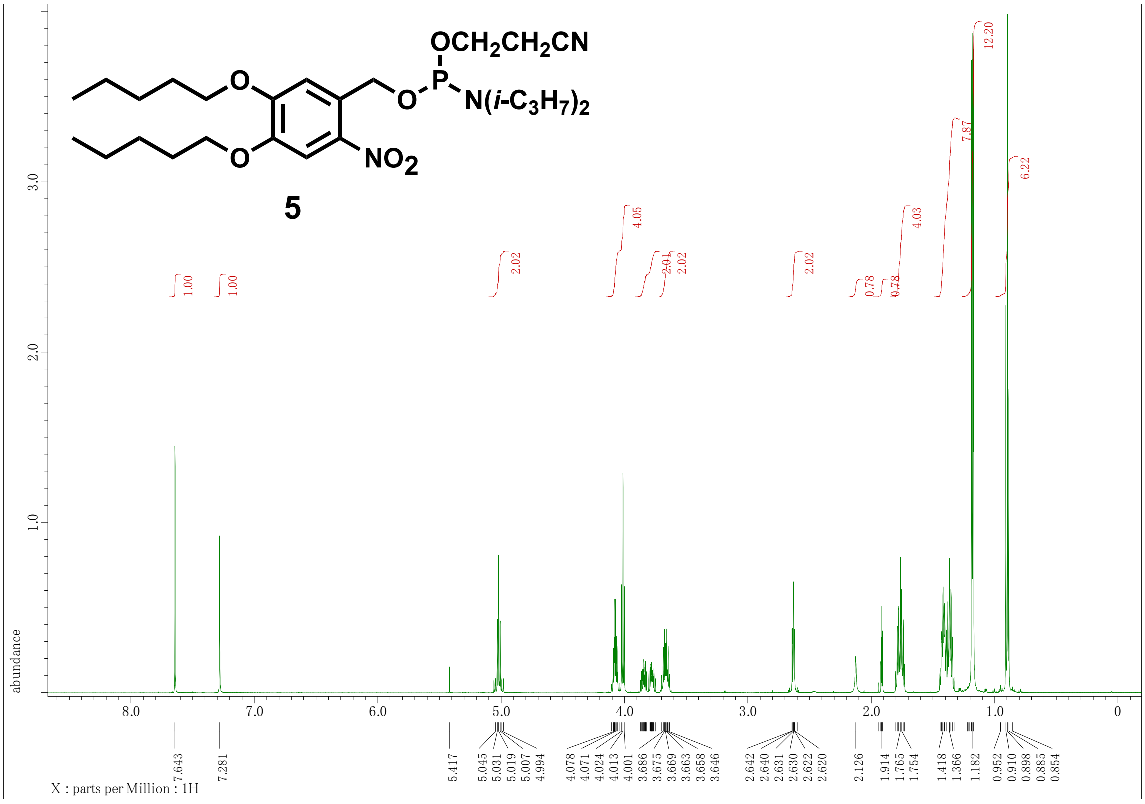

^{13}C NMR Spectrum of Compound **5** (151 MHz, CD_3CN)

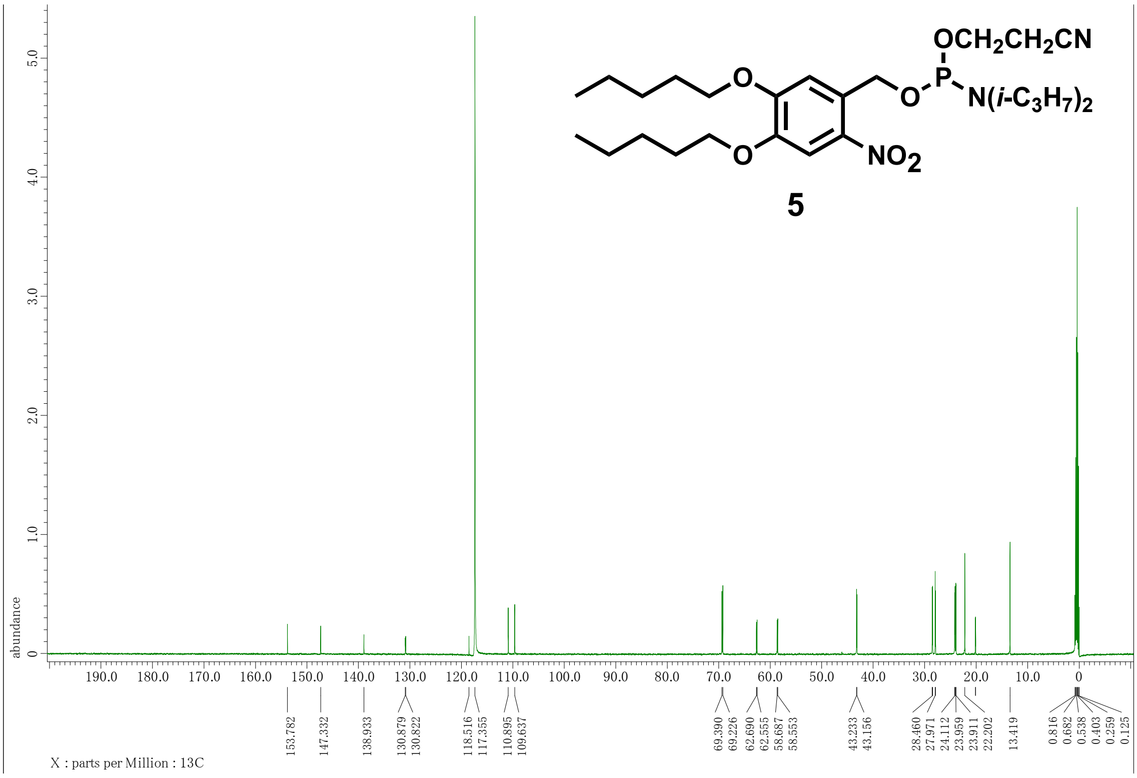

^{31}P NMR Spectrum of Compound **5** (243 MHz, CD_3CN)

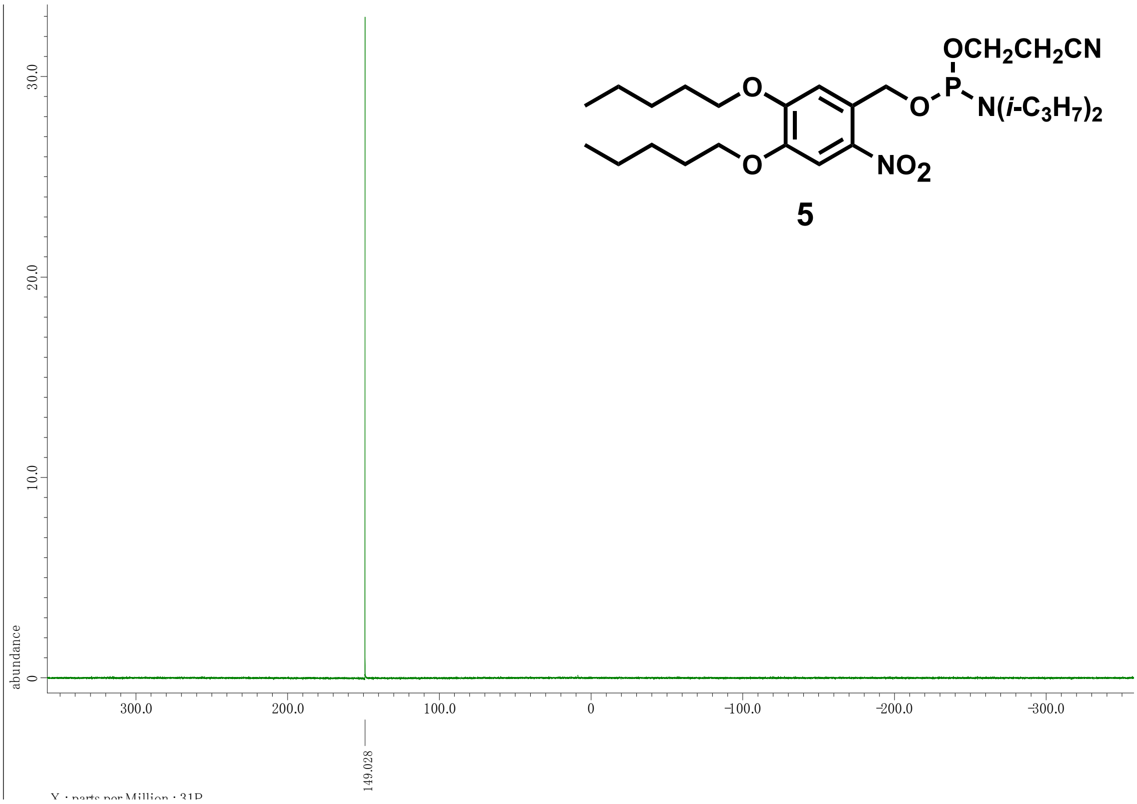

^{1}H NMR Spectrum of Compound **S8** (400 MHz, $CDCl_3$)

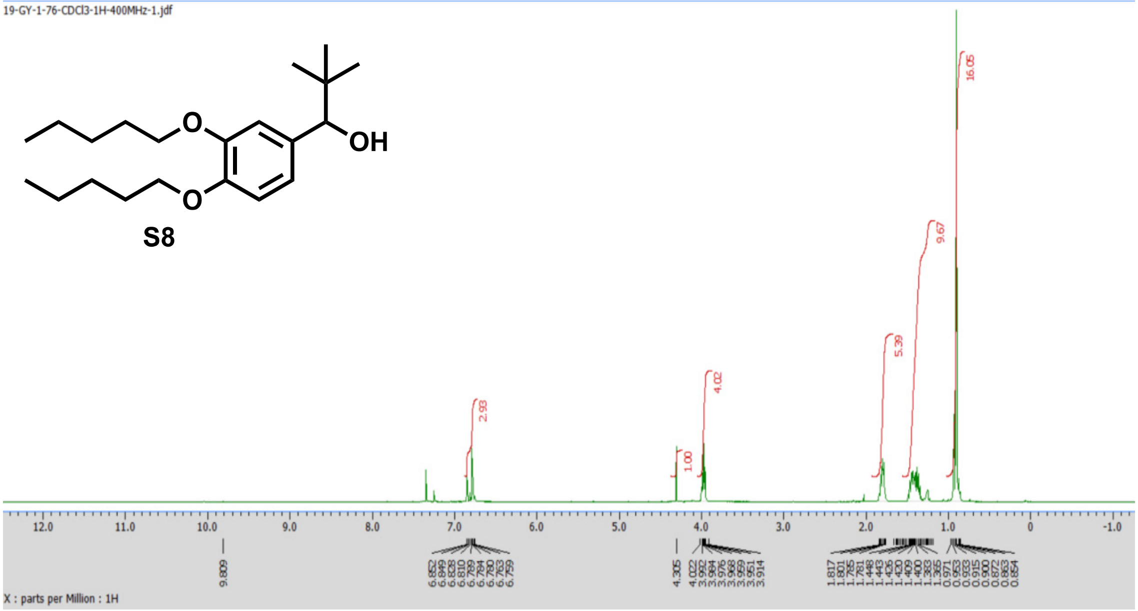

^{13}C NMR Spectrum of Compound **S8** (101 MHz, $CDCl_3$)

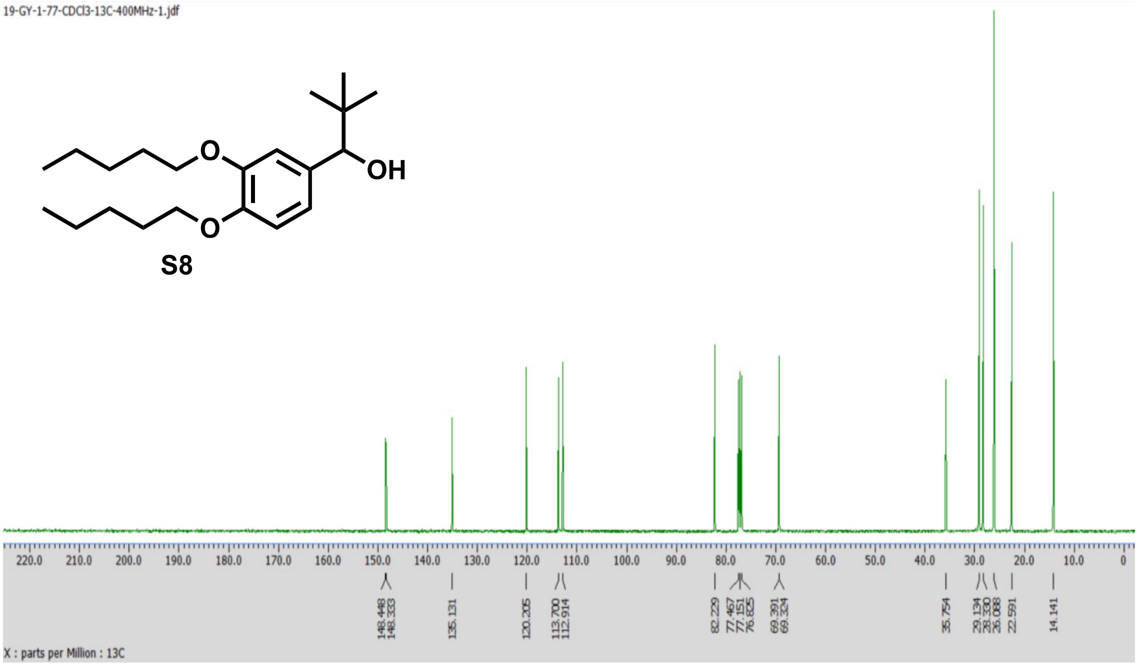

^{1}H NMR Spectrum of Compound **S9** (400 MHz, $CDCl_3$)

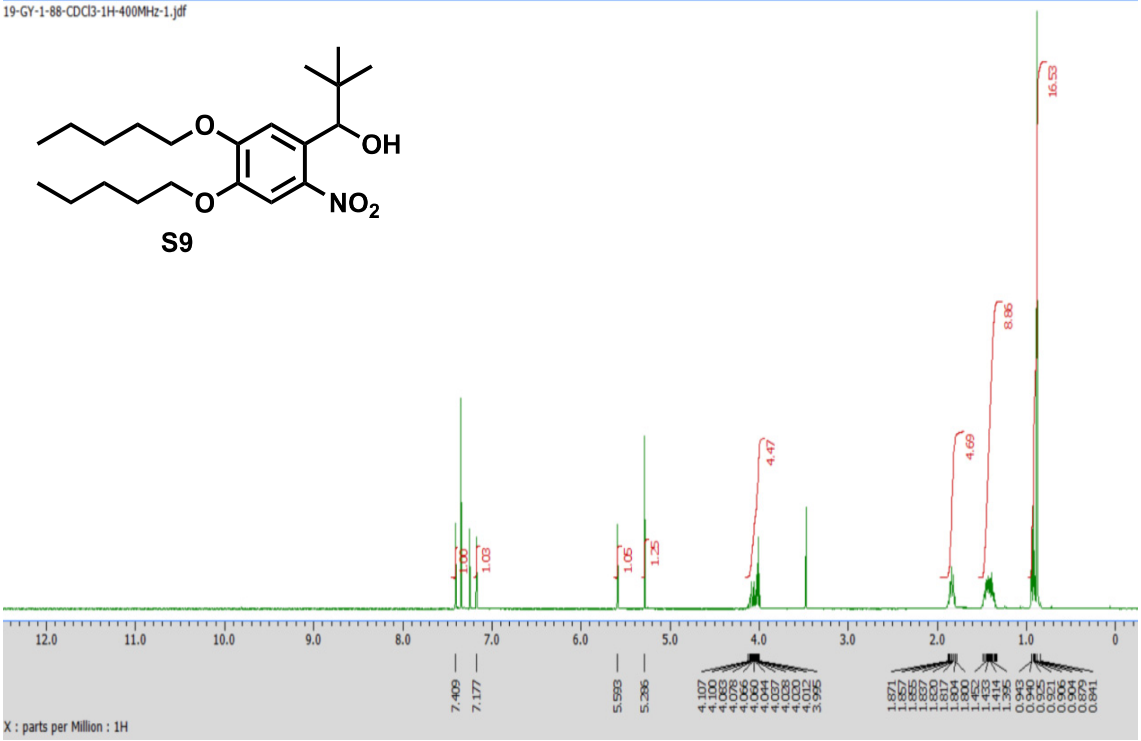

^{13}C NMR Spectrum of Compound **S9** (101 MHz, $CDCl_3$)

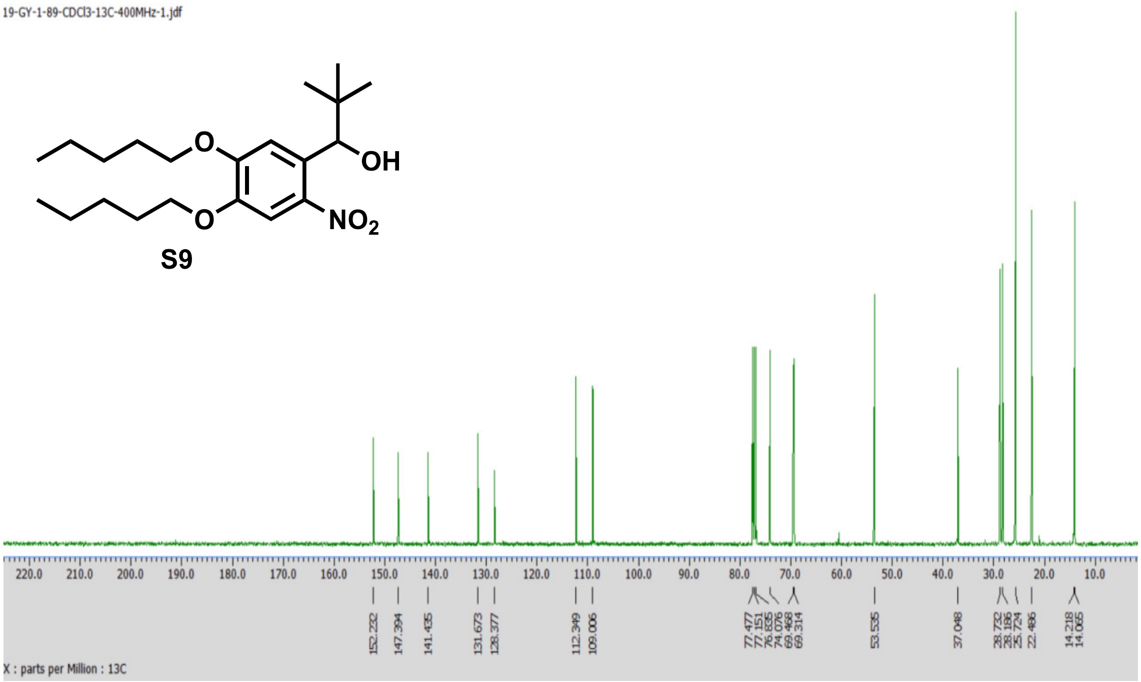

^1H NMR Spectrum of Compound **6** (400 MHz, CD_3CN)

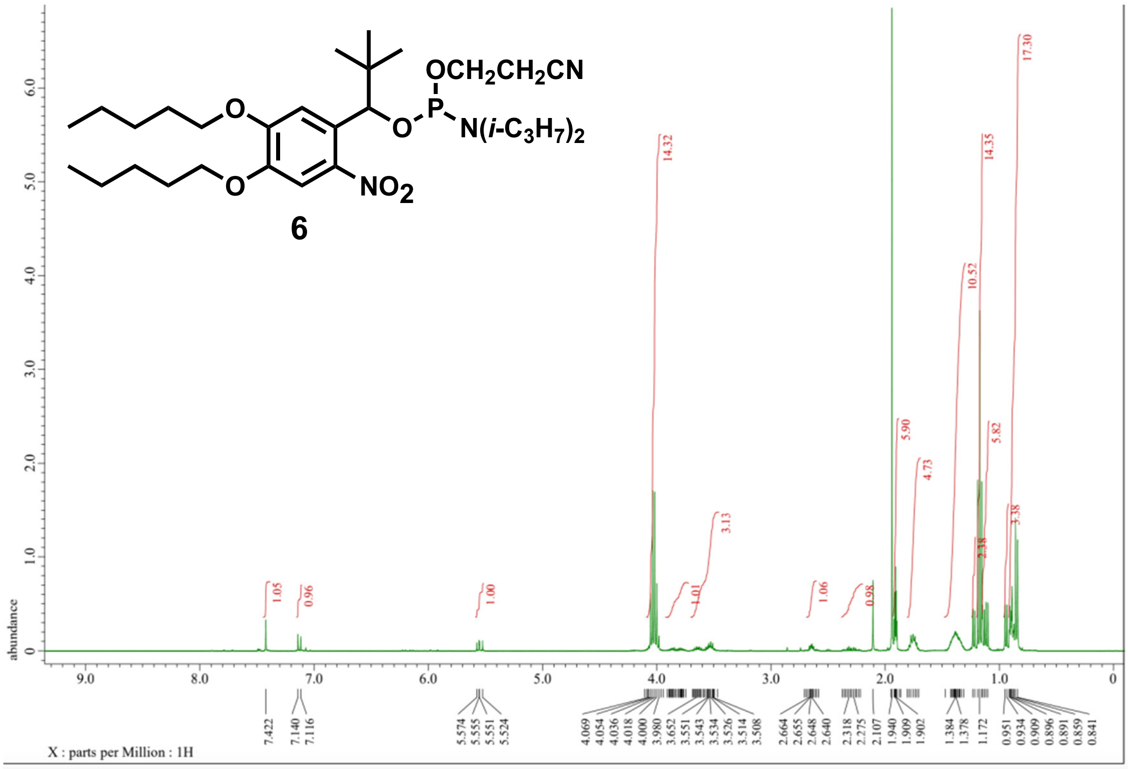

^{13}C NMR Spectrum of Compound **6** (101 MHz, CD_3CN)

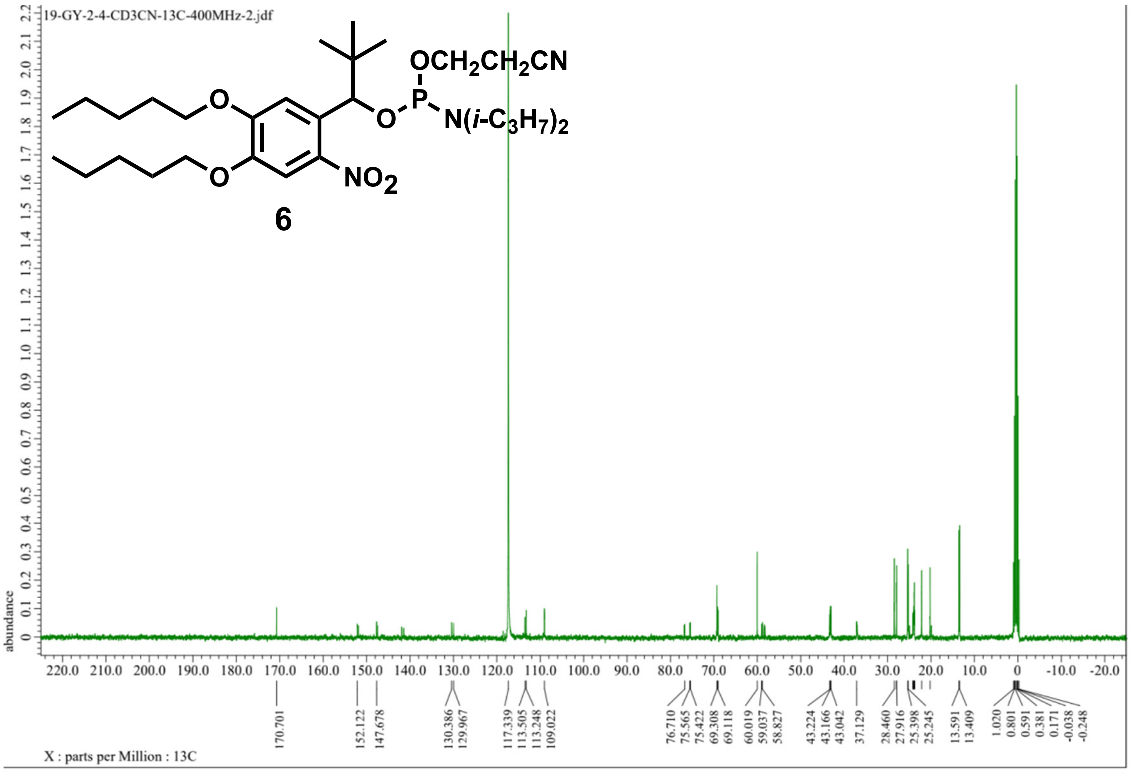

^{31}P NMR Spectrum of Compound **6** (162 MHz, CD_3CN)

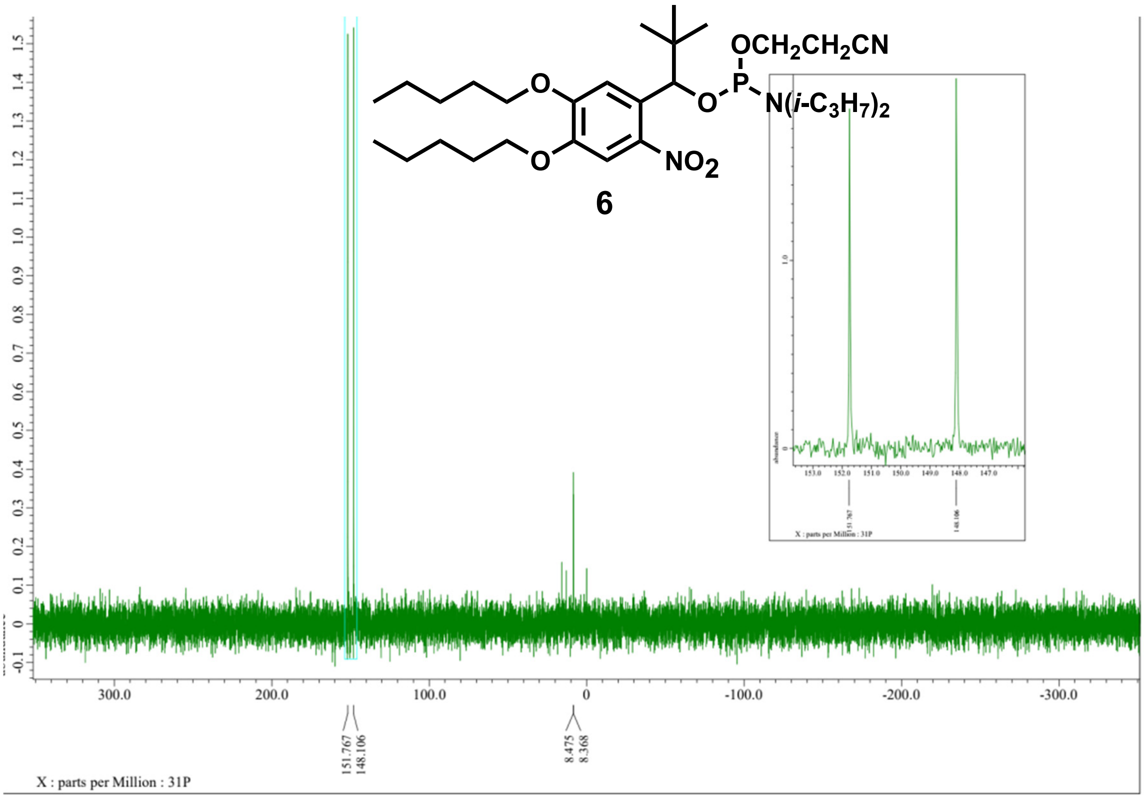

^{1}H NMR Spectrum of Compound **11** (400 MHz, $CDCl_3$)

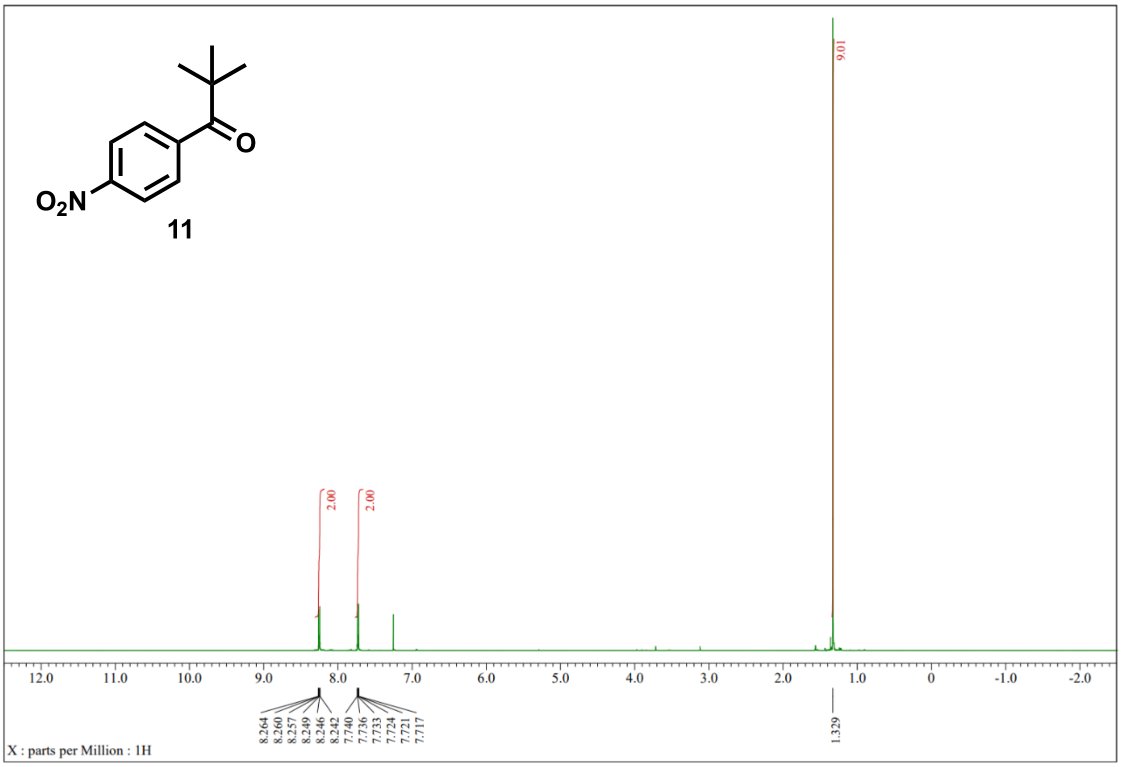

^{13}C NMR Spectrum of Compound **11** (101 MHz, $CDCl_3$)

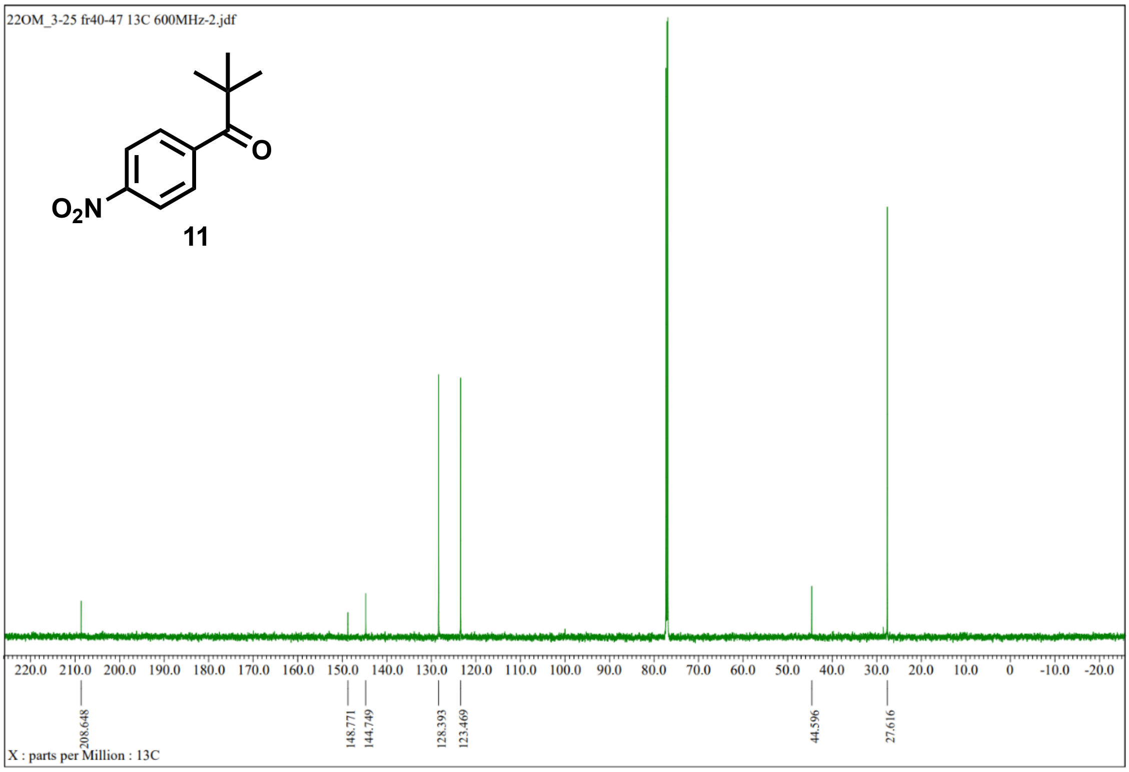

^{1}H NMR Spectrum of Compound **12** (400 MHz, $CDCl_3$)

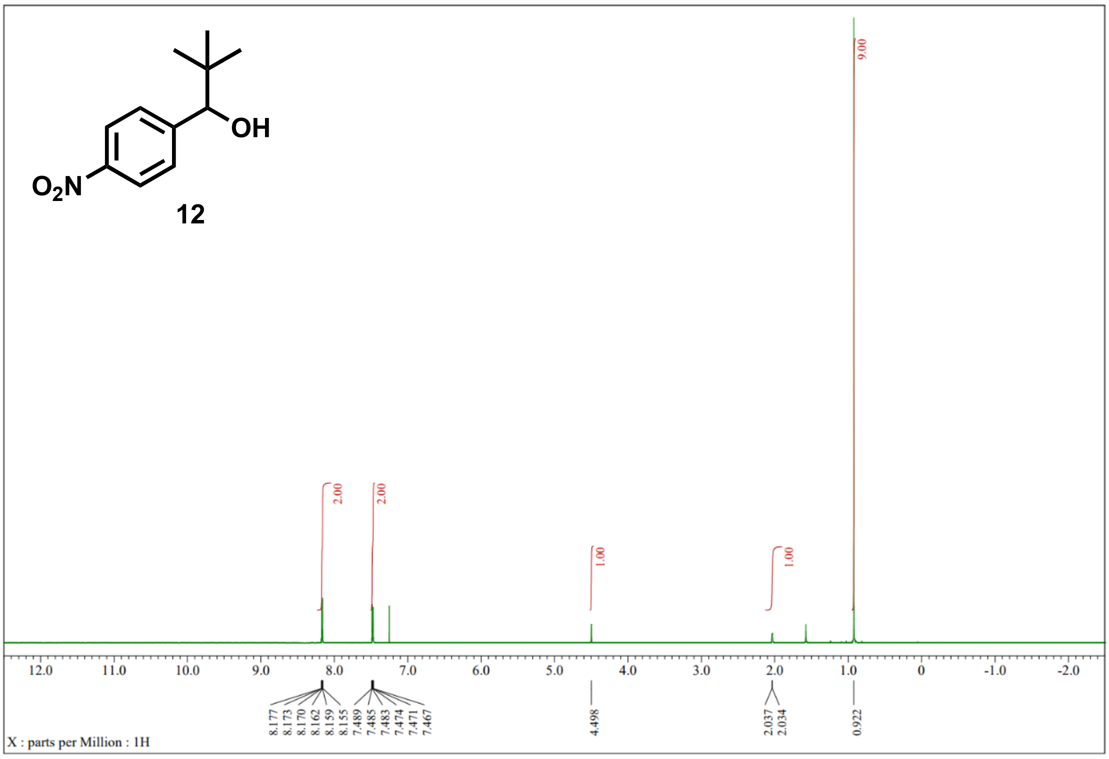

^{13}C NMR Spectrum of Compound **12** (101 MHz, $CDCl_3$)

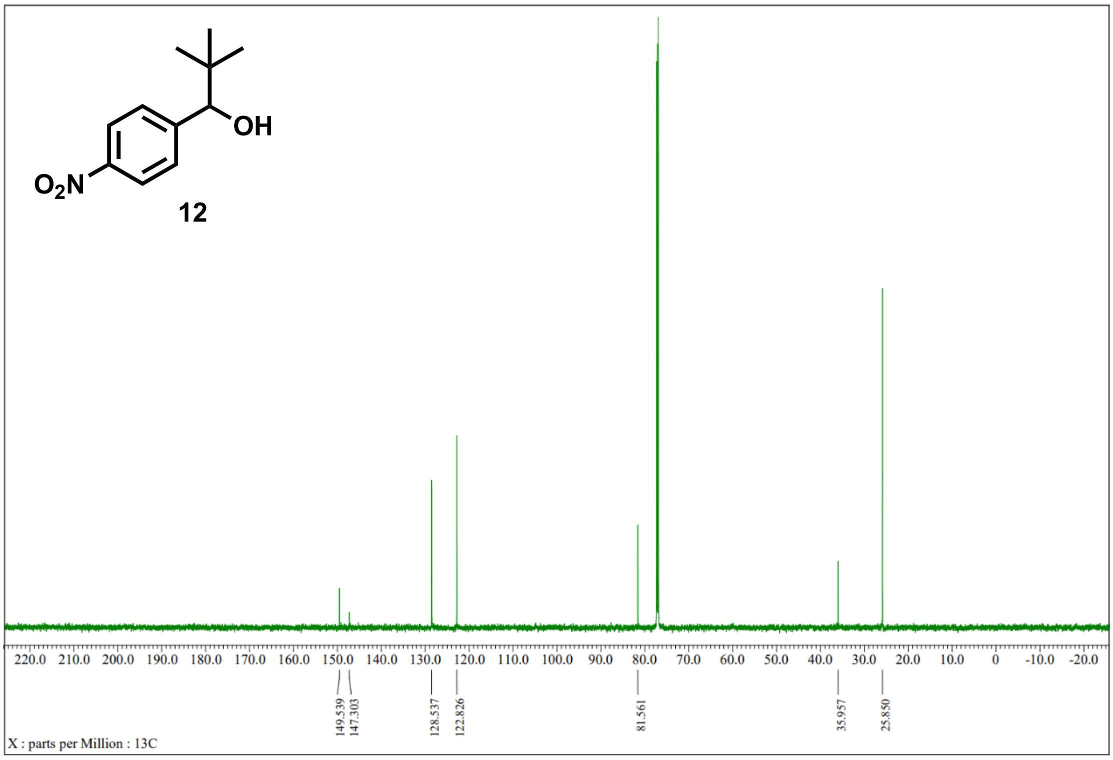

^{1}H NMR Spectrum of Compound **7** (400 MHz, $CDCl_3$)

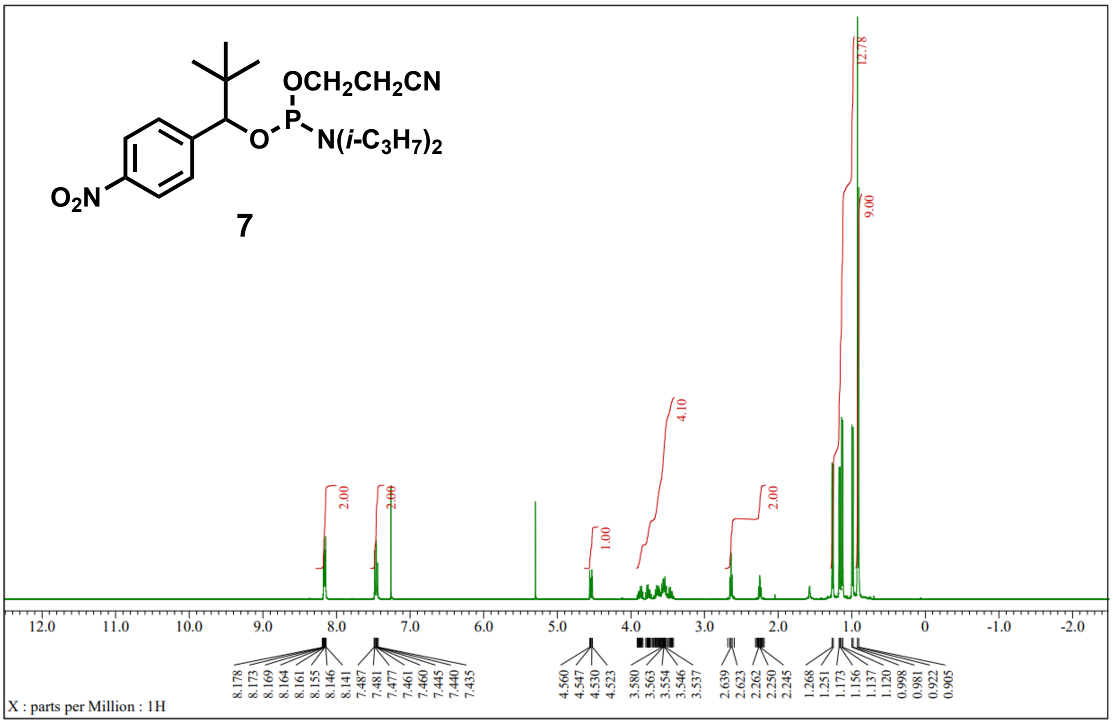

^{13}C NMR Spectrum of Compound **7** (101 MHz, $CDCl_3$)

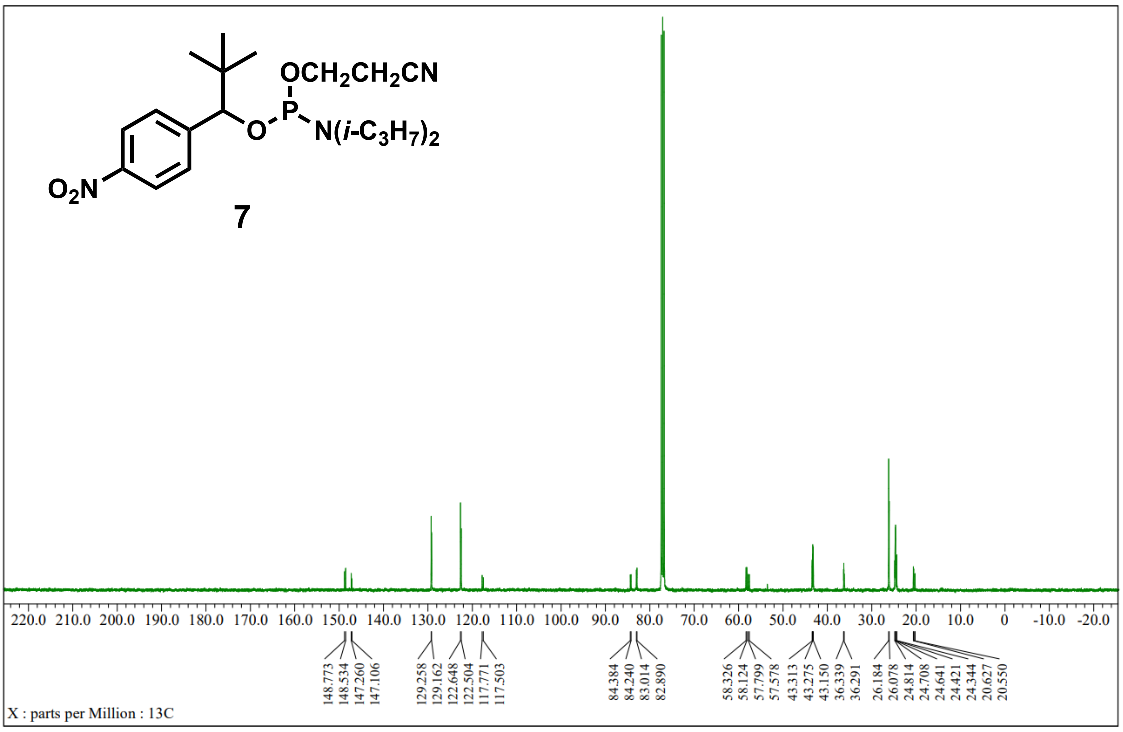

^{31}P NMR Spectrum of Compound **7** (162 MHz, $CDCl_3$)

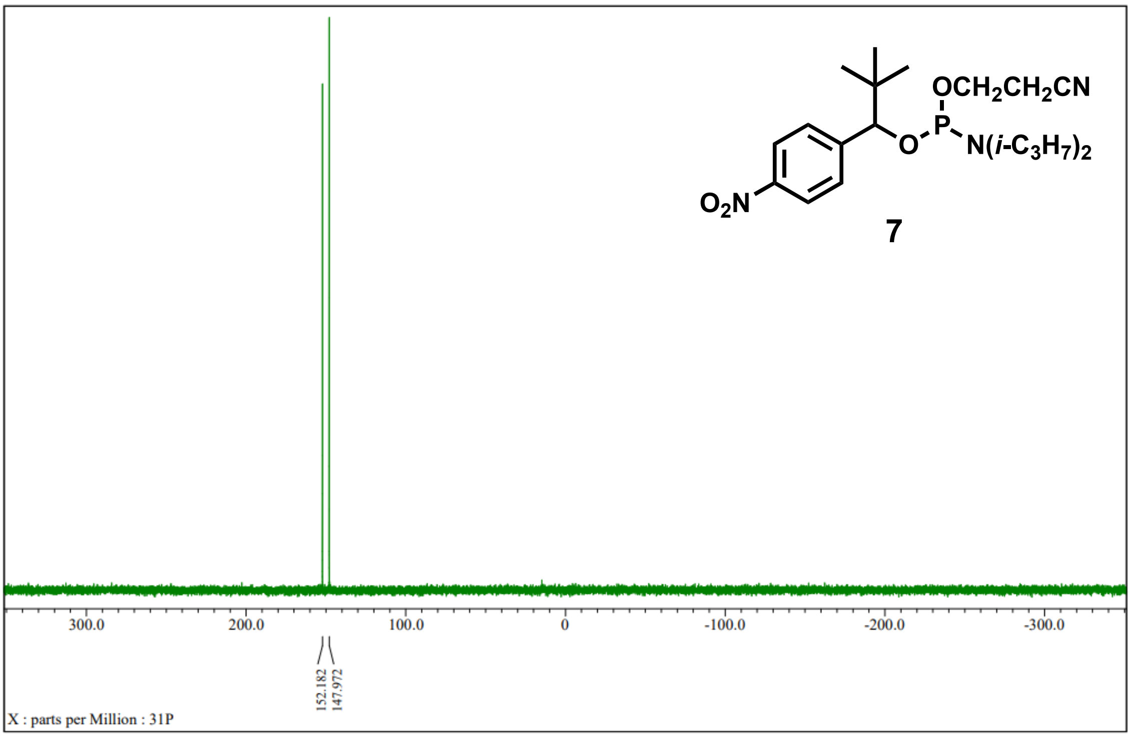

^1H NMR Spectrum of Compound **14** (600 MHz, DMSO-d_6)

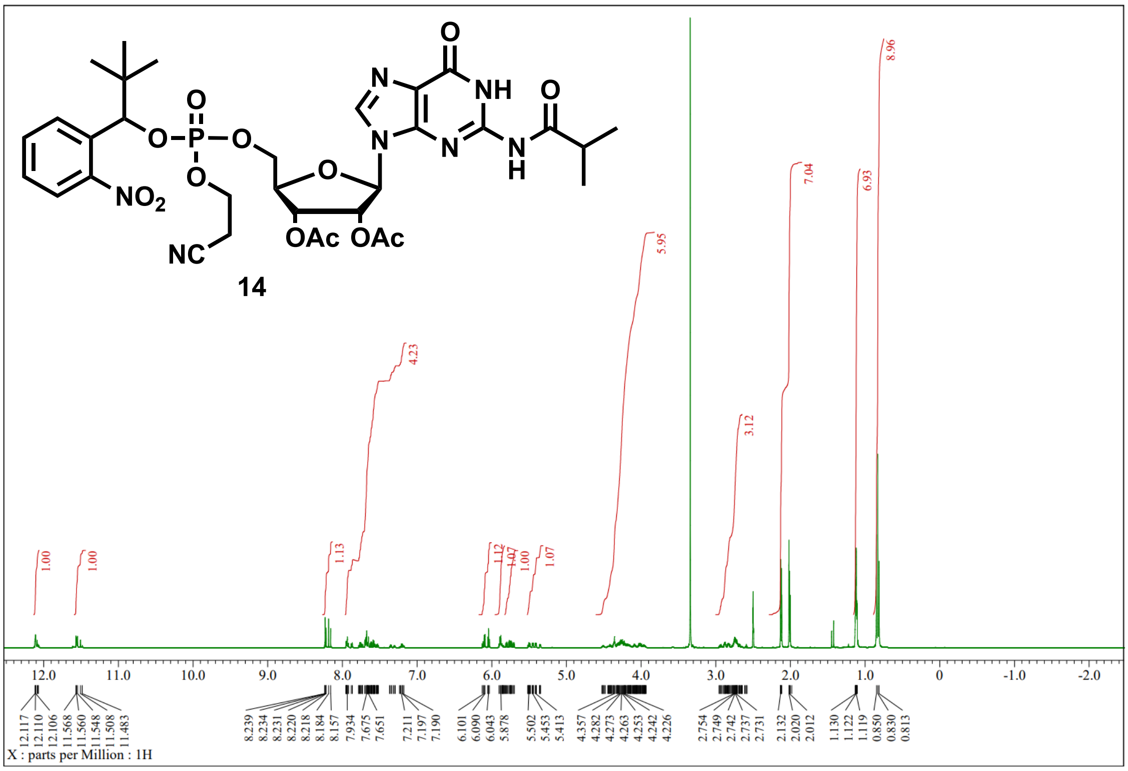

^{13}C NMR Spectrum of Compound **14** (151 MHz, DMSO-d_6)

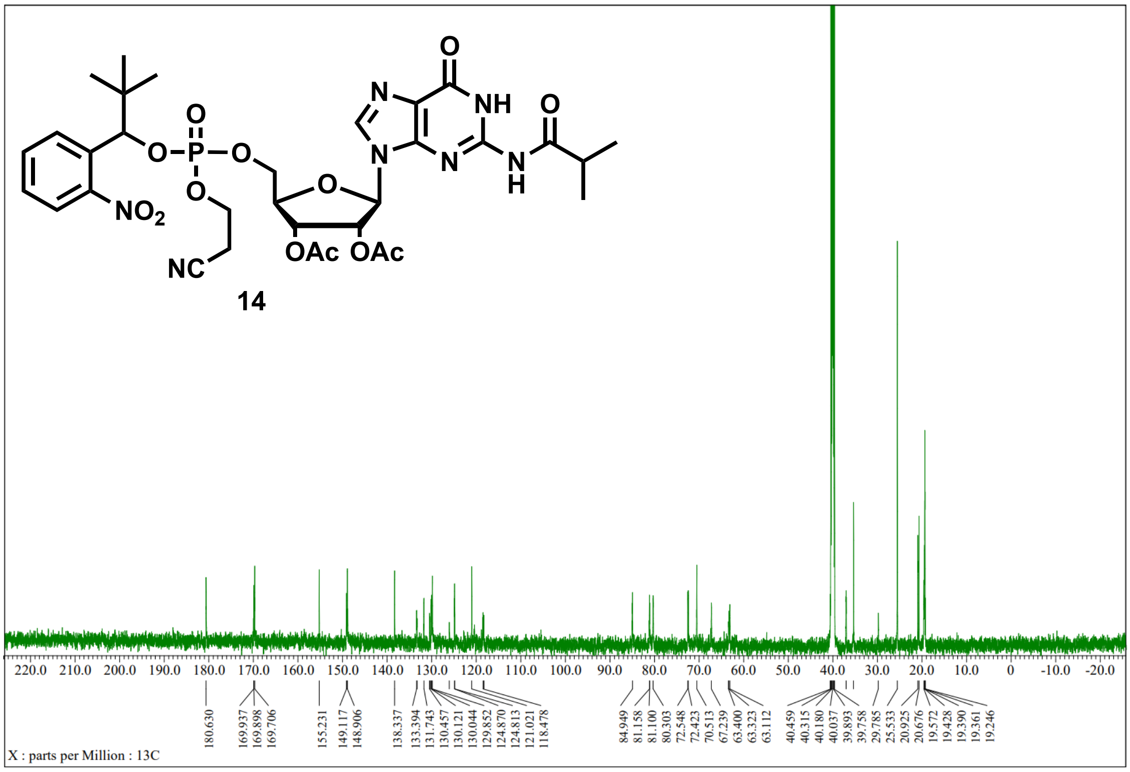

^{31}P NMR Spectrum of Compound **14** (243 MHz, DMSO-d_6)

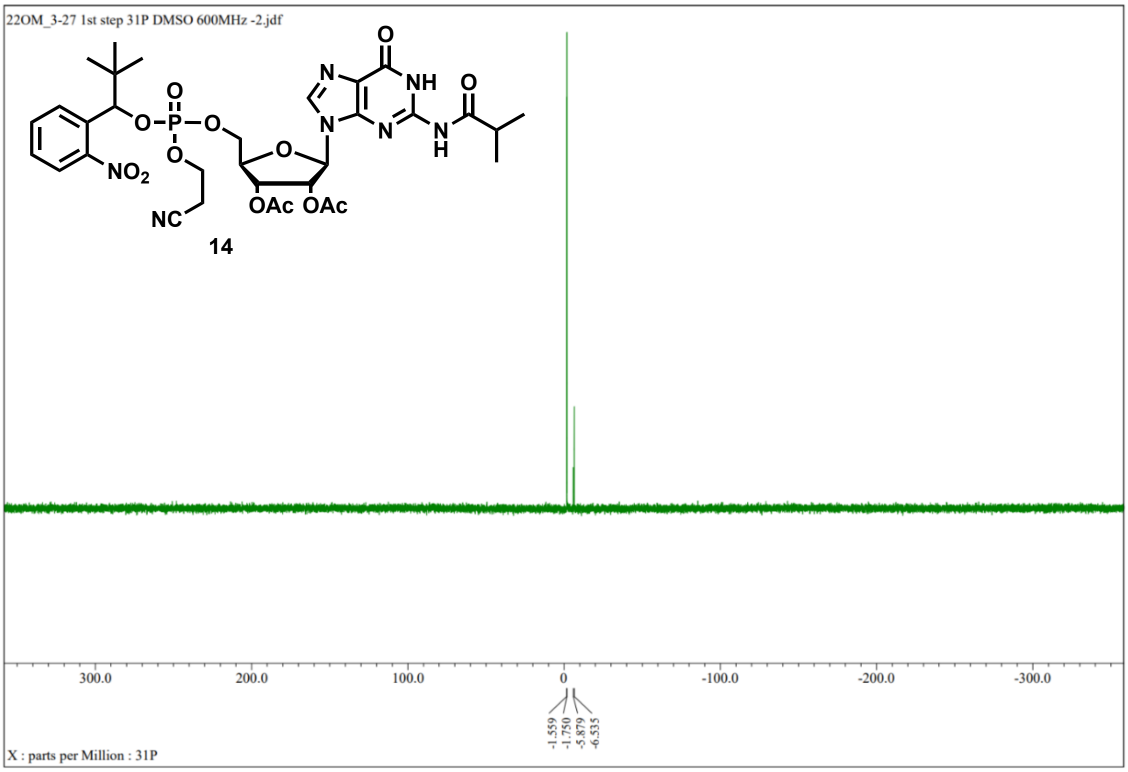

^1H NMR Spectrum of Compound **16** (600 MHz, DMSO-d_6)

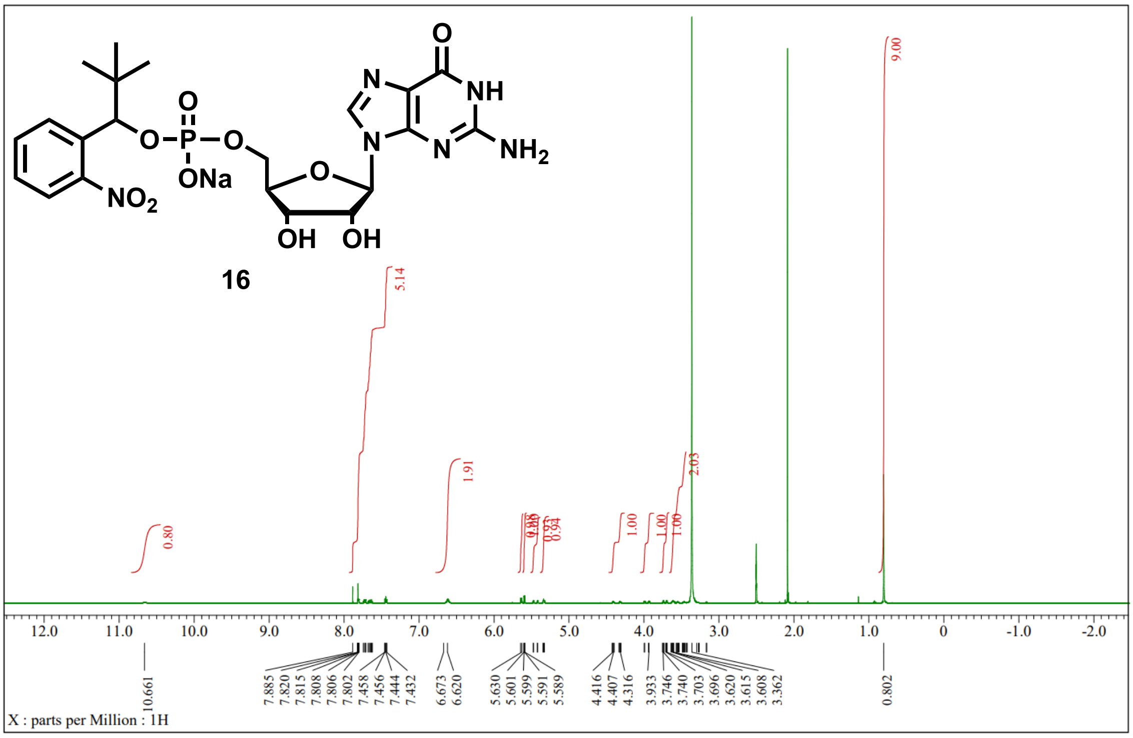

^{13}C NMR Spectrum of Compound **16** (151 MHz, DMSO-d_6)

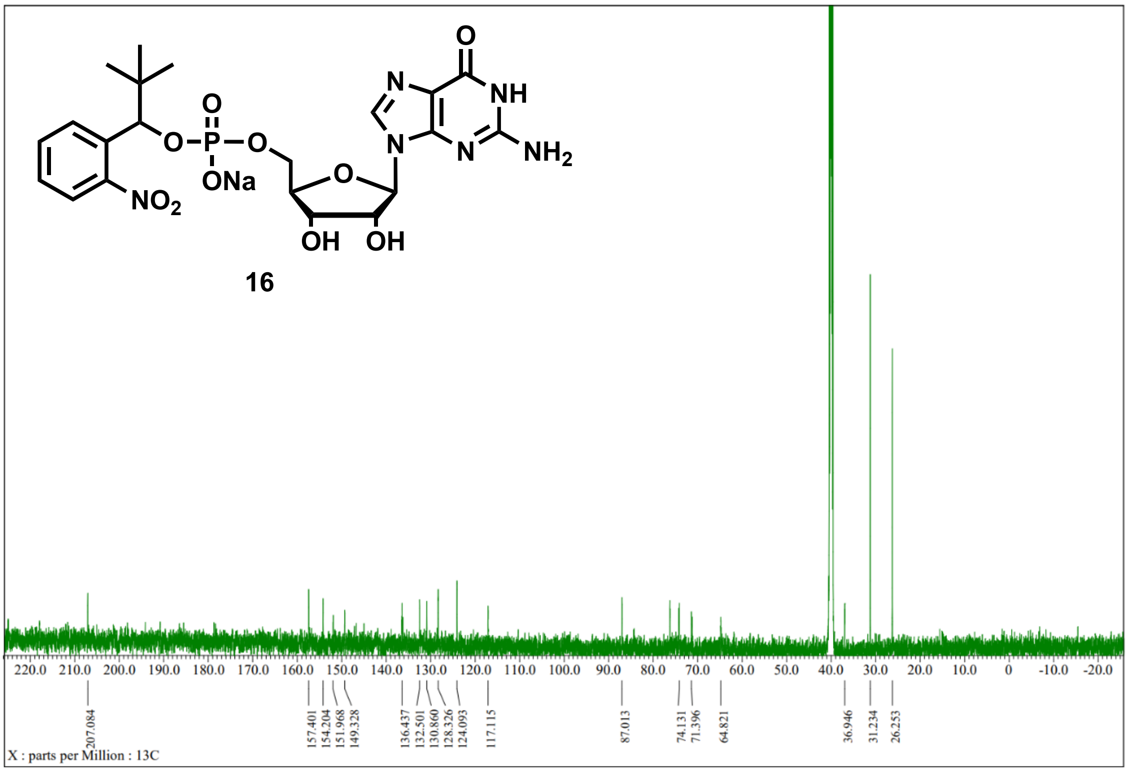

^{31}P NMR Spectrum of Compound **16** (243 MHz, DMSO-d_6)

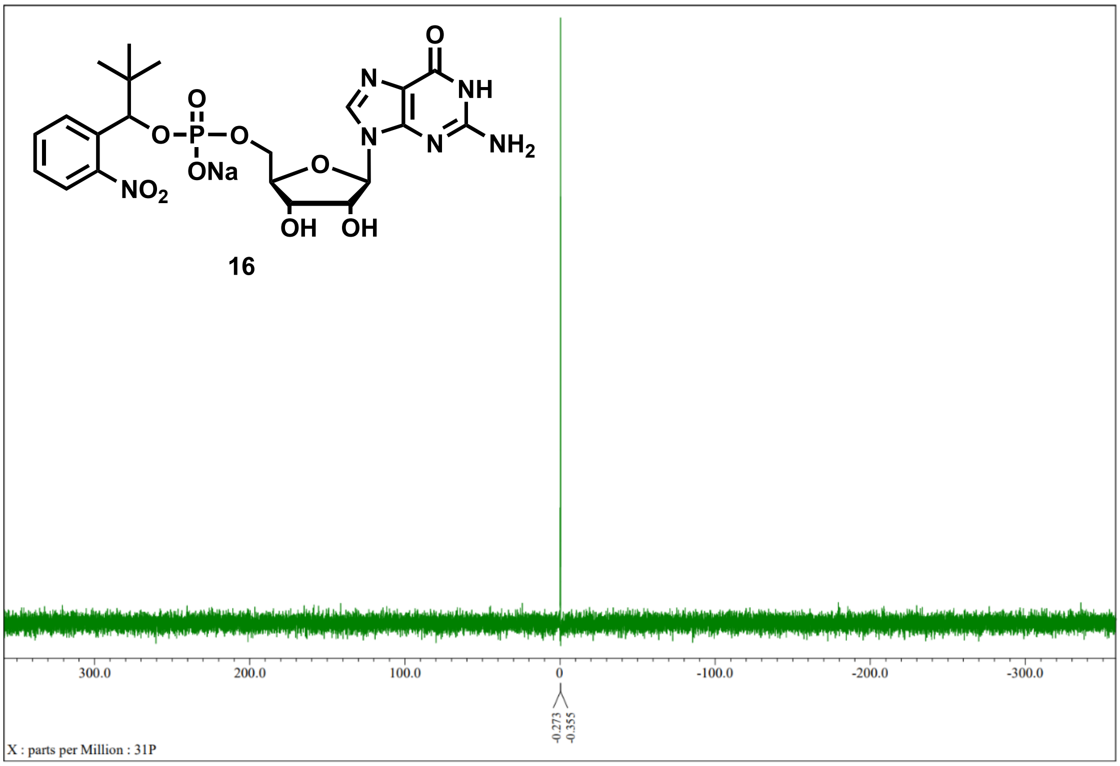

^{1}H NMR Spectrum of Compound **15** (600 MHz, DMSO-d_6)

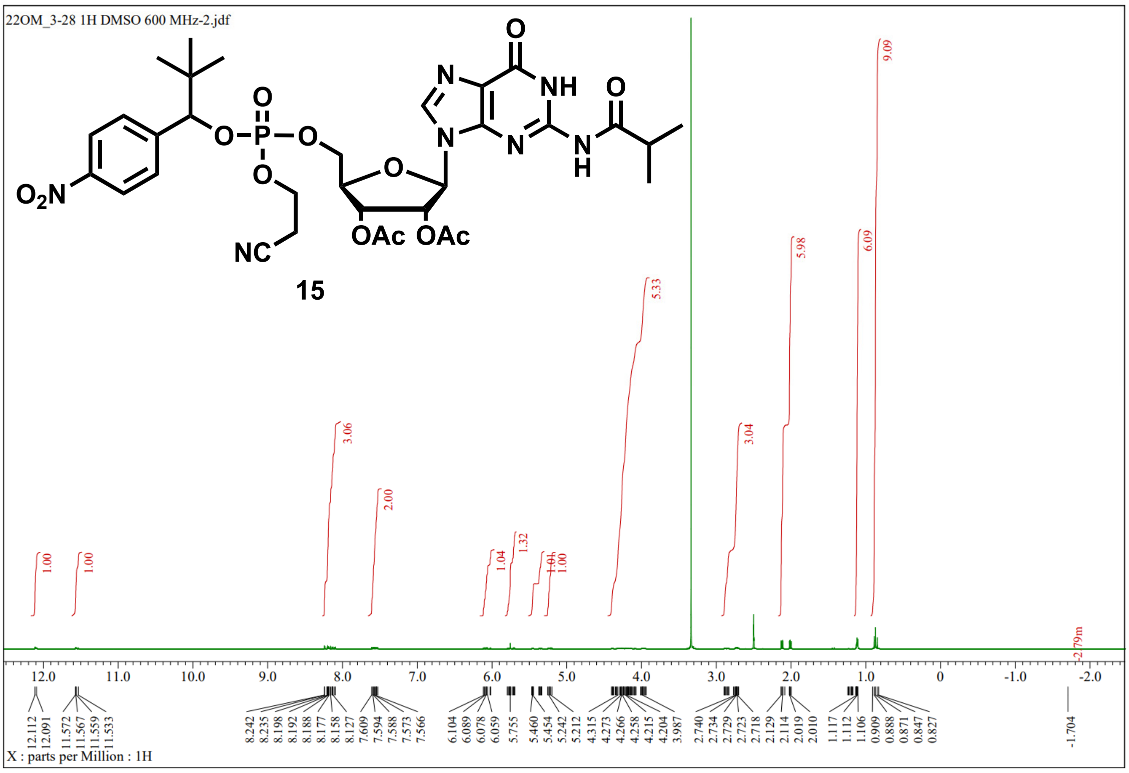

^{13}C NMR Spectrum of Compound **15** (151 MHz, DMSO-d_6)

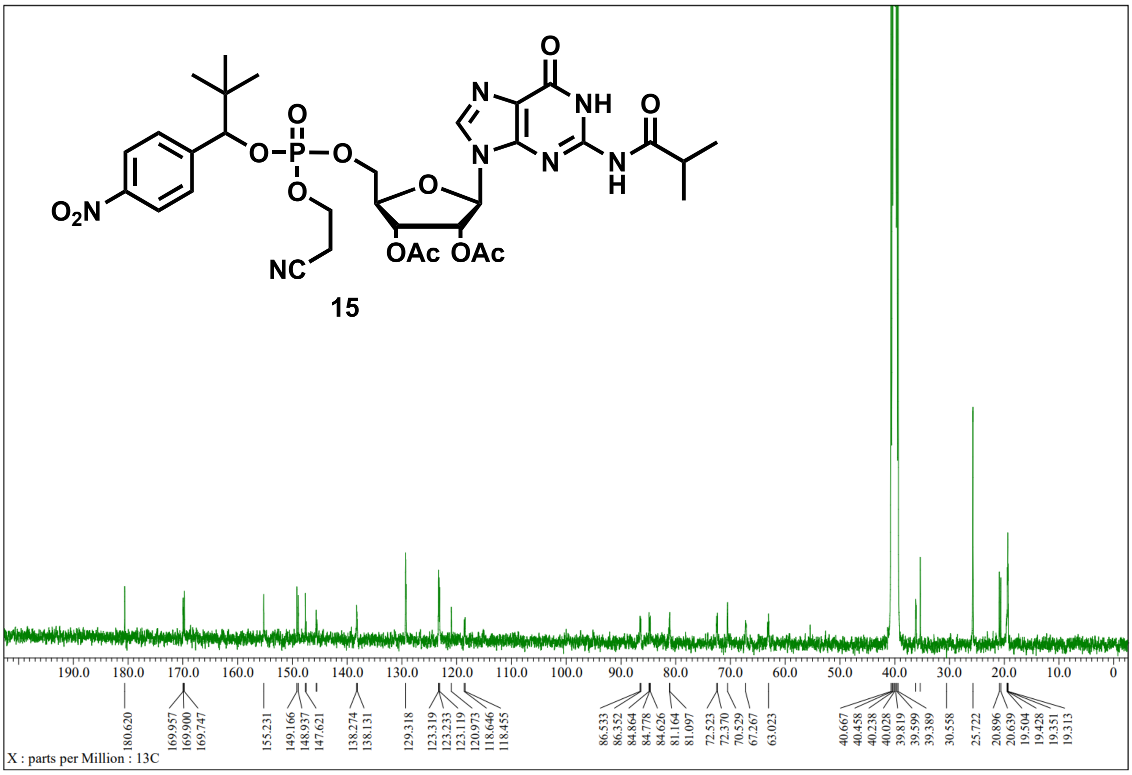

^{31}P NMR Spectrum of Compound **15** (243 MHz, DMSO-d_6)

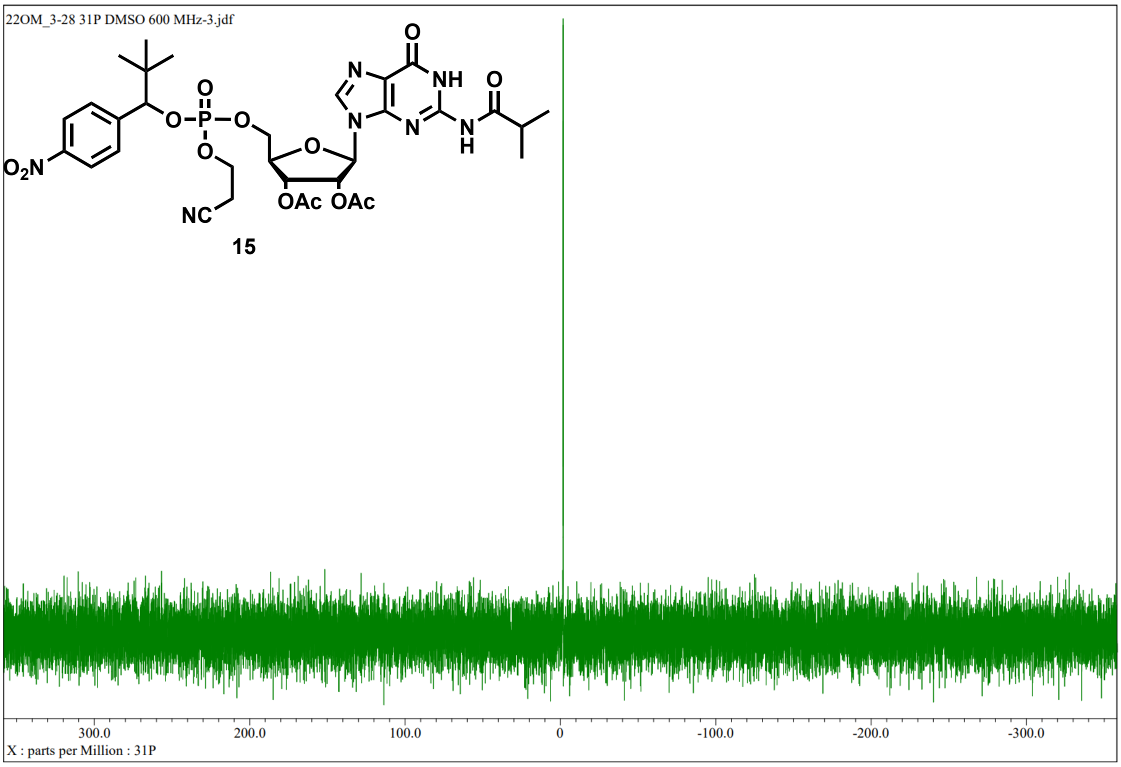

^{1}H NMR Spectrum of Compound **17** (600 MHz, DMSO-d_6)

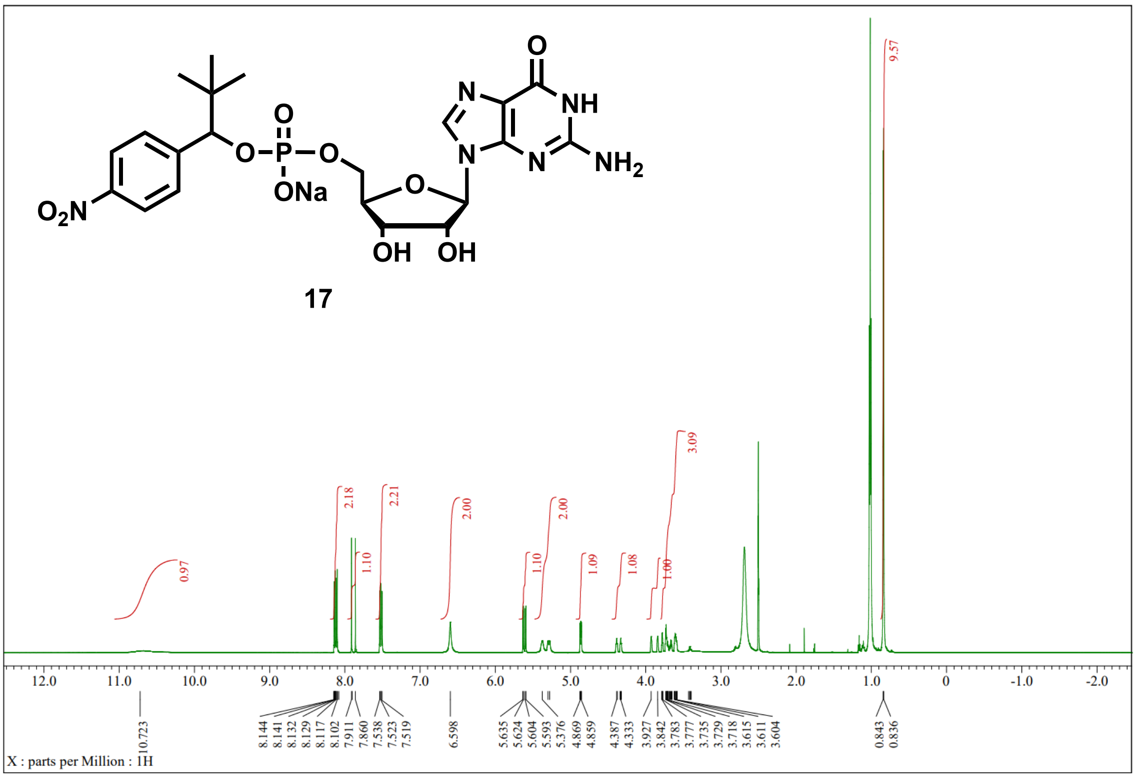

^{13}C NMR Spectrum of Compound **17** (151 MHz, DMSO-d_6)

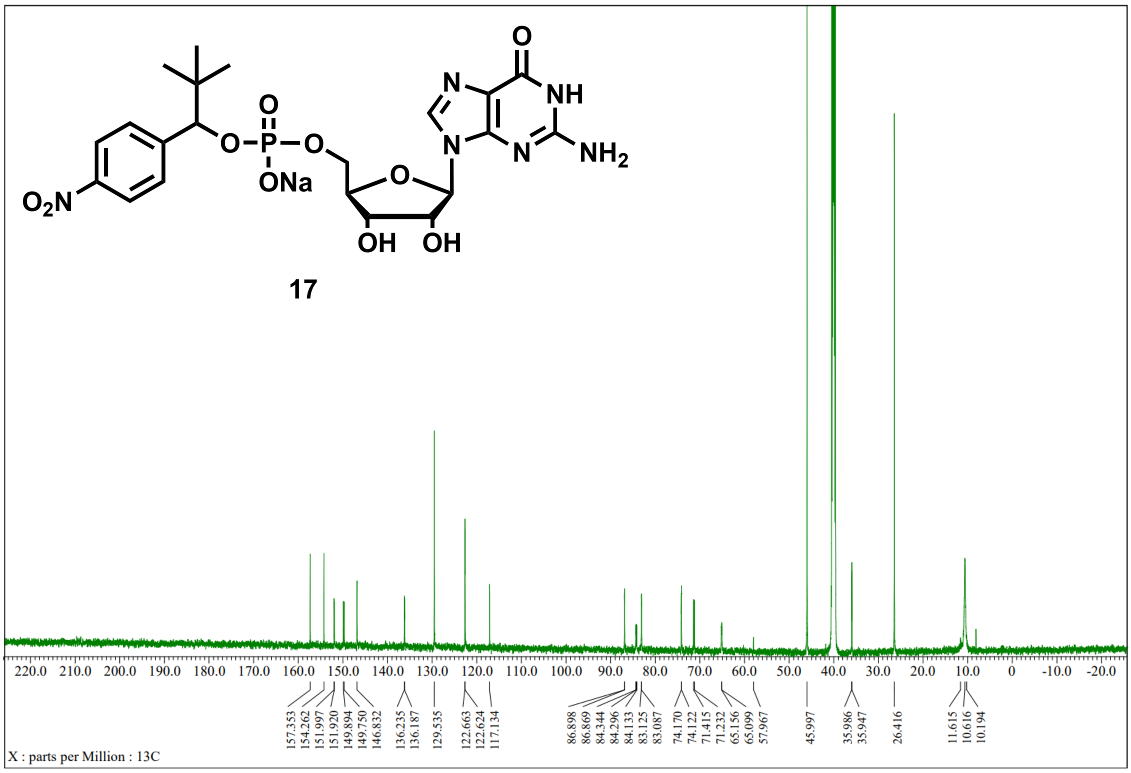

^{31}P NMR Spectrum of Compound **17** (243 MHz, DMSO-d_6)

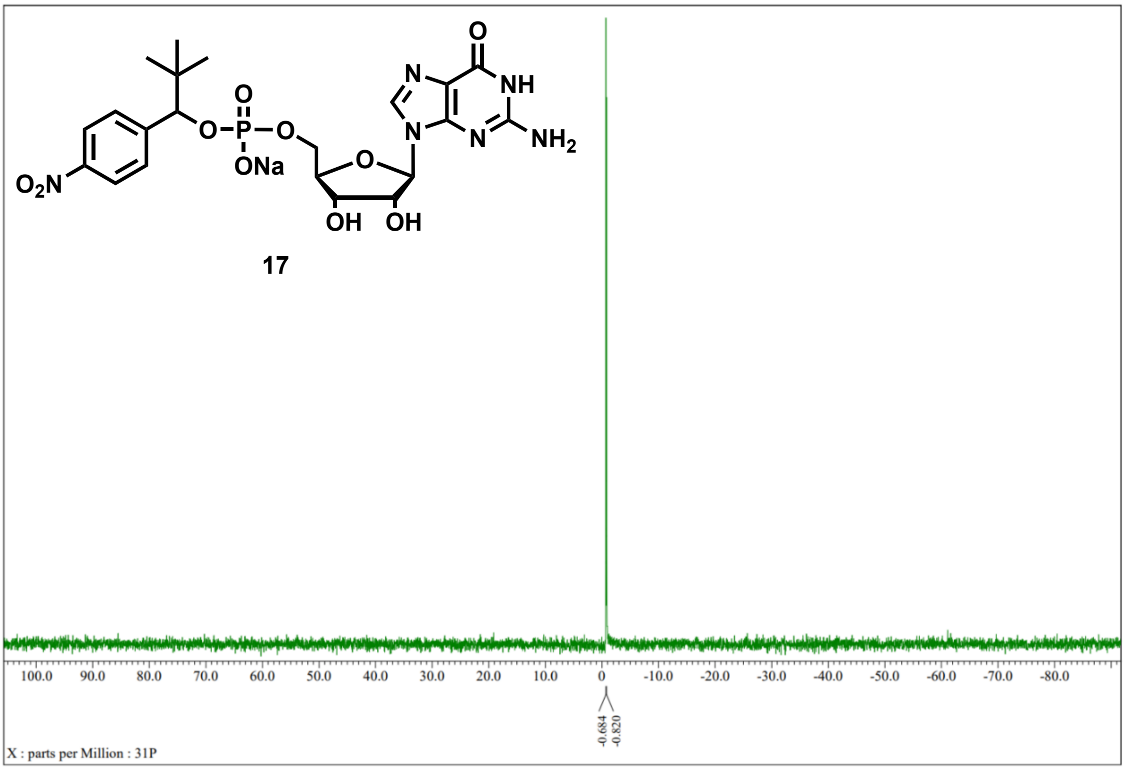

4. HPLC Profiles, MALDI-TOF-MS Spectra, dPAGE Analysis of Synthesized DNAs and RNAs

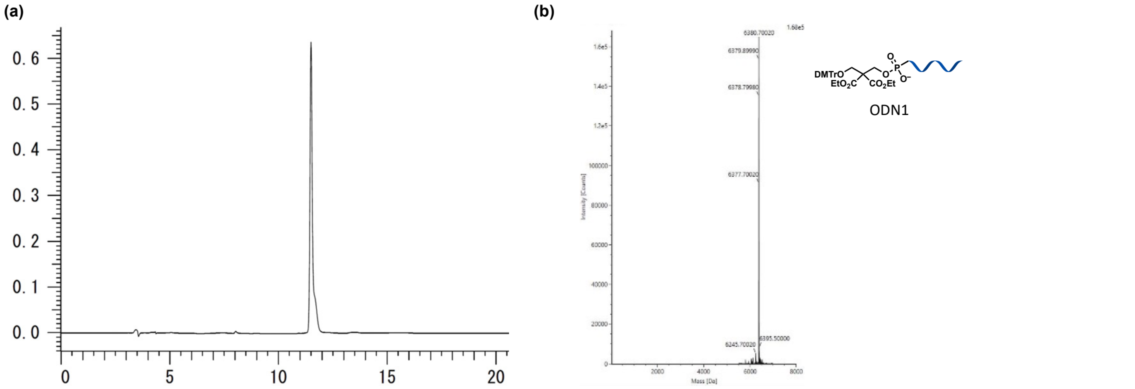

Figure S1. (a) HPLC profile of **ODN1** (Column: Hydrosphere C18 (250 x 4.6 mmI.D., S-5 µm, 12 nm), Solvent A: 50 mM TEAA buffer (pH 7.0) + 5% CH_3CN, Solvent B: CH_3CN, Gradient: 0-100%B over 20 minutes, column temperature 50 °C, Flow rate: 1 mL/min, detection: 254 nm, loop size: 2.0 mL), (b) Deconvoluted mass spectrum of **ODN1** which was collected by using Waters ACQUITY H-Class PLUS_LBNW - Xevo G2-XS Qtof System_NQTW (calcd. 6380. 419 Da, found 6380.700 Da).

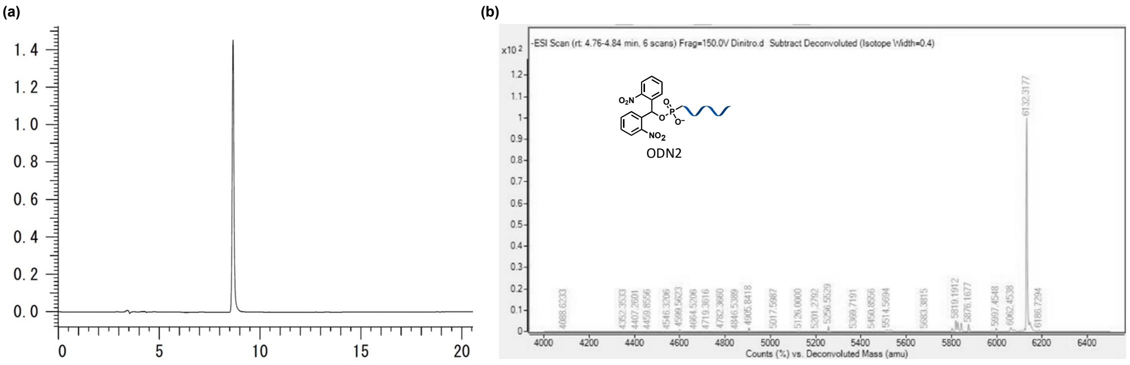

Figure S2. (a) HPLC profile of **ODN2** (Column: Hydrosphere C18 (250 x 4.6 mmI.D., S-5 µm, 12 nm), Solvent A: 50 mM TEAA buffer (pH 7.0) + 5% CH_3CN, Solvent B: CH_3CN, Gradient: 0-100%B over 20 minutes, column temperature 50 °C, Flow rate: 1 mL/min, detection: 254 nm, loop size: 2.0 mL), (b) Deconvoluted mass spectrum of **ODN2** which was collected by using Agilent 1290 Infinity II - 6530 LC/Q-TOF system (calcd. 6,132.057 Da, found 6,132.318 Da).

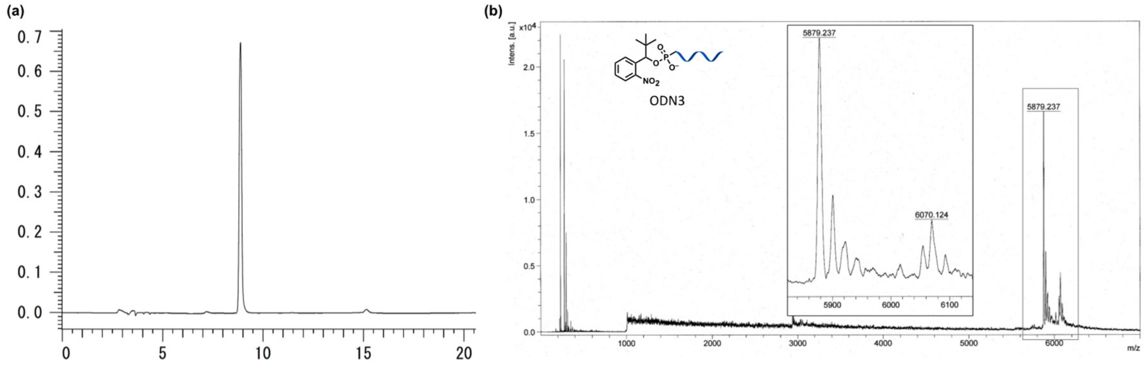

Figure S3. (a) HPLC profile of **ODN3** (Column: Hydrosphere C18 (250 x 4.6 mmI.D., S-5 μm, 12 nm), Solvent A: 50 mM TEAA buffer (pH 7.0) + 5% CH_3CN, Solvent B: CH_3CN, Gradient: 0-100%B over 20 minutes, column temperature 50 °C, Flow rate: 1 mL/min, detection: 254 nm, loop size: 2.0 mL), (b) MALDI-TOF-MS spectrum of **ODN3** (Positive mode $[M + H]^+$, Matrix: 3-HPA, calcd. *m/z* 6,068.077, found *m/z* 6,070.124).

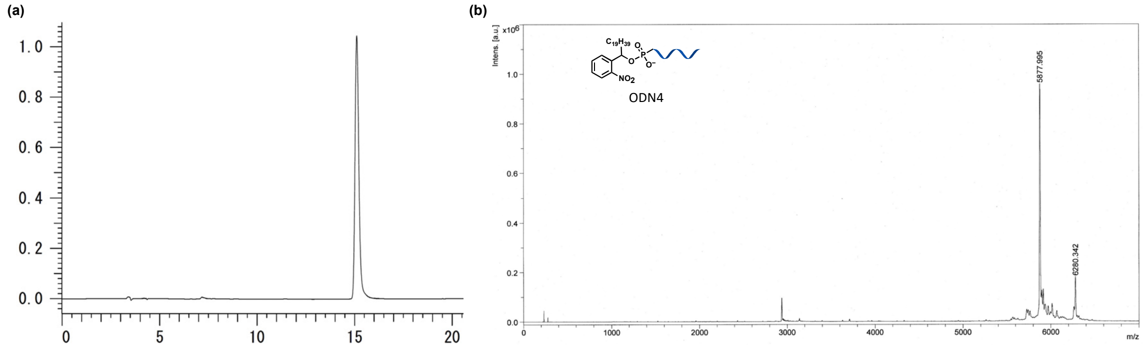

Figure S4. (a) HPLC profile of **ODN4** (Column: Hydrosphere C18 (250 x 4.6 mmI.D., S-5 μm, 12 nm), Solvent A: 50 mM TEAA buffer (pH 7.0) + 5% CH_3CN, Solvent B: CH_3CN, Gradient: 0-100%B over 20 minutes, column temperature 50 °C, Flow rate: 1 mL/min, detection: 254 nm, loop size: 2.0 mL), (b) MALDI-TOF-MS spectrum of **ODN4** (Positive mode $[M + H]^+$, Matrix: 3-HPA, calcd. *m/z* 6,278.482, found *m/z* 6,280.342).

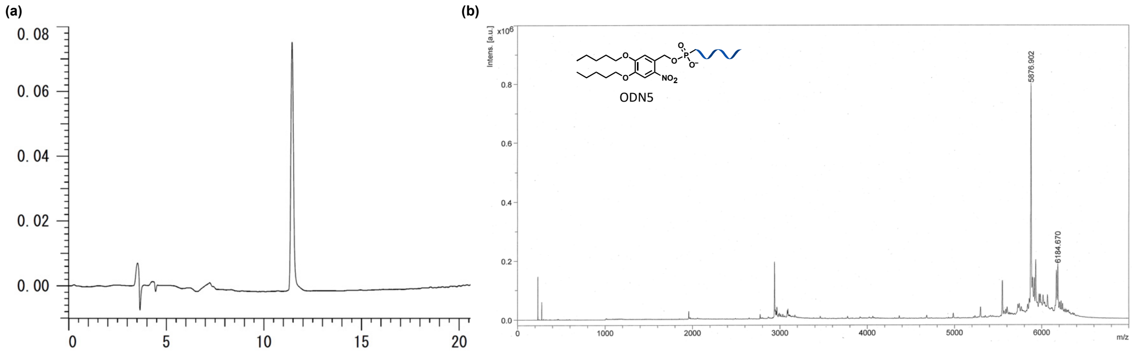

Figure S5. (a) HPLC profile of **ODN5** (Column: Hydrosphere C18 (250 x 4.6 mmI.D., S-5 μm, 12 nm), Solvent A: 50 mM TEAA buffer (pH 7.0) + 5% CH_3CN, Solvent B: CH_3CN, Gradient: 0-100%B over 20 minutes, column temperature 50 °C, Flow rate: 1 mL/min, detection: 254 nm, loop size:

2.0 mL), (b) MALDI-TOF-MS spectrum of **ODN5** (Positive mode $[M + H]^+$, Matrix: 3-HPA, calcd. *m/z* 6,184.237, found *m/z* 6,184.670).

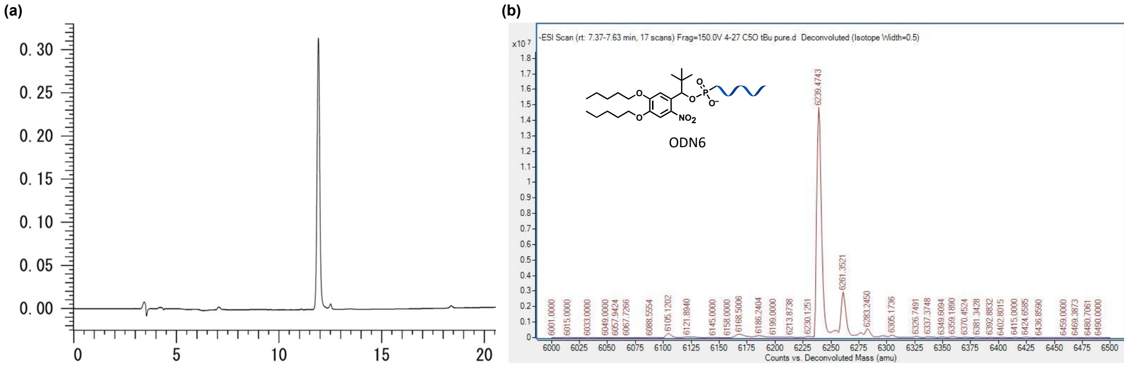

Figure S6. (a) HPLC profile of **ODN6** (Column: Hydrosphere C18 (250 x 4.6 mmI.D., S-5 µm, 12 nm), Solvent A: 50 mM TEAA buffer (pH 7.0) + 5% CH_3CN, Solvent B: CH_3CN, Gradient: 0-100%B over 20 minutes, column temperature 50 °C, Flow rate: 1 mL/min, detection: 254 nm, loop size: 2.0 mL), (b) Deconvoluted mass spectrum of **ODN6** which was collected by using Agilent 1290 Infinity II - 6530 LC/Q-TOF system (calcd. 6,239.337 Da, found 6,239.474 Da).

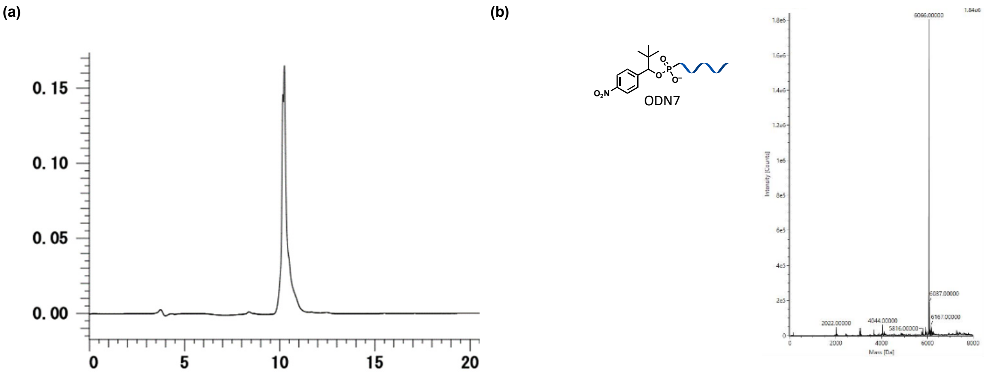

Figure S7. (a) HPLC profile of **ODN7** (Column: Hydrosphere C18 (250 x 4.6 mmI.D., S-5 µm, 12 nm), Solvent A: 50 mM TEAA buffer (pH 7.0) + 5% CH_3CN, Solvent B: CH_3CN, Gradient: 0-100%B over 20 minutes, column temperature 50 °C, Flow rate: 1 mL/min, detection: 254 nm, loop size: 2.0 mL), (b) Deconvoluted mass spectrum of **ODN7** which was collected by using Waters ACQUITY H-Class PLUS_LBNW - Xevo G2-XS Qtof System_NQTW (calcd. 6067.077 Da, found 6066.000 Da).

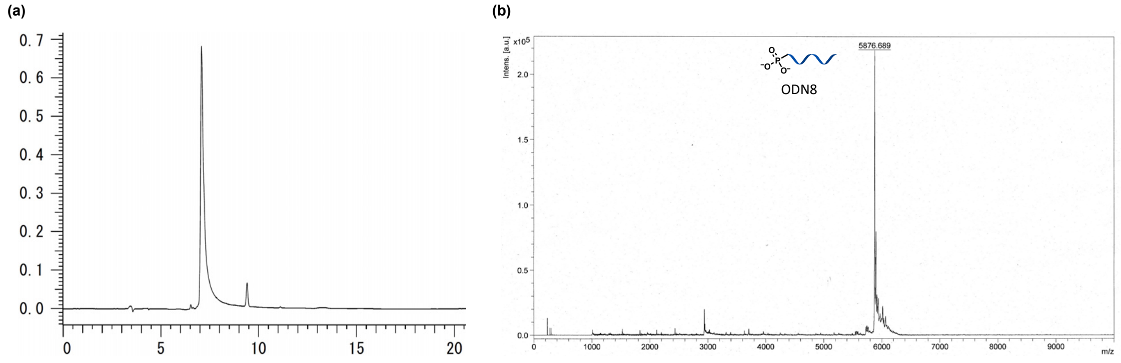

Figure S8. (a) HPLC profile of **ODN8** (Column: Hydrosphere C18 (250 x 4.6 mmI.D., S-5 µm, 12 nm), Solvent A: 50 mM TEAA buffer (pH 7.0) + 5% CH_3CN, Solvent B: CH_3CN, Gradient: 0-100%B over 20 minutes, column temperature 50 °C, Flow rate: 1 mL/min, detection: 254 nm, loop size: 2.0 mL) (b) MALDI-TOF-MS spectrum of **ODN8** (Positive mode $[M + H]^+$, Matrix: 3-HPA, calcd. *m/z* 5,876.847, found *m/z* 5876.689).

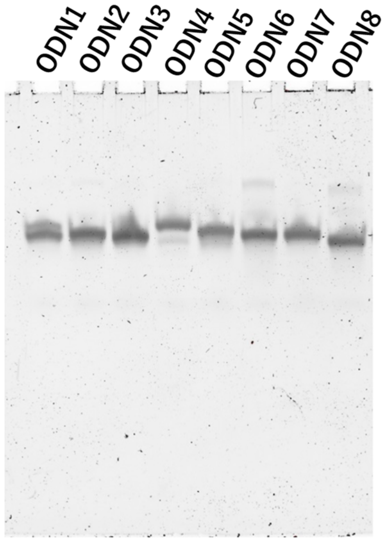

Figure S9. 20% dPAGE analysis of synthesized **ODN1–8** (20mm constant for 30 minutes).

5. Investigation of Deprotection Conditions of Nitrobenzyl Groups

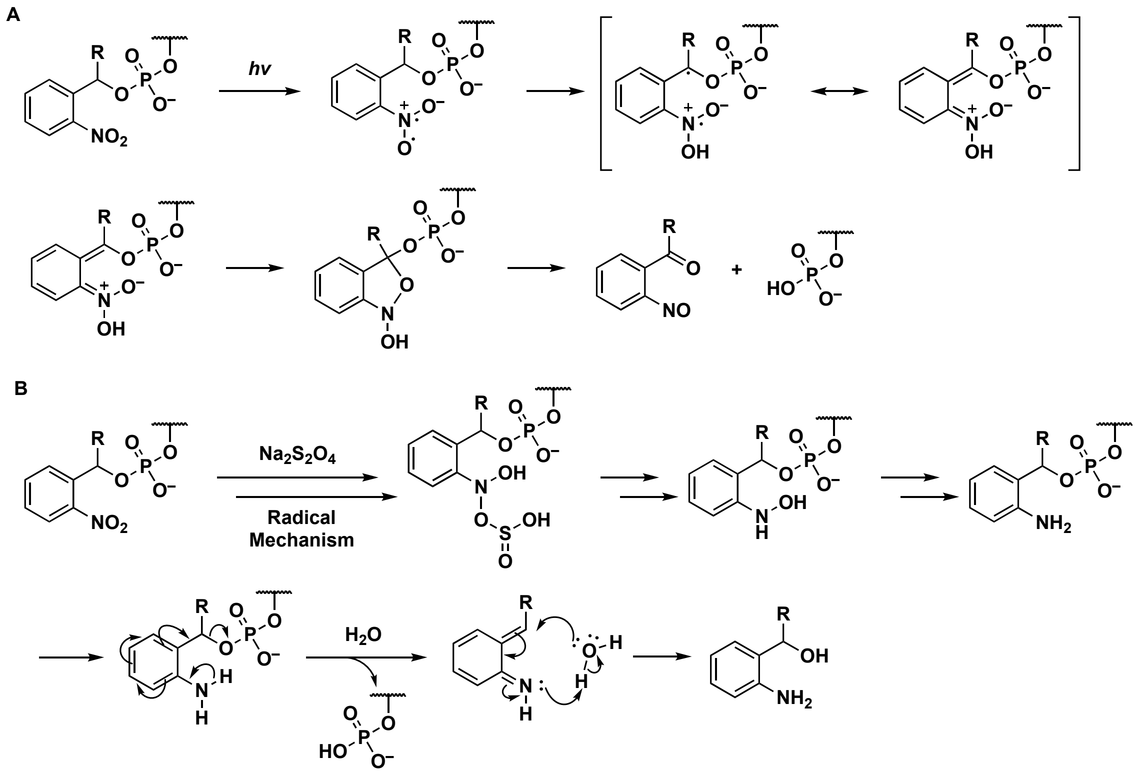

Scheme S8. Deprotection mechanism of nitrobenzyl group by (A) UV-irradiation (6,7) and (B) sodium dithionate treatment (8).

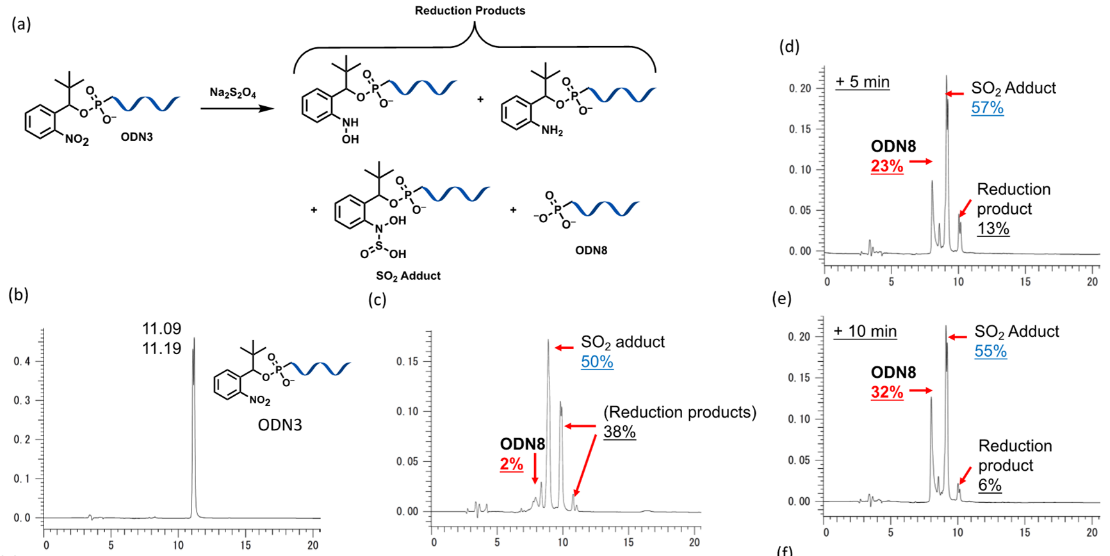

(g)

Deprotection Condition	ODN8 (%)	SO_2 Adduct (%)	Reduction product (%)
37ºC, 30 min	2	50	38
37ºC, 30 min + 95ºC, 5 min	23	57	13
37ºC, 30 min + 95ºC, 10 min	32	55	6
37ºC, 30 min + 95ºC, 20 min	46	54	0

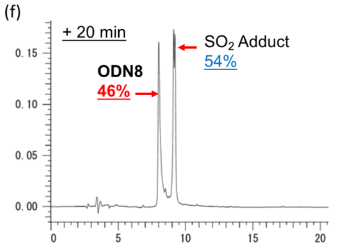

Figure S10. HPLC profiles of the reaction mixtures to investigate reductive deprotection of *o*-nitrobenzyl group (HPLC condition, Column: Hydrosphere C18 (250 x 4.6 mmI.D., S-5 μm, 12 nm), Solvent A: 50 mM TEAA buffer (pH 7.0) + 5% CH_3CN, Solvent B: CH_3CN, Gradient: 0-60%B over 20 minutes, column temperature 50 °C, Flow rate: 1 mL/min, detection: 254 nm, loop size: 2.0 mL), (a) Reaction scheme, (b) HPLC profile of **ODN3**, (c) HPLC profile of the reaction mixture of the reduction of **ODN3** by $Na_2S_2O_4$ (1,000 eq.) at 37 °C for 30 minutes, (d) HPLC profile of the reaction mixture of **ODN3** by $Na_2S_2O_4$ (1,000 eq.) at 37 °C for 30 minutes + 95 °C for 5 minutes , (e) HPLC profile of the reaction mixture of **ODN3** by $Na_2S_2O_4$ (1,000 eq.) at 37 °C for 30 minutes + 95 °C for 10 minutes, (f) HPLC profile of the reaction mixture of **ODN3** by $Na_2S_2O_4$ (1,000 eq.) at 37 °C for 30 minutes + 95 °C for 20 minutes, (g) HPLC peak analysis.

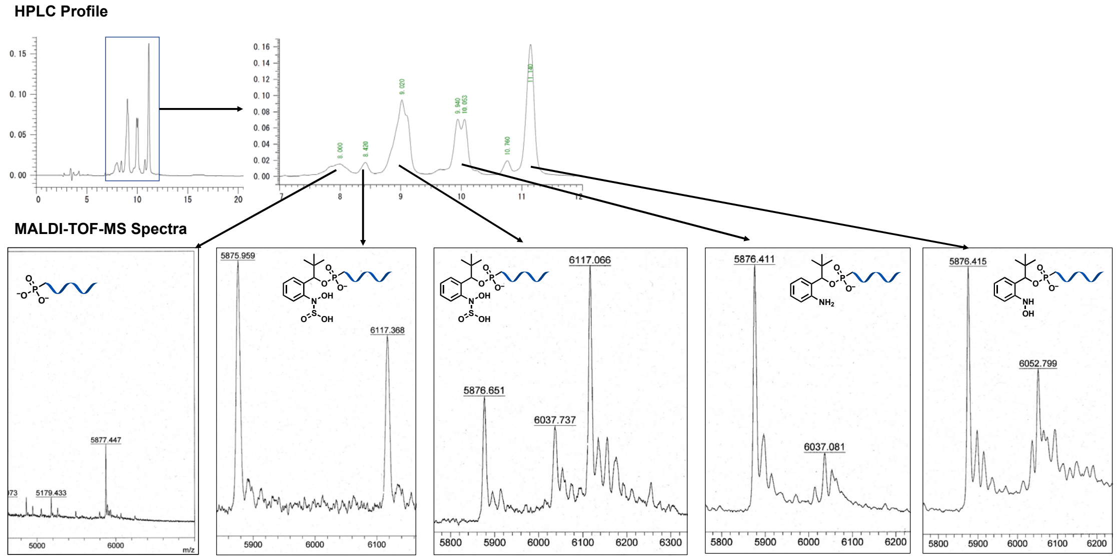

Figure S11. HPLC profile of the reaction mixture of the reductive deprotection of **ODN3** by $Na_2S_2O_4$ (500 eq.) at room temperature for 30 minutes (HPLC condition, Column: Hydrosphere C18 (250 x 4.6 mmI.D., S-5 μm, 12 nm), Solvent A: 50 mM TEAA buffer (pH 7.0) + 5% CH_3CN, Solvent B: CH_3CN, Gradient: 0-60%B over 20 minutes, column temperature 50 °C, Flow rate: 1 mL/min, detection: 254 nm, loop size: 2.0 mL). MALDI-TOF-MS spectra of each HPLC peaks (Linear Positive mode, Matrix: 3-HPA).

Table S1. List of reduction products of ODN3 by $Na_2S_2O_4$ treatment (500 eq., room temperature, 30 minutes).

		Mass / Da	
Structure	Retention Time / minutes	Calcd.	found
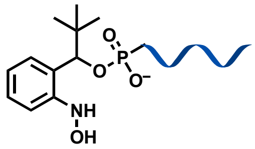	11.0	6,054.094	6,052.799
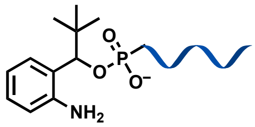	10.0	6,038.095	6,037.081
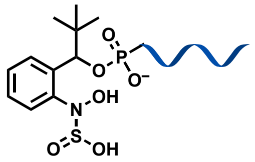	9.0, 8.5	6,118.152	6,117.066
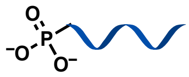	8.0	5,876.847	5,877.447

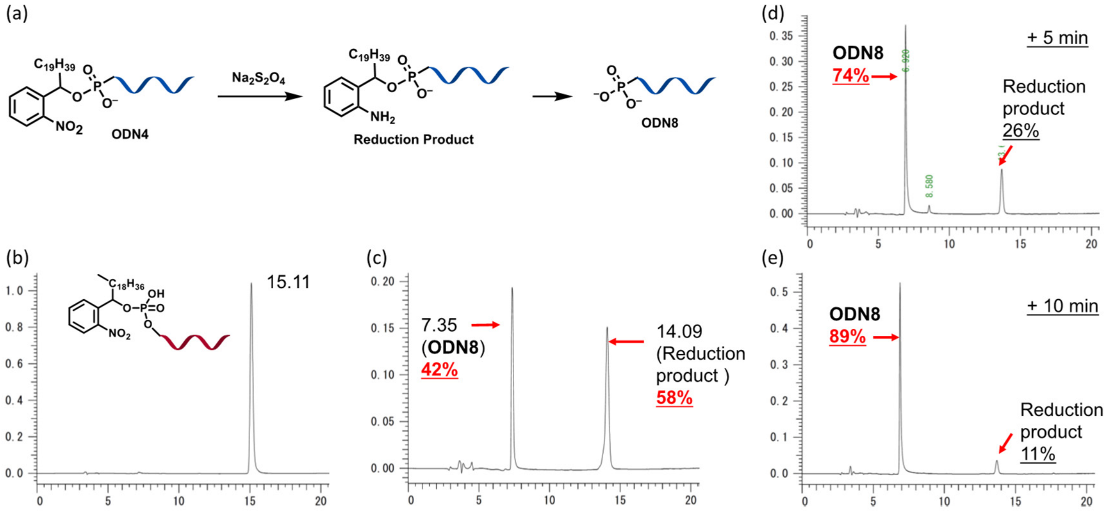

(g)

Deprotection Condition	ODN8 (%)	Reduction product (%)
37°C, 30 min	42	58
37°C, 30 min + 95°C, 5 min	74	26
37°C, 30 min + 95°C, 10 min	89	11
37°C, 30 min + 95°C, 20 min	98	2

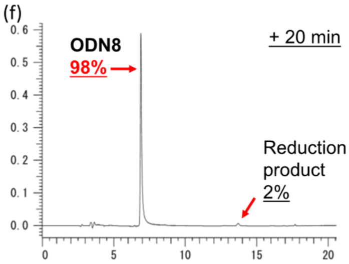

Figure S12. HPLC profiles of the reaction mixtures to investigate reductive deprotection of *o*-nitrobenzyl group (HPLC condition, Column: Hydrosphere C18 (250 x 4.6 mmI.D., S-5 μm, 12 nm), Solvent A: 50 mM TEAA buffer (pH 7.0) + 5% CH_3CN, Solvent B: CH_3CN, Gradient: 0-100%B over 20 minutes, column temperature 50 °C, Flow rate: 1 mL/min, detection: 254 nm, loop size: 2.0 mL), (a) Reaction scheme, (b) HPLC profile of **ODN4**, (c) HPLC profile of the reaction mixture of the reduction of **ODN4** by $Na_2S_2O_4$ (1,000 eq.) at 37 °C for 30 minutes, (d) HPLC profile of the reaction mixture of **ODN4** by $Na_2S_2O_4$ (1,000 eq.) at 37 °C for 30 minutes + 95 °C for 5 minutes , (e) HPLC profile of the reaction mixture of **ODN4** by $Na_2S_2O_4$ (1,000 eq.) at 37 °C for 30 minutes + 95 °C for 10 minutes, (f) HPLC profile of the reaction mixture of **ODN4** by $Na_2S_2O_4$ (1,000 eq.) at 37 °C for 30 minutes + 95 °C for 20 minutes, (g) HPLC peak analysis.

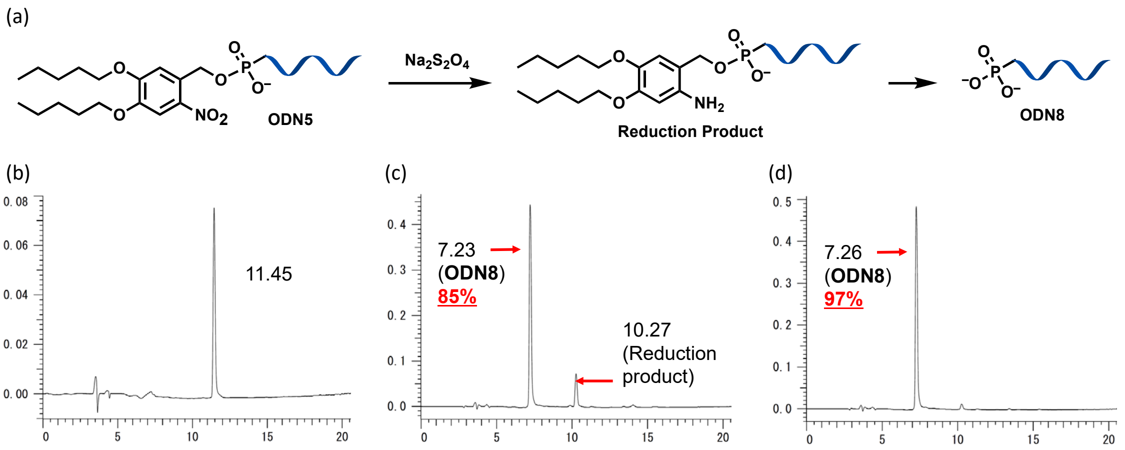

Figure S13. HPLC profiles of the reaction mixtures to investigate reductive deprotection of *o*-nitrobenzyl group (HPLC condition, Column: Hydrosphere C18 (250 x 4.6 mmI.D., S-5 μm, 12 nm), Solvent A: 50 mM TEAA buffer (pH 7.0) + 5% CH_3CN, Solvent B: CH_3CN, Gradient: 0-100%B over 20 minutes, column temperature 50 °C, Flow rate: 1 mL/min, detection: 254 nm, loop size: 2.0 mL), (a) Reaction scheme, (b) HPLC profile of **ODN5**, (c) HPLC profile of the reaction mixture of the reduction of **ODN5** by $Na_2S_2O_4$ (1,000 eq.) at 37 °C for 30 minutes, (d) HPLC profile of the reaction mixture of **ODN5** by $Na_2S_2O_4$ (1,000 eq.) at 37 °C for 30 minutes + 65 °C for 10 minutes.

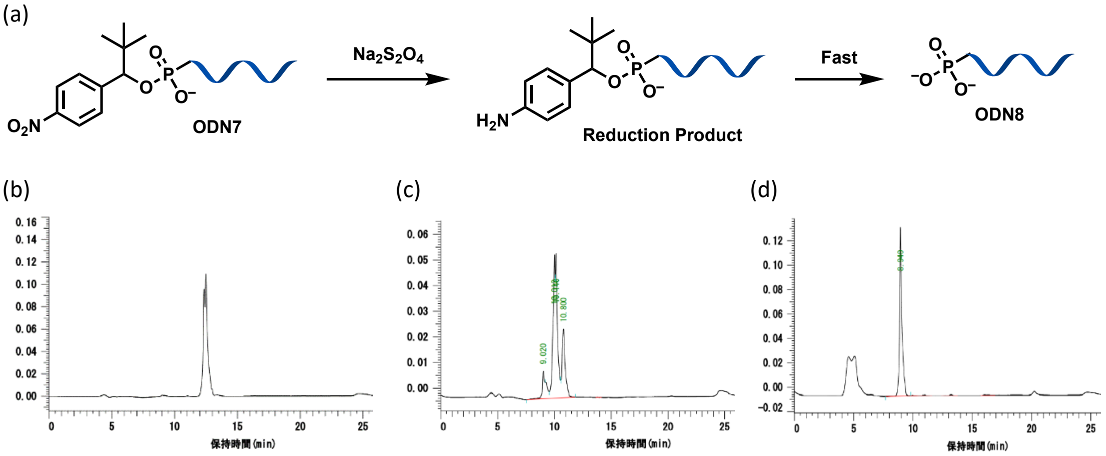

Figure S14. HPLC profiles of the reaction mixtures to investigate reductive deprotection of *p*-nitrobenzyl group (HPLC condition, Column: Hydrosphere C18 (250 x 4.6 mmI.D., S-5 μm, 12 nm), Solvent A: 50 mM TEAA buffer (pH 7.0) + 5% CH_3CN, Solvent B: CH_3CN, Gradient: 0-100%B over 20 minutes, column temperature 50 °C, Flow rate: 1 mL/min, detection: 254 nm, loop size: 2.0 mL), (a) Reaction scheme, (b) HPLC profile of **ODN7**, (c) HPLC profile of the reaction mixture of the reduction of **ODN7** by $Na_2S_2O_4$ (1,000 eq.) at 37 °C for 30 minutes, (d) HPLC profile of the reaction mixture of **ODN7** by $Na_2S_2O_4$ (1,000 eq.) at 37 °C for 30 minutes + 50 °C for 120 minutes.

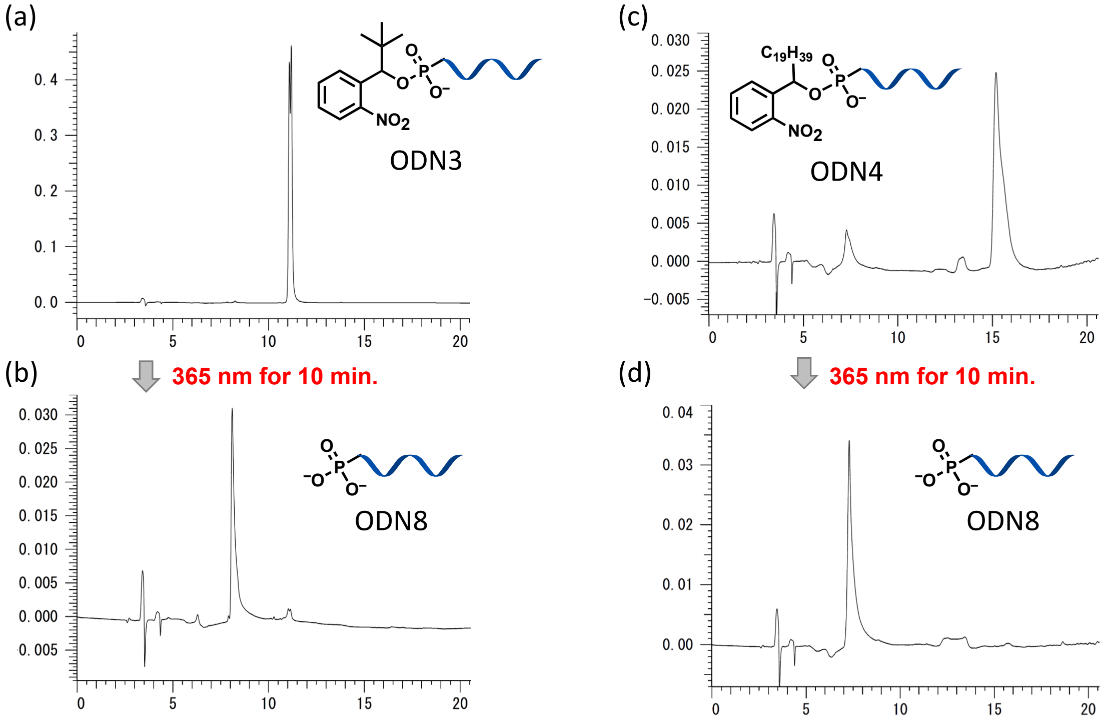

Figure S15. HPLC profiles of the reaction mixture to investigate UV-induced deprotection of o-nitrobenzyl group. (a) HPLC profile of **ODN3** (HPLC condition, Column: Hydrosphere C18 (250 x 4.6 mmI.D., S-5 μm, 12 nm), Solvent A: 50 mM TEAA buffer (pH 7.0) + 5% CH_3CN, Solvent B: CH_3CN, Gradient: 0-60%B over 20 minutes, column temperature 50 °C, Flow rate: 1 mL/min, detection: 254 nm, loop size: 2.0 mL), (b) HPLC profile of the produced **ODN8** by UV-irradiation of **ODN3** (HPLC condition, Column: Hydrosphere C18 (250 x 4.6 mmI.D., S-5 μm, 12 nm), Solvent A: 50 mM TEAA buffer (pH 7.0) + 5% CH_3CN, Solvent B: CH_3CN, Gradient: 0-60%B over 20 minutes, column temperature 50 °C, Flow rate: 1 mL/min, detection: 254 nm, loop size: 2.0 mL). (c) HPLC profile of **ODN4** (HPLC condition, Column: Hydrosphere C18 (250 x 4.6 mmI.D., S-5 μm, 12 nm), Solvent A: 50 mM TEAA buffer (pH 7.0) + 5% CH_3CN, Solvent B: CH_3CN, Gradient: 0-100%B over 20 minutes, column temperature 50 °C, Flow rate: 1 mL/min, detection: 254 nm, loop size: 2.0 mL), (d) HPLC profile of the produced **ODN8** by UV-irradiation of **ODN4** (HPLC condition, Column: Hydrosphere C18 (250 x 4.6 mmI.D., S-5 μm, 12 nm), Solvent A: 50 mM TEAA buffer (pH 7.0) + 5% CH_3CN, Solvent B: CH_3CN, Gradient: 0-100%B over 20 minutes, column temperature 50 °C, Flow rate: 1 mL/min, detection: 254 nm, loop size: 2.0 mL).

6. Spectral Data of Chemically Synthesized RNAs

Oligoribonucleotide (ORNs) were synthesized and 5’-phosphorylated by using phosphoramidite (**3**).

Table S2. List of Chemically Synthesized RNAs.

ORN	Sequence (5’→3’)*	5’-Structure	Retention Time	Mass / Da Calcd.	Mass / Da found
**ORN1**	**(HiBiT)**	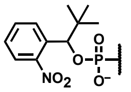	10.11 min	22,447.899	22,446.600
**ORN2**	A_mG_mAGCCACCAUGGU GAGCGGCUGGCGGCUG UUCAAGAAGAUUAGCU	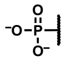	8.53 min	22,256.669	22,256.301
**ORN3**	GAAAAAAAAAAAAAA AAAAAAA	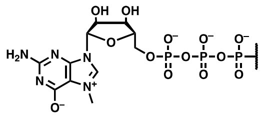	8.57 min	22,695.882	22,695.199

*A_m and G_m: 2’-*O*-Methyladenosine and 2’-*O*-Methylguanosine

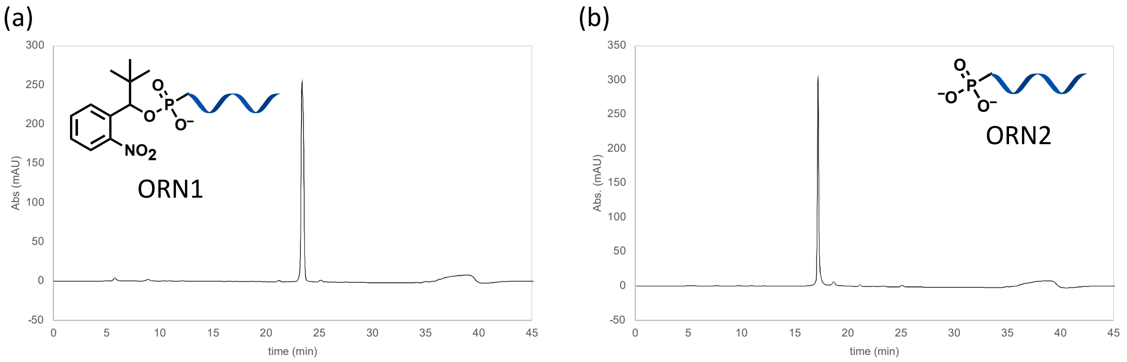

Figure S16. HPLC profiles of (a) **ORN1** and (b) produced **ORN2** by UV-irradiation of **ORN1** (HPLC condition, Column: YMC-Triart bio C4 (250 x 4.6 mmI.D., S-5 µm, 30 nm), Solvent A: 50 mM TEAA buffer (pH 7.0) + 5% CH_3CN, Solvent B: CH_3CN, Gradient: 0-25%B over 25 minutes, column temperature 50 °C, Flow rate: 1 mL/min, detection: 254 nm, loop size: 2.0 mL).

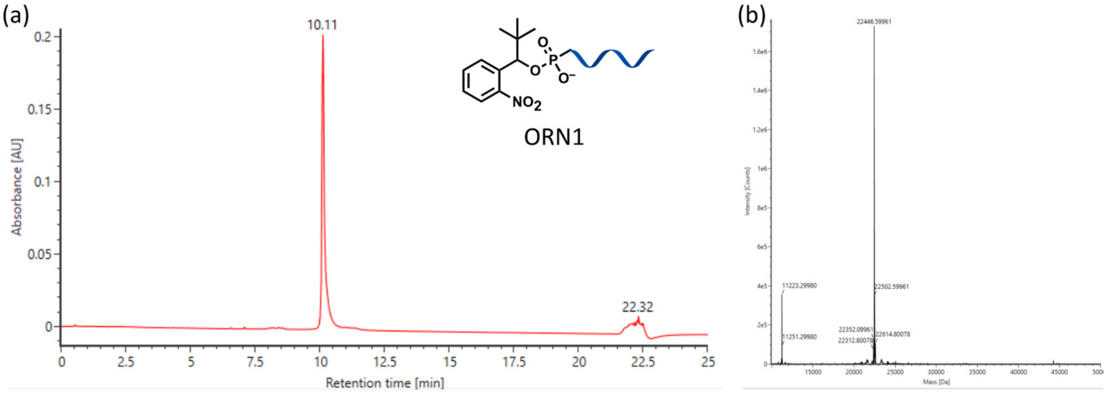

Figure S17. LC-MS analysis of **ORN1**, (a) UPLC Profile, (b) Deconvoluted mass spectrum**.** UPLC System: Waters ACQUITY H-Class PLUS_LBNW, MS System: Waters Xevo G2-XS Qtof System_NQTW, ACQUITY UPLC BEH C18 Column, 130Å, 1.7 µm, 2.1 mm X 50 mm, Part No.186002350, Serial No. 04063126735169, Sol A; 100 mM 1,1,1,3,3,3,-hexafluoropropan-2-ol (HFIP), 8.6 mM TEA (pH 8.3), Sol B; 100% MeOH, Column Temp.: 60 deg., Detection: 260 nm, Flow rate: 0.3 mL/min, Gradient Program: 5–10%B (0–0.5 minutes), 10–30%B (0.5–20 minutes), 30–80%B (20.0–20.01 minutes), 80%B (20.01–21.00 minutes), 80–5%B (21.00–21.01 minutes), 5%B (21.01–25.00 minutes).

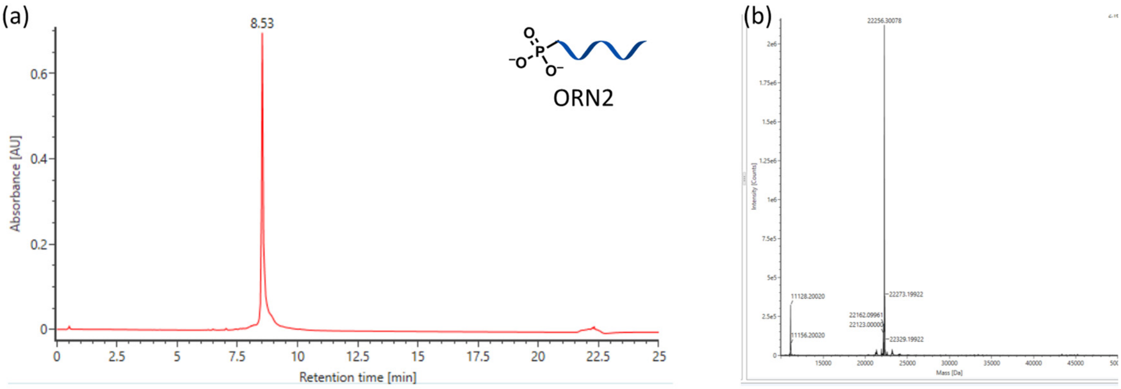

Figure S18. LC-MS analysis of **ORN2**, (a) UPLC Profile, (b) Deconvoluted mass spectrum**.** UPLC System: Waters ACQUITY H-Class PLUS_LBNW, MS System: Waters Xevo G2-XS Qtof System_NQTW, ACQUITY UPLC BEH C18 Column, 130Å, 1.7 µm, 2.1 mm X 50 mm, Part No.186002350, Serial No. 04063126735169, Sol A; 100 mM 1,1,1,3,3,3,-hexafluoropropan-2-ol (HFIP), 8.6 mM TEA (pH 8.3), Sol B; 100% MeOH, Column Temp.: 60 deg., Detection: 260 nm, Flow rate: 0.3 mL/min, Gradient Program: 5–10%B (0–0.5 minutes), 10–30%B (0.5–20 minutes), 30–80%B (20.0–20.01 minutes), 80%B (20.01–21.00 minutes), 80–5%B (21.00–21.01 minutes), 5%B (21.01–25.00 minutes).

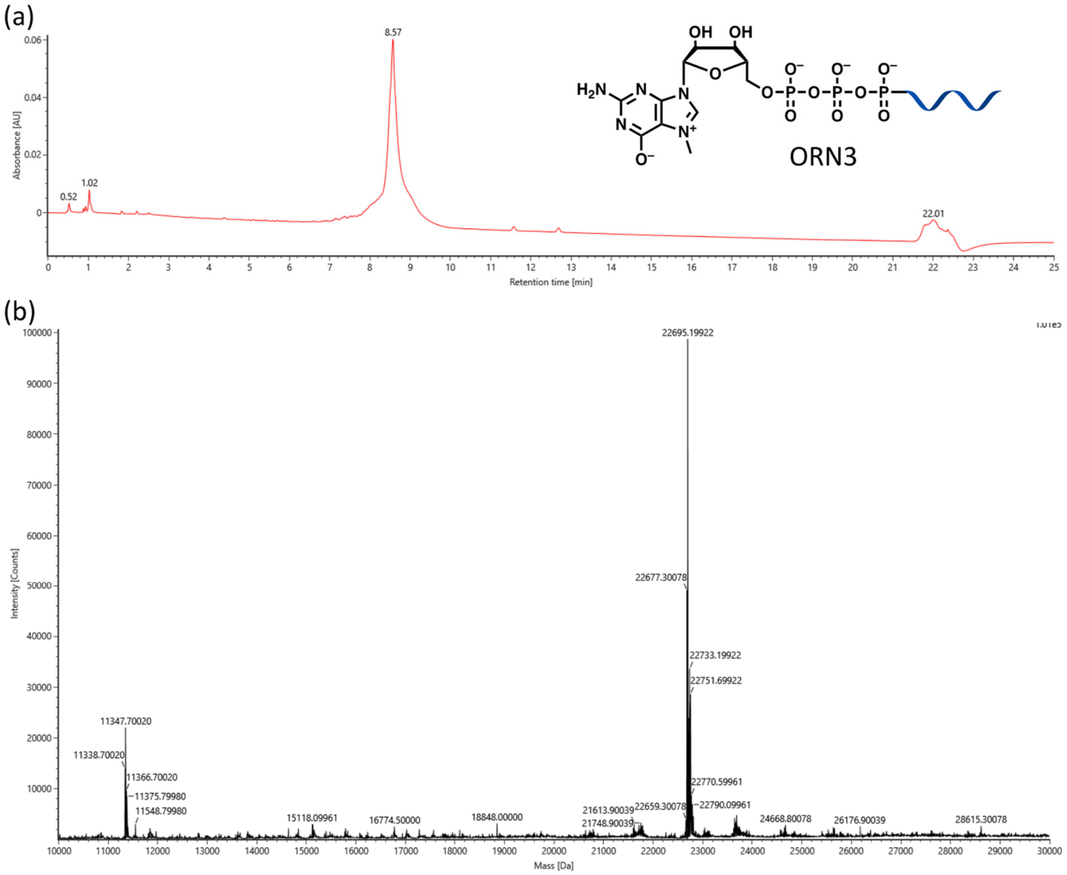

Figure S19. LC-MS analysis of **ORN3**, (a) UPLC Profile, (b) Deconvoluted mass spectrum**.** UPLC System: Waters ACQUITY H-Class PLUS_LBNW, MS System: Waters Xevo G2-XS Qtof System_NQTW, ACQUITY UPLC BEH C18 Column, 130Å, 1.7 µm, 2.1 mm X 50 mm, Part No.186002350, Serial No. 04063126735169, Sol A; 100 mM 1,1,1,3,3,3,-hexafluoropropan-2-ol (HFIP), 8.6 mM TEA (pH 8.3), Sol B; 100% MeOH, Column Temp.: 60 deg., Detection: 260 nm, Flow rate: 0.3 mL/min, Gradient Program: 5–10%B (0–0.5 minutes), 10–30%B (0.5–20 minutes), 30–80%B (20.0–20.01 minutes), 80%B (20.01–21.00 minutes), 80–5%B (21.00–21.01 minutes), 5%B (21.01–25.00 minutes).

Table S3. List of Chemically Synthesized RNAs.

ORN	Length	Sequence (5’→3’)*	5’-Structure	Retention Time (minutes)
**ORN4**	107	G_mG_mAGCCACCAUGGACUACAAGGACGACGACG AUAAGAUCAUCAGCUAUAAAGACGACGACGAUA AACACCACCACCACCACCACUGAAAAAAAAAAA AAAAAAAAAA	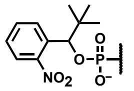	16.60
**ORN5**			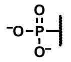	11.77
**ORN6**	131	G_mG_mAGCCACCAUGGGAUGGAGCUGUAUCAUCC UCUUCUUGGUAGCAACAGCUACAGGCGCGCACU CCAGUAUAAUCAACUUUGAAAAACUGAGCGAGA AGGACGAGCUGUAGAAAAAAAAAAAAAAAAAA AA	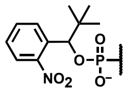	15.87
**ORN7**			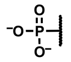	10.37

* G_m: 2’-*O*-Methylguanosine

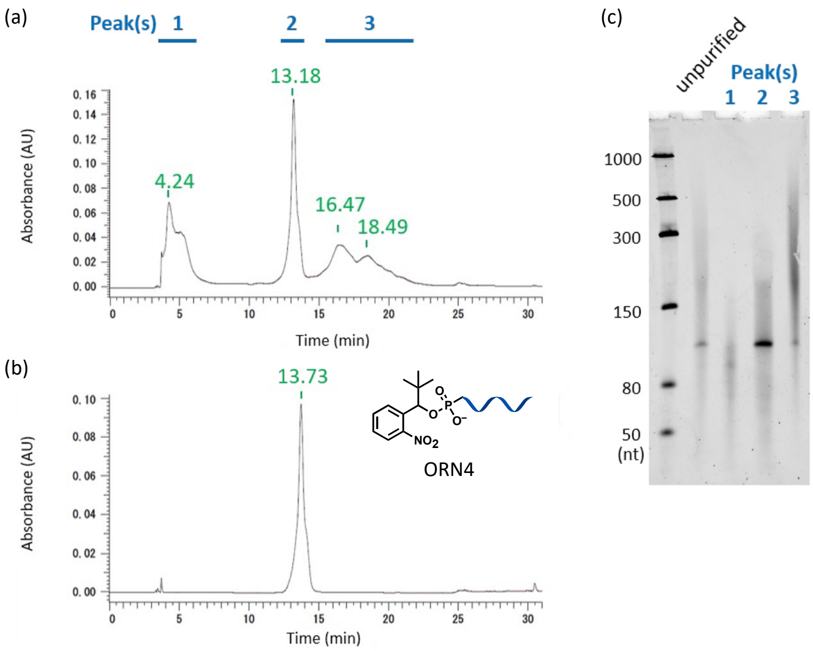

Figure S20. HPLC profiles and dPAGE image for **ORN4** synthesis. (a) HPLC profile of the crude **ORN3** (HPLC condition, Column: YMC-Triart bio C4 (250 x 4.6 mmI.D., S-5 μm, 30 nm), Solvent A: 50 mM TEAA buffer (pH 7.0) + 5% CH_3CN, Solvent B: CH_3CN, Gradient: 5-20%B over 20 minutes, column temperature 50 °C, Flow rate: 1 mL/min, detection: 260 nm, loop size: 2.0 mL), (b) HPLC profile of **ORN4** after RP-HPLC purification (HPLC condition, Column: YMC-Triart bio C4 (250 x 4.6 mmI.D., S-5 μm, 30 nm), Solvent A: 50 mM TEAA buffer (pH 7.0) + 5% CH_3CN, Solvent B: CH_3CN, Gradient: 5-20%B over 20 minutes, column temperature 50 °C, Flow rate: 1 mL/min, detection: 260 nm, loop size: 2.0 mL), (c) dPAGE image of peak 1–3 of crude HPLC profile (5% dPAGE containing 7M urea).

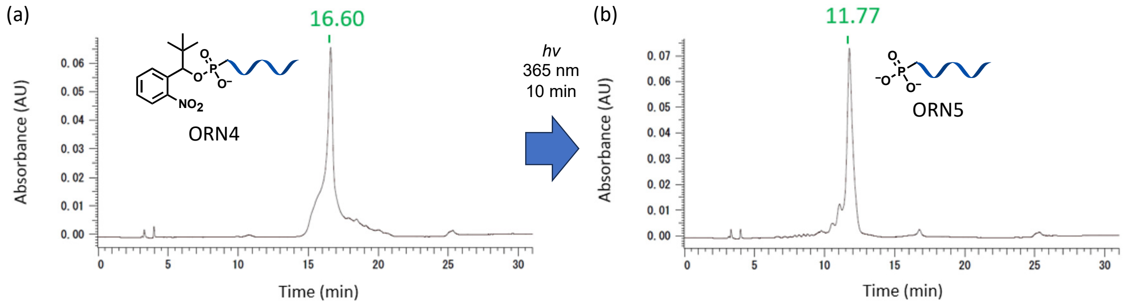

Figure S21. HPLC profiles for UV-induced deprotection of **ORN4**. (a) HPLC profiles of **ORN4** and (b) produced **ORN5** by UV-irradiation of **ORN4** (HPLC condition, Column: Hydrosphere C18 (250 x 4.6 mmI.D., S-5 µm, 12 nm), Solvent A: 50 mM TEAA buffer (pH 7.0) + 5% CH_3CN, Solvent B: CH_3CN, Gradient: 0-20%B over 20 minutes, column temperature 50 °C, Flow rate: 1 mL/min, detection: 260 nm, loop size: 2.0 mL).

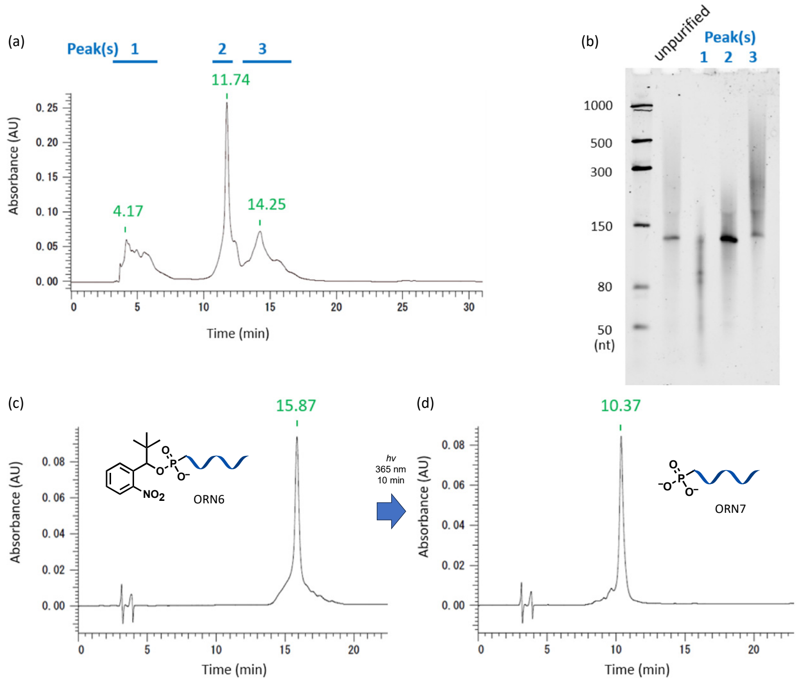

Figure S22. HPLC profiles and dPAGE image for **ORN6** synthesis and UV-induced deprotection. (a) HPLC profile of the crude **ORN6** (HPLC condition, Column: YMC-Triart bio C4 (250 x 4.6 mmI.D., S-5 µm, 30 nm), Solvent A: 50 mM TEAA buffer (pH 7.0) + 5% CH_3CN, Solvent B: CH_3CN, Gradient: 5-20%B over 20 minutes, column temperature 50 °C, Flow rate: 1 mL/min, detection: 260 nm, loop size: 2.0 mL), (b) dPAGE image of peak 1–3 of crude HPLC profile (5% dPAGE containing 7M urea), (c) HPLC profile of **ORN6** after RP-HPLC purification (HPLC condition, Column: Hydrosphere C18 (250 x 4.6 mmI.D., S-5 µm, 12 nm), Solvent A: 50 mM TEAA buffer (pH 7.0) + 5% CH_3CN, Solvent B: CH_3CN, Gradient: 5-15%B over 20 minutes, column temperature 50 °C, Flow rate: 1 mL/min, detection: 260 nm, loop size: 2.0 mL), (d) HPLC profile of the produced **ORN7** by UV-irradiation of **ORN6** (HPLC condition, Column: Hydrosphere C18 (250 x 4.6 mmI.D., S-5 µm, 12 nm), Solvent A: 50 mM TEAA buffer (pH 7.0) + 5% CH_3CN, Solvent B: CH_3CN, Gradient: 5-15%B over 20 minutes, column temperature 50 °C, Flow rate: 1 mL/min, detection: 260 nm, loop size: 2.0 mL).

7. Analysis of 5′-phosphate RNAs Which Synthesized by *In Vitro* Transcription

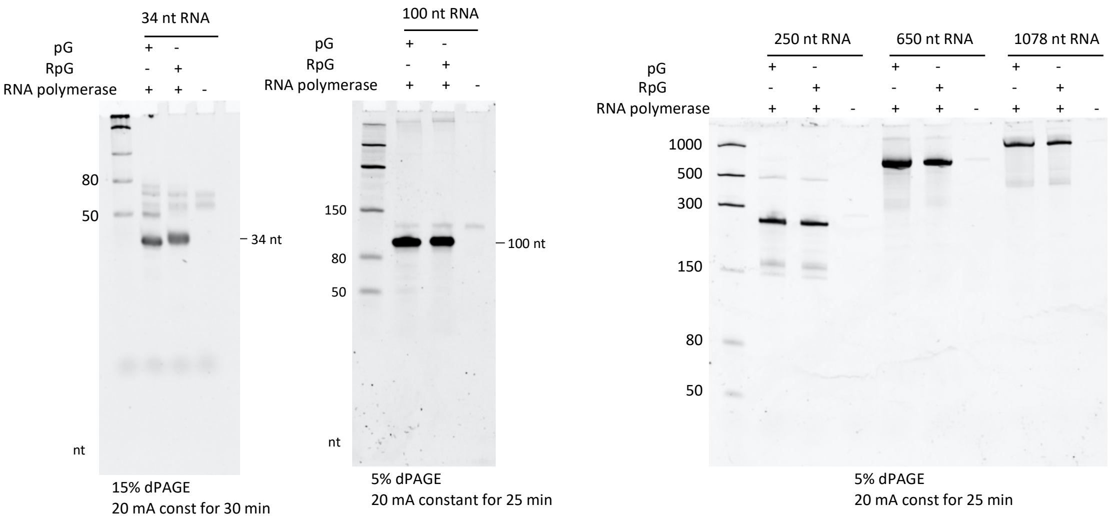

Figure S23. dPAGE analysis of transcribed products.

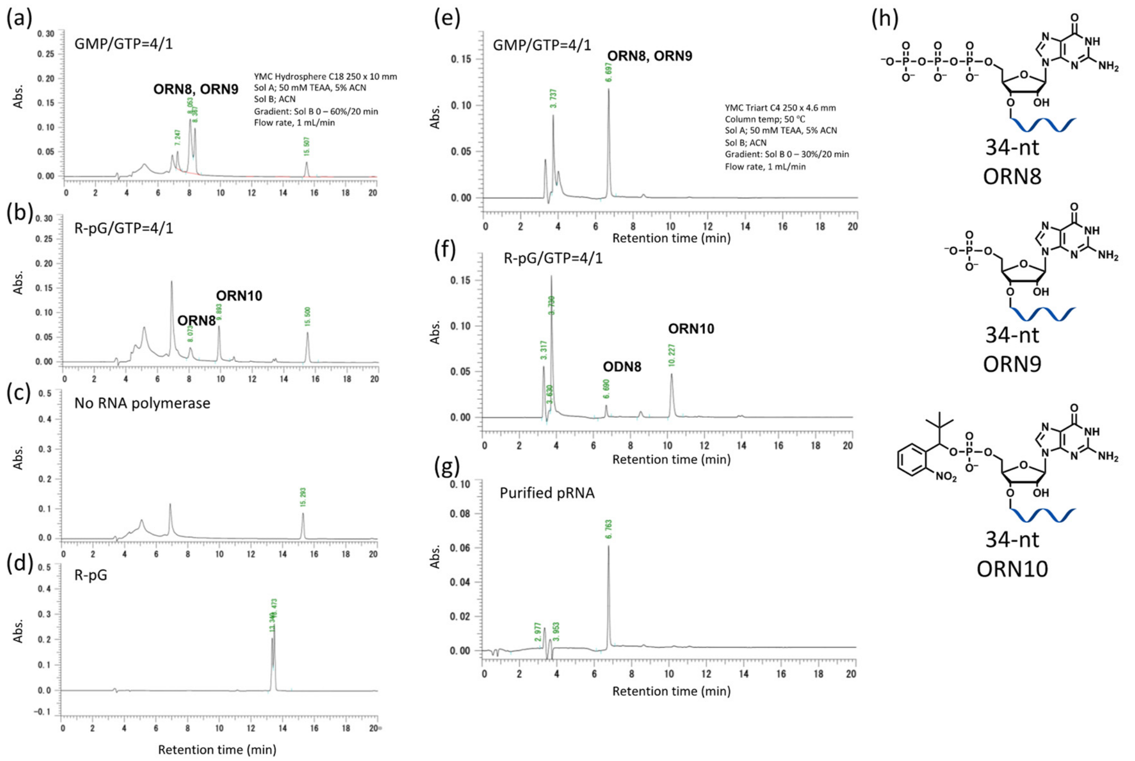

Figure S24. HPLC analysis of *in vitro* transcribed RNAs; (a) *In vitro* transcription product mixture prepared from GMP/GTP = 4/1, (b) *In vitro* transcription product mixture prepared from RpG (**16**)/GTP = 4/1, (c) *In vitro* transcription mixture without addition of RNA polymerase, (d) RpG (**16**) only (HPLC condition for (a)–(d): Column: Hydrosphere C18 (250 x 4.6 mmI.D., S-5 µm, 12 nm), Solvent A: 50 mM TEAA buffer (pH 7.0) + 5% CH_3CN, Solvent B: CH_3CN, Gradient: 0-60%B over 20 minutes, column temperature 50 °C, Flow rate: 1 mL/min, detection: 260 nm, loop size: 2.0 mL), (e) *In vitro* transcription product mixture prepared from GMP/GTP = 4/1, (f) *In vitro* transcription

product mixture prepared from RpG (**16**)/GTP = 4/1, (g) 34-nt **ORN9** (HPLC condition for (e)–(g): Column: YMC-Triart bio C4 (250 x 4.6 mmI.D., S-5 µm, 30 nm), Solvent A: 50 mM TEAA buffer (pH 7.0) + 5% CH_3CN, Solvent B: CH_3CN, Gradient: 0-30%B over 20 minutes, column temperature 50 °C, Flow rate: 1 mL/min, detection: 260 nm, loop size: 2.0 mL), (h) Structure of 34-nt **ORN8–ORN10**.

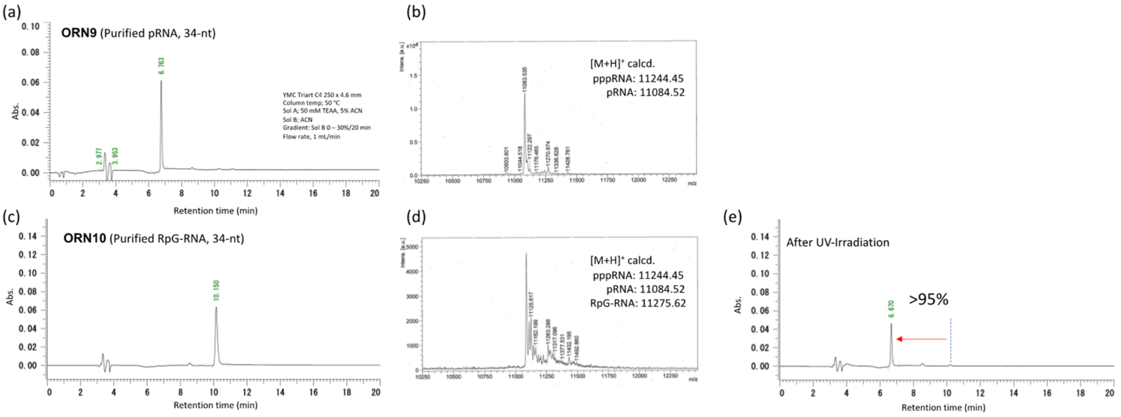

Figure S25. HPLC profiles (HPLC condition: Column: YMC-Triart bio C4 (250 x 4.6 mmI.D., S-5 µm, 30 nm), Solvent A: 50 mM TEAA buffer (pH 7.0) + 5% CH_3CN, Solvent B: CH_3CN, Gradient: 0-30%B over 20 minutes, column temperature 50 °C, Flow rate: 1 mL/min, detection: 260 nm, loop size: 2.0 mL)) and MALDI-TOF-MS spectra (Linear Positive Mode, Matrix: 3-HPA) of 34-nt *in vitro* transcribed RNAs; (a) HPLC profile of 34-nt **ORN9**, (b) MALDI-TOF-MS spectrum of 34-nt **ORN9**, (c) HPLC profile of 34-nt **ORN10**, (d) HPLC profile of 34-nt **ORN10**, (e) HPLC profile of **ORN9** which produced by UV-irradiation of **ORN10**.

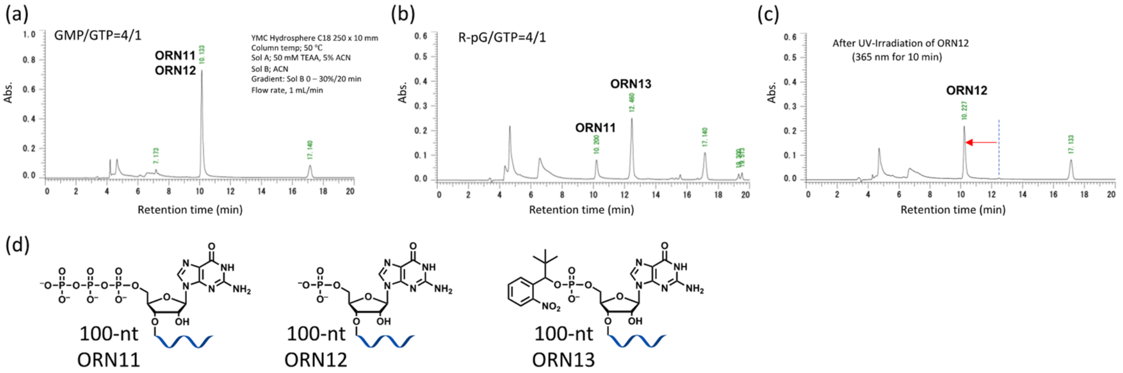

Figure S26. HPLC profiles (HPLC condition: Column: Hydrosphere C18 (250 x 4.6 mmI.D., S-5 µm, 12 nm), Solvent A: 50 mM TEAA buffer (pH 7.0) + 5% CH_3CN, Solvent B: CH_3CN, Gradient: 0-30%B over 20 minutes, column temperature 50 °C, Flow rate: 1 mL/min, detection: 260 nm, loop size: 2.0 mL)) of 100-nt *in vitro* transcribed RNAs; (a) *In vitro* transcription product mixture prepared from GMP/GTP = 4/1, (b) *In vitro* transcription product mixture prepared from RpG (**16**)/GTP = 4/1, (c) UV-irradiation product of 100-nt **ORN13** to produce **ORN12**, (d) Structure of 100-nt **ORN11–ORN13**.

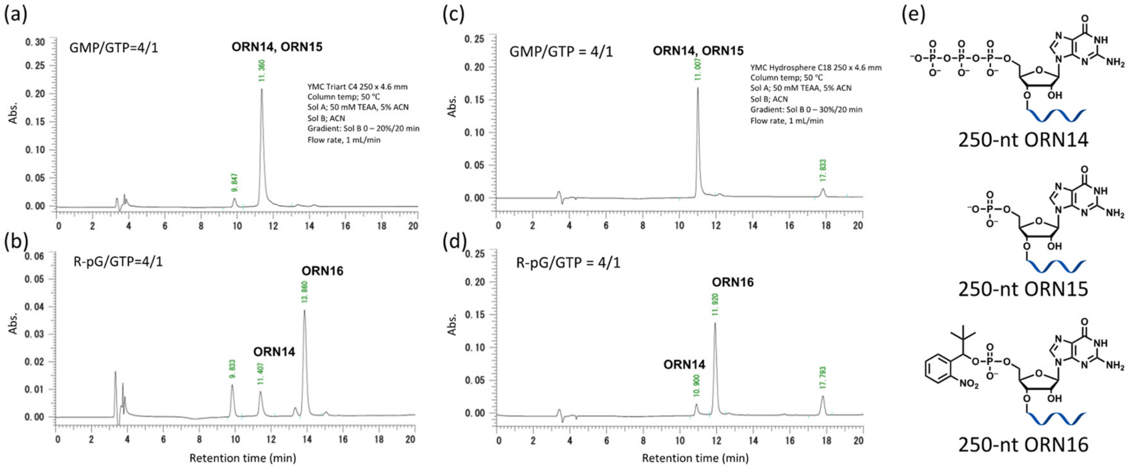

Figure S27. HPLC profiles of 250-nt *in vitro* transcribed RNAs: (a) *In vitro* transcription product mixture prepared from GMP/GTP = 4/1, (b) *In vitro* transcription product mixture prepared from RpG (**16**)/GTP = 4/1 (HPLC condition for (a) and (b): Column: YMC-Triart bio C4 (250 x 4.6 mmI.D., S-5 μm, 30 nm), Solvent A: 50 mM TEAA buffer (pH 7.0) + 5% CH_3CN, Solvent B: CH_3CN, Gradient: 0-20%B over 20 minutes, column temperature 50 °C, Flow rate: 1 mL/min, detection: 260 nm, loop size: 2.0 mL), (c) In vitro transcription product mixture prepared from GMP/GTP = 4/1, (d) In vitro transcription product mixture prepared from RpG (**16**)/GTP = 4/1 (HPLC condition for (c) and (d): Column: Hydrosphere C18 (250 x 4.6 mmI.D., S-5 μm, 12 nm), Solvent A: 50 mM TEAA buffer (pH 7.0) + 5% CH_3CN, Solvent B: CH_3CN, Gradient: 0-30%B over 20 minutes, column temperature 50 °C, Flow rate: 1 mL/min, detection: 260 nm, loop size: 2.0 mL), (e) Structure of 250-nt **ORN14–ORN16**.

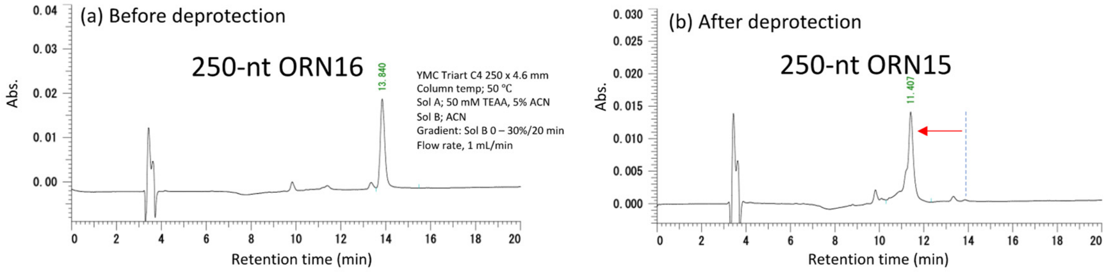

Figure S28. HPLC profiles of (a) 250-nt **ORN16** and (b) 250-nt **ORN15** which produced by UV-irradiation of **ORN16** (HPLC condition: Column: YMC-Triart bio C4 (250 x 4.6 mmI.D., S-5 μm, 30 nm), Solvent A: 50 mM TEAA buffer (pH 7.0) + 5% CH_3CN, Solvent B: CH_3CN, Gradient: 0-30%B over 20 minutes, column temperature 50 °C, Flow rate: 1 mL/min, detection: 260 nm, loop size: 2.0 mL).

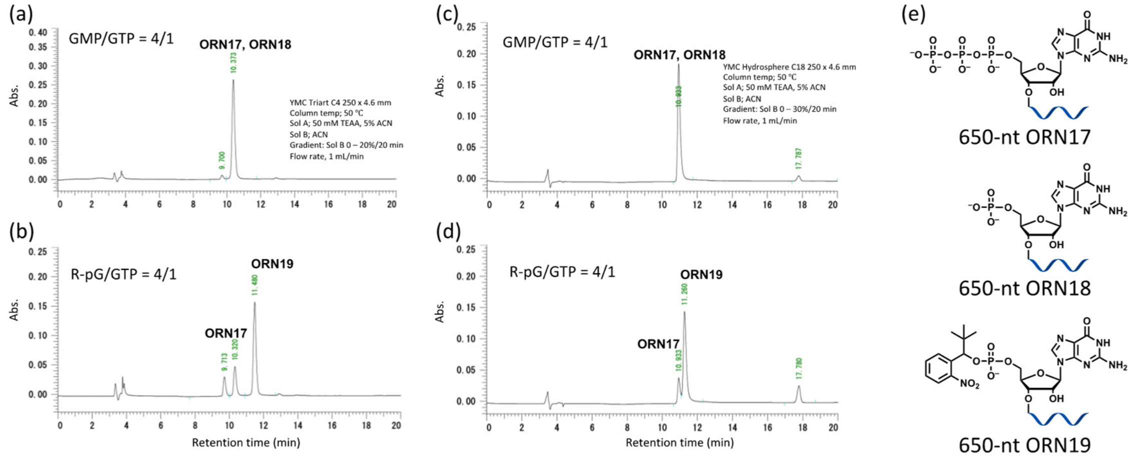

Figure S29. HPLC profiles of 650-nt *in vitro* transcribed RNAs: (a) *In vitro* transcription product mixture prepared from GMP/GTP = 4/1, (b) *In vitro* transcription product mixture prepared from RpG (**16**)/GTP = 4/1 (HPLC condition for (a) and (b): Column: YMC-Triart bio C4 (250 x 4.6 mmI.D., S-5 μm, 30 nm), Solvent A: 50 mM TEAA buffer (pH 7.0) + 5% CH_3CN, Solvent B: CH_3CN, Gradient: 0-20%B over 20 minutes, column temperature 50 °C, Flow rate: 1 mL/min, detection: 260 nm, loop size: 2.0 mL), (c) *In vitro* transcription product mixture prepared from GMP/GTP = 4/1, (d) *In vitro* transcription product mixture prepared from RpG (**16**)/GTP = 4/1 (HPLC condition for (c) and (d): Column: Hydrosphere C18 (250 x 4.6 mmI.D., S-5 μm, 12 nm), Solvent A: 50 mM TEAA buffer (pH 7.0) + 5% CH_3CN, Solvent B: CH_3CN, Gradient: 0-30%B over 20 minutes, column temperature 50 °C, Flow rate: 1 mL/min, detection: 260 nm, loop size: 2.0 mL), (e) Structure of 650-nt **ORN17–ORN19**.

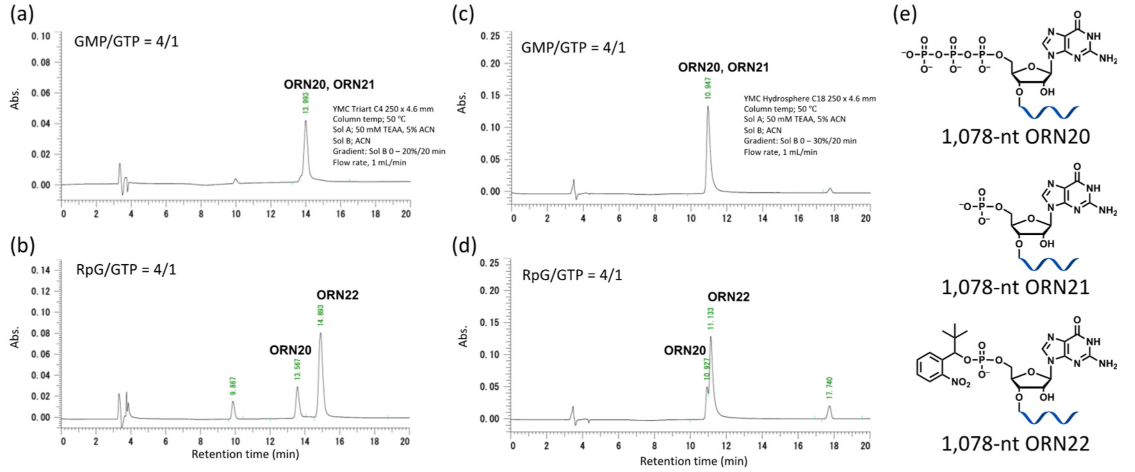

Figure S30. HPLC profiles of 1,078-nt *in vitro* transcribed RNAs: (a) *In vitro* transcription product mixture prepared from GMP/GTP = 4/1, (b) *In vitro* transcription product mixture prepared from RpG (**16**)/GTP = 4/1 (HPLC condition for (a) and (b): Column: YMC-Triart bio C4 (250 x 4.6 mmI.D., S-5 μm, 30 nm), Solvent A: 50 mM TEAA buffer (pH 7.0) + 5% CH_3CN, Solvent B: CH_3CN, Gradient: 0-20%B over 20 minutes, column temperature 50 °C, Flow rate: 1 mL/min, detection: 260 nm, loop size: 2.0 mL), (c) *In vitro* transcription product mixture prepared from GMP/GTP = 4/1, (d) *In vitro* transcription product mixture prepared from RpG (**16**)/GTP = 4/1 (HPLC condition for (c) and (d): Column: Hydrosphere C18 (250 x 4.6 mmI.D., S-5 μm, 12 nm), Solvent A: 50 mM TEAA buffer (pH 7.0) + 5% CH_3CN, Solvent B: CH_3CN, Gradient: 0-30%B over

20 minutes, column temperature 50 °C, Flow rate: 1 mL/min, detection: 260 nm, loop size: 2.0 mL), (e) Structure of 1,078-nt **ORN20–ORN22**.

Table S4. Result of RP-HPLC analysis (Hydrosphere C18 column) of *In Vitro* Transcribed RNAs Using RpG (16).

Analysis Condition*			pRNA, pppRNA	RpG-RNA		
Gradient	Column Temp.	Length	Retention Time	Retention Time	Yield	Gap
0–60%B	r.t.	34-nt	8.07 min	9.89 min	66.7%	1.82 min
0–30%B	50 °C	100-nt	10.2 min	12.5 min	74.1%	2.26 min
0–30%B	50 °C	250-nt	10.9 min	11.9 min	74.1%	1.02 min
0–30%B	50 °C	650-nt	10.9 min	11.3 min	74.0%	0.33 min
0–30%B	50 °C	1078-nt	10.9 min	11.1 min	68.1%	0.21 min

All analysis samples were prepared by in vitro transcription using RpG (16). The HPLC solvent gradient, column temperature, and retention times for the analysis of transcriptionally synthesized RNAs of different lengths were summarized.

*HPLC condition, Column: Hydrosphere C18 (250 x 4.6 mmI.D., S-5 μm, 12 nm), Solvent A: 50 mM TEAA buffer (pH 7.0) + 5% CH_3CN, Solvent B: CH_3CN, Gradient over 20 minutes, Flow rate: 1 mL/min, detection: 260 nm, loop size: 2.0 mL

Table S5. Result of RP-HPLC analysis (YMC Triart Bio C4 column) of *In Vitro* Transcribed RNAs Using RpG (16).

Analysis Condition*			pRNA, pppRNA	RpG-RNA		
Gradient	Column Temp.	Length	Retention Time	Retention Time	Yield	Gap
0–30%B	50 °C	34-nt	6.69 min	10.2 min	68.8%	3.54 min
0–20%B	50 °C	250-nt	11.4 min	13.9 min	76.9%	2.45 min
0–20%B	50 °C	650-nt	10.3 min	11.5 min	71.1%	1.16 min
0–20%B	50 °C	1078-nt	14.0 min	15.2 min	70.0%	1.25 min

All analysis samples were prepared by in vitro transcription using RpG (16). The HPLC solvent gradient, column temperature, and retention times for the analysis of transcriptionally synthesized RNAs of different lengths were summarized.

*HPLC condition, Column: YMC-Triart bio C4 (250 x 4.6 mmI.D., S-5 μm, 30 nm), Solvent A: 50 mM TEAA buffer (pH 7.0) + 5% CH_3CN, Solvent B: CH_3CN, Gradient over 20 minutes, Flow rate: 1 mL/min, detection: 260 nm, loop size: 2.0 mL

Table S6. Template DNA Sequences for Synthesizing 5’ -Monophosphate RNA by IVT.

RNA Length	IVT Template DNA Sequence*
34-nt	CCCGGATCCTAATACGACTCACTATAGGGATCCGAAGGAGATATATCCGATGGACTACAA
100-nt	CCCGGATCCTAATACGACTCACTATAGGCGCATATTAAGGTGACGCGTGTGGCCTCGAACACCG<br>AGCGACCCTGCAGCGACCCGCTTAAAAGCTTGGCAATCCGGTACTGTTGGTAAAGCCACCAT
250-nt	CCCGGATCCTAATACGACTCACTATAGGACGACGACGACAAGATCATCGACTATAAAGACGACG<br>ACGATAAAGGTGGCGACTATAAGGACGACGACGACAAAGCCATTAATAGTGACTCTGAGTGTC<br>CCCTGTCCCACGACGGGTACTGCCTCCACGACGGTGTGTGCATGTATATTGAAGCATTGGACAA<br>GTACGCCTGCAACTGTGTTGTTGGCTACATCGGGGAGCGCTGTCAGTACCGAGACCTGAAGTG<br>GTGGGAACTGCGCCTGCAGTAG
650-nt	CCCGGATCCTAATACGACTCACTATAGGCGCATATTAAGGTGACGCGTGTGGCCTCGAACACCG<br>AGCGACCCTGCAGCGACCCGCTTAAAAGCTTGGCAATCCGGTACTGTTGGTAAAGCCACCATG<br>GTCTTCACACTCGAAGATTTCGTTGGGGACTGGCGACAGACAGCCGGCTACAACCTGGACCAA<br>GTCCTTGAACAGGGAGGTGTGTCCAGTTTGTTTCAGAATCTCGGGGTGTCCGTAACTCCGATC<br>CAAAGGATTGTCCTGAGCGGTGAAAATGGGCTGAAGATCGACATCCATGTCATCATCCCGTAT<br>GAAGGTCTGAGCGGCGACCAAATGGGCCAGATCGAAAAAATTTTAAGGTGGTGTACCCTGTG<br>GATGATCATCACTTTAAGGTGATCCTGCACTATGGCACACTGGTAATCGACGGGGTTACGCCGA<br>ACATGATCGACTATTTCGGACGGCCGTATGAAGGCATCGCCGTGTTCGACGGCAAAAAGATCA<br>CTGTAACAGGGACCCTGTGGAACGGCAACAAAATTATCGACGAGCGCCTGATCAACCCCGACG<br>GCTCCCTGCTGTTCCGAGTAACCATCAACGGAGTGACCGGCTGGCGGCTGTGCGAACGCATTC<br>TGGCGTAATTCTAGAAAAAAAAAAAAAAAAAAAAAAAAAAAA
1078-nt	CAATCTGGATTAATACGACTCACTATAGGGCTAGTAATTCACGTCTGCTCGAAGCGGCCGCTCT<br>AGAACTAACCTTACGCCACCATGACTTCGAAAGTTTATGATCCAGAACAAAGGAAACGGATGA<br>TAACTGGTCCGCAGTGGTGGGCCAGATGTAAACAAATGAATGTTCTTGATTCATTTATTAATTA<br>TTATGATTCAGAAAAACATGCAGAAAATGCTGTTATTTTTTTACATGGTAACGCGGCCTCTTC<br>TTATTTATGGCGACATGTTGTGCCACATATTGAGCCAGTAGCGCGGTGTATTATACCAGACCTT<br>ATTGGTATGGGCAAATCAGGCAAATCTGGTAATGGTTCTTATAGGTTACTTGATCATTACAAAT<br>ATCTTACTGCATGGTTTGAACTTCTTAATTTACCAAAGAAGATCATTTTTGTCGGCCATGATTG<br>GGGTGCTTGTTTGGCATTTCATTATAGCTATGAGCATCAAGATAAGATCAAAGCAATAGTTCAC<br>GCTGAAAGTGTAGTAGATGTGATTGAATCATGGGATGAATGGCCTGATATTGAAGAAGATATT<br>GCGTTGATCAAATCTGAAGAAGGAGAAAAAATGGTTTTGGAGAATAACTTCTTCGTGGAAAC<br>CATGTTGCCATCAAAAATCATGAGAAAGTTAGAACCAGAAGAATTTGCAGCATATCTTGAACC<br>ATTCAAAGAGAAAGGTGAAGTTCGTCGTCCAACATTATCATGGCCTCGTGAAATCCCGTTAGT<br>AAAAGGTGGTAAACCTGACGTTGTACAAATTGTTAGGAATTATAATGCTTATCTACGTGCAAG<br>TGATGATTTACCAAAAATGTTTATTGAATCGGACCCAGGATTCTTTTCCAATGCTATTGTTGA<br>AGGTGCCAAGAAGTTTCCTAATACTGAATTTGTCAAAGTAAAAGGTCTTCATTTTTCGCAAGA<br>AGATGCACCTGATGAAATGGGAAAATATATCAAATCGTTCGTTGAGCGAGTTCTCAAAAATGA<br>ACAATAACGTACTCAAAGTATAAGTCAACACAACTATGTCAGAAGCAAATGTAATTTCACATAA<br>AAAAAAAAAAAAAAAAAAAAAAAAAAA

*The yellow-highlighted 18-base sequence is the T7 promotor, which is transcription start site recognized by T7 RNA polymerase.

8. dPAGE Analysis for Synthesis of Circular mRNA

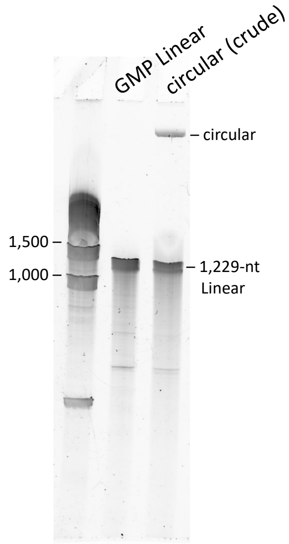

Figure S31. 5% dPAGE analysis of the linear IRES-mRNA that prepared by *in vitro* transcription using GMP as a substrate and the crude IRES circular mRNA (1 x TBE buffer, gel size: 8 cm x 8 cm, 26 mA, 120 mins, Ethidium bromide stain). The circularization efficiency was calculated to 54.0% from the ratio of the band intensity of linear and circular RNAs. Gel purification was performed to obtain circular mRNA and used for translation experiments.

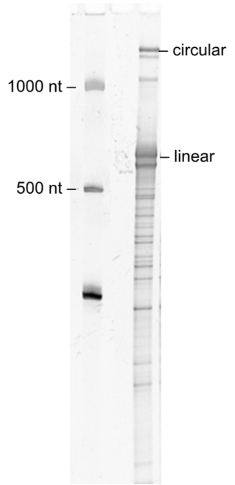

Figure S32. 5% dPAGE analysis of the crude no IRES circular mRNA that prepared by *in vitro* transcription using *o*-RpG (**16**) as a substrate (1 x TBE buffer, gel size: 8 cm x 8 cm, 26 mA, 120 mins, Ethidium bromide stain). The circularization efficiency was calculated to 35.1% from the ratio of the band intensity of linear and circular RNAs. Gel purification was performed to obtain circular mRNA and used for translation experiments.

9. Comparison of *In Vitro* Translational Activity of Circular and Linear mRNAs

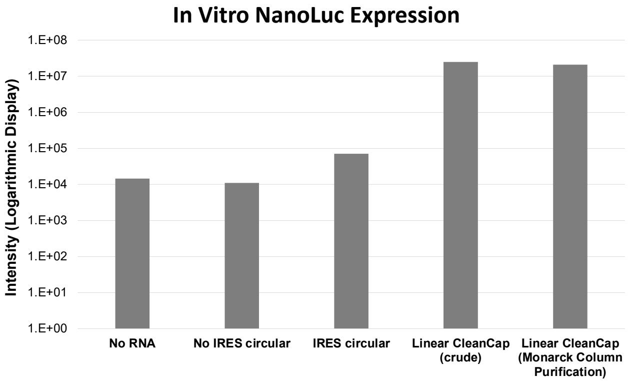

Figure S33. *In vitro* NanoLuc expression levels of no IRES circular mRNA, IRES circular mRNA, linear CleanCap-AG mRNA (with or without Monarck RNA Cleanup column purification) using HeLa cells (seeding 1.0×10^4 /100 µL 96-well plate, Lipofectamine® Messenger MAX Reagent, 10 ng mRNA, 0.15 µL Lipofectamine/ 5 µL Opti-MEM. After 24 hours incubation at 37 °C, Nano-Glo® luciferase assay (Promega N1110) was performed.

10. DNA/RNA Sequences for *In Vitro* RNA Synthesis and Circular mRNA Synthesis

Primer DNAs for PCR

Forward primer: 5’-CCCGGATCCTAATACGACTCACTATAGGCGCATATTAAGGTGACGCGT-3’

Reverse primer: 5’-TTTTTTTTTTTTTTTTTTTTTTTTTTTTTTTCTAGAATTACGCCAGAATGCG-3’

* Underlined capitals indicate hybridization region with template DNA.

pNL1.1TK (GenBank: KM359774.1)

GGCCTAACTGGCCGGTACCTGAGTCTAAATGAGTCTTCGGACCTCGCGGGGGCCGCTTAAGCGGTGGTT
AGGGTTTGTCTGACGCGGGGGGAGGGGGAAGGAACGAAACACTCTCATTCGGAGGCGGCTCGGGGTTT
GGTCTTGGTGGCCACGGGCACGCAGAAGAGCGCCGCGATCCTCTTAAGCACCCCCCCGCCCTCCGTGGA
GGCGGGGGTTTGGTCGGCGGGTGGTAACTGGCGGGCCGCTGACTCGGGCGGGTCGCGCGCCCCAGAGT
GTGACCTTTTCGGTCTGCTCGCAGACCCCCGGGCGGCGCCGCCGCGGCGGCGACGGGCTCGCTGGGTCC
TAGGCTCCATGGGGACCGTATACGTGGACAGGCTCTGGAGCATCCGCACGACTGCGGTGATATTACCGG
AGACCTTCTGCGGGACGAGCCGGGTCACGCGGCTGACGCGGAGCGTCCGTTGGGCGACAAACACCAGG
ACGGGGCACAGGTACACTATCTTGTCACCCGGAGGCGCGAGGGACTGCAGGAGCTTCAGGGAGTGGCG
CAGCTGCTTCATCCCCGTGGCCCGTTGCTCGCGTTTGCTGGCGGTGTCCCCGGAAGAAATATATTTGCA
TGTCTTTAGTTCTATGATGACACAAACCCCGCCCAGCGTCTTGTCATTGGCGAATTCGAACACGCAGAT
GCAGTCGGGGCGGCGCGGTCCCAGGTCCACTTCGCATATTAAGGTGACGCGTGTGGCCTCGAACACCGA
GCGACCCTGCAGCGACCCGCTTAAAAGCTTGGCAATCCGGTACTGTTGGTAAAGCCACCATGGTCTTCA
CACTCGAAGATTTCGTTGGGGACTGGCGACAGACAGCCGGCTACAACCTGGACCAAGTCCTTGAACAG
GGAGGTGTGTCCAGTTTGTTTCAGAATCTCGGGGTGTCCGTAACTCCGATCCAAAGGATTGTCCTGAGC
GGTGAAAATGGGCTGAAGATCGACATCCATGTCATCATCCCGTATGAAGGTCTGAGCGGCGACCAAATG
GGCCAGATCGAAAAAATTTTTAAGGTGGTGTACCCTGTGGATGATCATCACTTTAAGGTGATCCTGCAC
TATGGCACACTGGTAATCGACGGGGTTACGCCGAACATGATCGACTATTTCGGACGGCCGTATGAAGGC
ATCGCCGTGTTCGACGGCAAAAAGATCACTGTAACAGGGACCCTGTGGAACGGCAACAAAATTATCGA
CGAGCGCCTGATCAACCCCGACGGCTCCCTGCTGTTCCGAGTAACCATCAACGGAGTGACCGGCTGGCG
GCTGTGCGAACGCATTCTGGCGTAATTCTAGAGTCGGGGCGGCCGGCCGCTTCGAGCAGACATGATAAG
ATACATTGATGAGTTTGGACAAACCACAACTAGAATGCAGTGAAAAAAATGCTTTATTTGTGAAATTT

GTGATGCTATTGCTTTATTTGTAACCATTATAAGCTGCAATAAACAAGTTAACAACAACAATTGCATTC
ATTTTATGTTTCAGGTTCAGGGGGAGGTGTGGGAGGTTTTTTAAAGCAAGTAAAACCTCTACAAATGT
GGTAAAATCGATAAGGATCCGTCGACCGATGCCCTTGAGAGCCTTCAACCCAGTCAGCTCCTTCCGGTG
GGCGCGGGGCATGACTATCGTCGCCGCACTTATGACTGTCTTCTTTATCATGCAACTCGTAGGACAGGT
GCCGGCAGCGCTCTTCCGCTTCCTCGCTCACTGACTCGCTGCGCTCGGTCGTTCGGCTGCGGCGAGCGG
TATCAGCTCACTCAAAGGCGGTAATACGGTTATCCACAGAATCAGGGGATAACGCAGGAAAGAACATGT
GAGCAAAAGGCCAGCAAAAGGCCAGGAACCGTAAAAAGGCCGCGTTGCTGGCGTTTTTCCATAGGCTC
CGCCCCCCTGACGAGCATCACAAAAATCGACGCTCAAGTCAGAGGTGGCGAAACCCGACAGGACTATAA
AGATACCAGGCGTTTCCCCCTGGAAGCTCCCTCGTGCGCTCTCCTGTTCCGACCCTGCCGCTTACCGGA
TACCTGTCCGCCTTTCTCCCTTCGGGAAGCGTGGCGCTTTCTCATAGCTCACGCTGTAGGTATCTCAGT
TCGGTGTAGGTCGTTCGCTCCAAGCTGGGCTGTGTGCACGAACCCCCCGTTCAGCCCGACCGCTGCGCC
TTATCCGGTAACTATCGTCTTGAGTCCAACCCGGTAAGACACGACTTATCGCCACTGGCAGCAGCCACT
GGTAACAGGATTAGCAGAGCGAGGTATGTAGGCGGTGCTACAGAGTTCTTGAAGTGGTGGCCTAACTAC
GGCTACACTAGAAGAACAGTATTTGGTATCTGCGCTCTGCTGAAGCCAGTTACCTTCGGAAAAAGAGTT
GGTAGCTCTTGATCCGGCAAACAAACCACCGCTGGTAGCGGTGGTTTTTTTGTTTGCAAGCAGCAGAT
TACGCGCAGAAAAAAAGGATCTCAAGAAGATCCTTTGATCTTTTCTACGGGGTCTGACGCTCAGTGGA
ACGAAAACTCACGTTAAGGGATTTTGGTCATGAGATTATCAAAAAGGATCTTCACCTAGATCCTTTTAA
ATTAAAAATGAAGTTTTAAATCAATCTAAAGTATATATGAGTAAACTTGGTCTGACAGCGGCCGCAAAT
GCTAAACCACTGCAGTGGTTACCAGTGCTTGATCAGTGAGGCACCGATCTCAGCGATCTGCCTATTTCG
TTCGTCCATAGTGGCCTGACTCCCCGTCGTGTAGATCACTACGATTCGTGAGGGCTTACCATCAGGCCCC
AGCGCAGCAATGATGCCGCGAGAGCCGCGTTCACCGGCCCCCGATTTGTCAGCAATGAACCAGCCAGCA
GGGAGGGCCGAGCGAAGAAGTGGTCCTGCTACTTTGTCCGCCTCCATCCAGTCTATGAGCTGCTGTCGT
GATGCTAGAGTAAGAAGTTCGCCAGTGAGTAGTTTCCGAAGAGTTGTGGCCATTGCTACTGGCATCGTG
GTATCACGCTCGTCGTTCGGTATGGCTTCGTTCAACTCTGGTTCCCAGCGGTCAAGCCGGGTCACATGA
TCACCCATATTATGAAGAAATGCAGTCAGCTCCTTAGGGCCTCCGATCGTTGTCAGAAGTAAGTTGGCC
GCGGTGTTGTCGCTCATGGTAATGGCAGCACTACACAATTCTCTTACCGTCATGCCATCCGTAAGATGC
TTTTCCGTGACCGGCGAGTACTCAACCAAGTCGTTTTGTGAGTAGTGTATACGGCGACCAAGCTGCTCT
TGCCCGGCGTCTATACGGGACAACACCGCGCCACATAGCAGTACTTTGAAAGTGCTCATCATCGGGAAT
CGTTCTTCGGGGCGGAAAGACTCAAGGATCTTGCCGCTATTGAGATCCAGTTCGATATAGCCCACTCTT
GCACCCAGTTGATCTTCAGCATCTTTTACTTTCACCAGCGTTTCGGGGTGTGCAAAAACAGGCAAGCA
AAATGCCGCAAAGAAGGGAATGAGTGCGACACGAAAATGTTGGATGCTCATACTCGTCCTTTTTCAAT
ATTATTGAAGCATTTATCAGGGTTACTAGTACGTCTCTCAAGGATAAGTAAGTAATATTAAGGTACGGG
AGGTATTGGACAGGCCGCAATAAAATATCTTTATTTTCATTACATCTGTGTGTTGGTTTTTTGTGTGAA
TCGATAGTACTAACATACGCTCTCCATCAAAACAAAACGAAACAAAACAAACTAGCAAAATAGGCTGTC
CCCAGTGCAAGTGCAGGTGCCAGAACATTTCTCT

* Underlined capitals indicate hybridization region with PCR primers.

* The NanoLuc-coding region is highlighted in orange capitals.

PCR Product DNA (NanoLuc)

CCCGGATCCTAATACGACTCACTATAGGCGCATATTAAGGTGACGCGTGTGGCCTCGAACACCGAGCGA
CCCTGCAGCGACCCGCTTAAAAGCTTGGCAATCCGGTACTGTTGGTAAAGCCACCATGGTCTTCACACT
CGAAGATTTCGTTGGGGACTGGCGACAGACAGCCGGCTACAACCTGGACCAAGTCCTTGAACAGGGAG
GTGTGTCCAGTTTGTTTCAGAATCTCGGGGTGTCCGTAACTCCGATCCAAAGGATTGTCCTGAGCGGTG
AAAATGGGCTGAAGATCGACATCCATGTCATCATCCCGTATGAAGGTCTGAGCGGCGACCAAATGGGCC
AGATCGAAAAAATTTTTAAGGTGGTGTACCCTGTGGATGATCATCACTTTAAGGTGATCCTGCACTATG
GCACACTGGTAATCGACGGGGTTACGCCGAACATGATCGACTATTTCGGACGGCCGTATGAAGGCATCG

CCGTGTTCGACGGCAAAAAGATCACTGTAACAGGGACCCTGTGGAACGGCAACAAAATTATCGACGAG
CGCCTGATCAACCCCGACGGCTCCCTGCTGTTCCGAGTAACCATCAACGGAGTGACCGGCTGGCGGCTG
TGCGAACGCATTCTGGCGTAATTCTAGAAAAAAAAAAAAAAAAAAAAAAAAAAAAAA

* The NanoLuc-coding region is highlighted in orange capitals.

IVT Product NanoLuc-mRNA

GGCGCAUAUUAAGGUGACGCGUGUGGCCUCGAACACCGAGCGACCCUGCAGCGACCCGCUUAAAAGC
UUGGCAAUCCGGUACUGUUGGUAAAGCCACCAUGGUCUUCACACUCGAAGAUUUCGUUGGGGACUG
GCGACAGACAGCCGGCUACAACCUGGACCAAGUCCUUGAACAGGGAGGUGUGUCCAGUUUGUUUCAG
AAUCUCGGGGUGUCCGUAACUCCGAUCCAAAGGAUUGUCCUGAGCGGUGAAAAUGGGCUGAAGAUC
GACAUCCAUGUCAUCAUCCCGUAUGAAGGUCUGAGCGGCGACCAAAUGGGCCAGAUCGAAAAAAUU
UUUAAGGUGGUGUACCCUGUGGAUGAUCAUCACUUUAAGGUGAUCCUGCACUAUGGCACACUGGUA
AUCGACGGGGUUACGCCGAACAUGAUCGACUAUUUCGGACGGCCGUAUGAAGGCAUCGCCGUGUUCG
ACGGCAAAAAGAUCACUGUAACAGGGACCCUGUGGAACGGCAACAAAAUUAUCGACGAGCGCCUGAU
CAACCCCGACGGCUCCCUGCUGUUCCGAGUAACCAUCAACGGAGUGACCGGCUGGCGGCUGUGCGAA
CGCAUUCUGGCGUAAUUCUAGAAAAAAAAAAAAAAAAAAAAAAAAAAAAAA

* The NanoLuc-coding region is highlighted in orange capitals.

EMCV-IRES-pNL1.1TK (IRES + NanoLuc)

GGCCTAACTGGCCGGTACCTGAGTCTAAATGAGTCTTCGGACCTCGCGGGGGCCGCTTAAGCGGTGGTT
AGGGTTTGTCTGACGCGGGGGGAGGGGGAAGGAACGAAACACTCTCATTCGGAGGCGGCTCGGGGTTT
GGTCTTGGTGGCCACGGGCACGCAGAAGAGCGCCGCGATCCTCTTAAGCACCCCCCCGCCCTCCGTGGA
GGCGGGGGTTTGGTCGGCGGGTGGTAACTGGCGGGCCGCTGACTCGGGCGGGTCGCGCGCCCCAGAGT
GTGACCTTTTCGGTCTGCTCGCAGACCCCCGGGCGGCGCCGCCGCGGCGGCGACGGGCTCGCTGGGTCC
TAGGCTCCATGGGGACCGTATACGTGGACAGGCTCTGGAGCATCCGCACGACTGCGGTGATATTACCGG
AGACCTTCTGCGGGACGAGCCGGGTCACGCGGCTGACGCGGAGCGTCCGTTGGGCGACAAACACCAGG
ACGGGGCACAGGTACACTATCTTGTCACCCGGAGGCGCGAGGGACTGCAGGAGCTTCAGGGAGTGGCG
CAGCTGCTTCATCCCCGTGGCCCGTTGCTCGCGTTTGCTGGCGGTGTCCCCGGAAGAAATATATTTGCA
TGTCTTTAGTTCTATGATGACACAAACCCCGCCCAGCGTCTTGTCATTGGCGAATTCGAACACGCAGAT
GCAGTCGGGGCGGCGCGGTCCCAGGTCCACTTCGCATATTAAGGTGACGCGTAATTCACGCGTCGAGCA
TGCATCTAGGGCGGCCAATTCCGCCCCTCTCCCCCCCCCCCCTCTCCCTCCCCCCCCCCTAACGTTACTG
GCCGAAGCCGCTTGGAATAAGGCCGGTGTGCGTTTGTCTATATGTTATTTTCCACCATATTGCCGTCTT
TTGGCAATGTGAGGGCCCGGAAACCTGGCCCTGTCTTCTTGACGAGCATTCCTAGGGGTCTTTCCCCTC
TCGCCAAAGGAATGCAAGGTCTGTTGAATGTCGTGAAGGAAGCAGTTCCTCTGGAAGCTTCTTGAAGA
CAAACAACGTCTGTAGCGACCCTTTGCAGGCAGCGGAACCCCCCACCTGGCGACAGGTGCCTCTGCGGC
CAAAAGCCACGTGTATAAGATACACCTGCAAAGGCGGCACAACCCCAGTGCCACGTTGTGAGTTGGATA
GTTGTGGAAAGAGTCAAATGGCTCTCCTCAAGCGTATTCAACAAGGGGCTGAAGGATGCCCAGAAGGT
ACCCCATTGTATGGGATCTGATCTGGGGCCTCGGTGCACATGCTTTACATGTGTTTAGTCGAGGTTAAA
AAAACGTCTAGGCCCCCCGAACCACGGGGACGTGGTTTTCCTTTGAAAAACACGATGATAAGCTTGCC
ACAACCCGGGATCGCCACCATGGTCTTCACACTCGAAGATTTCGTTGGGGACTGGCGACAGACAGCCG
GCTACAACCTGGACCAAGTCCTTGAACAGGGAGGTGTGTCCAGTTTGTTTCAGAATCTCGGGGTGTCC
GTAACTCCGATCCAAAGGATTGTCCTGAGCGGTGAAAATGGGCTGAAGATCGACATCCATGTCATCATC
CCGTATGAAGGTCTGAGCGGCGACCAAATGGGCCAGATCGAAAAAATTTTTAAGGTGGTGTACCCTGT
GGATGATCATCACTTTAAGGTGATCCTGCACTATGGCACACTGGTAATCGACGGGGTTACGCCGAACAT
GATCGACTATTTCGGACGGCCGTATGAAGGCATCGCCGTGTTCGACGGCAAAAAGATCACTGTAACAGG
GACCCTGTGGAACGGCAACAAAATTATCGACGAGCGCCTGATCAACCCCGACGGCTCCCTGCTGTTCCG

AGTAACCATCAACGGAGTGACCGGCTGGCGGCTGTGCGAACGCATTCTGGCGTAATTCTAGAGTCGGGG
CGGCCGGCCGCTTCGAGCAGACATGATAAGATACATTGATGAGTTTGGACAAACCACAACTAGAATGCA
GTGAAAAAAATGCTTTATTTGTGAAATTTGTGATGCTATTGCTTTATTTGTAACCATTATAAGCTGCAA
TAAACAAGTTAACAACAACAATTGCATTCATTTTATGTTTCAGGTTCAGGGGGAGGTGTGGGAGGTTT
TTTAAAGCAAGTAAAACCTCTACAAATGTGGTAAAATCGATAAGGATCCGTCGACCGATGCCCTTGAGA
GCCTTCAACCCAGTCAGCTCCTTCCGGTGGGCGCGGGGCATGACTATCGTCGCCGCACTTATGACTGTC
TTCTTTATCATGCAACTCGTAGGACAGGTGCCGGCAGCGCTCTTCCGCTTCCTCGCTCACTGACTCGCT
GCGCTCGGTCGTTCGGCTGCGGCGAGCGGTATCAGCTCACTCAAAGGCGGTAATACGGTTATCCACAGA
ATCAGGGGATAACGCAGGAAAGAACATGTGAGCAAAAGGCCAGCAAAAGGCCAGGAACCGTAAAAAG
GCCGCGTTGCTGGCGTTTTTCCATAGGCTCCGCCCCCCTGACGAGCATCACAAAAATCGACGCTCAAGT
CAGAGGTGGCGAAACCCGACAGGACTATAAAGATACCAGGCGTTTCCCCCTGGAAGCTCCCTCGTGCGC
TCTCCTGTTCCGACCCTGCCGCTTACCGGATACCTGTCCGCCTTTCTCCCTTCGGGAAGCGTGGCGCTT
TCTCATAGCTCACGCTGTAGGTATCTCAGTTCGGTGTAGGTCGTTCGCTCCAAGCTGGGCTGTGTGCAC
GAACCCCCCGTTCAGCCCGACCGCTGCGCCTTATCCGGTAACTATCGTCTTGAGTCCAACCCGGTAAGA
CACGACTTATCGCCACTGGCAGCAGCCACTGGTAACAGGATTAGCAGAGCGAGGTATGTAGGCGGTGCT
ACAGAGTTCTTGAAGTGGTGGCCTAACTACGGCTACACTAGAAGAACAGTATTTGGTATCTGCGCTCTG
CTGAAGCCAGTTACCTTCGGAAAAAGAGTTGGTAGCTCTTGATCCGGCAAACAAACCACCGCTGGTAG
CGGTGGTTTTTTTGTTTGCAAGCAGCAGATTACGCGCAGAAAAAAAGGATCTCAAGAAGATCCTTTGA
TCTTTTCTACGGGGTCTGACGCTCAGTGGAACGAAAACTCACGTTAAGGGATTTTGGTCATGAGATTA
TCAAAAAGGATCTTCACCTAGATCCTTTTAAATTAAAAATGAAGTTTTAAATCAATCTAAAGTATATAT
GAGTAAACTTGGTCTGACAGCGGCCGCAAATGCTAAACCACTGCAGTGGTTACCAGTGCTTGATCAGT
GAGGCACCGATCTCAGCGATCTGCCTATTTCGTTCGTCCATAGTGGCCTGACTCCCCGTCGTGTAGATC
ACTACGATTCGTGAGGGCTTACCATCAGGCCCCAGCGCAGCAATGATGCCGCGAGAGCCGCGTTCACCG
GCCCCCGATTTGTCAGCAATGAACCAGCCAGCAGGGAGGGCCGAGCGAAGAAGTGGTCCTGCTACTTT
GTCCGCCTCCATCCAGTCTATGAGCTGCTGTCGTGATGCTAGAGTAAGAAGTTCGCCAGTGAGTAGTTT
CCGAAGAGTTGTGGCCATTGCTACTGGCATCGTGGTATCACGCTCGTCGTTCGGTATGGCTTCGTTCAA
CTCTGGTTCCCAGCGGTCAAGCCGGGTCACATGATCACCCATATTATGAAGAAATGCAGTCAGCTCCTT
AGGGCCTCCGATCGTTGTCAGAAGTAAGTTGGCCGCGGTGTTGTCGCTCATGGTAATGGCAGCACTACA
CAATTCTCTTACCGTCATGCCATCCGTAAGATGCTTTTCCGTGACCGGCGAGTACTCAACCAAGTCGTT
TTGTGAGTAGTGTATACGGCGACCAAGCTGCTCTTGCCCGGCGTCTATACGGGACAACACCGCGCCACA
TAGCAGTACTTTGAAAGTGCTCATCATCGGGAATCGTTCTTCGGGGCGGAAAGACTCAAGGATCTTGC
CGCTATTGAGATCCAGTTCGATATAGCCCACTCTTGCACCCAGTTGATCTTCAGCATCTTTTACTTTCAC
CAGCGTTTCGGGGTGTGCAAAAACAGGCAAGCAAAATGCCGCAAAGAAGGGAATGAGTGCGACACGA
AAATGTTGGATGCTCATACTCGTCCTTTTTCAATATTATTGAAGCATTTATCAGGGTTACTAGTACGTC
TCTCAAGGATAAGTAAGTAATATTAAGGTACGGGAGGTATTGGACAGGCCGCAATAAAATATCTTTATT
TTCATTACATCTGTGTGTTGGTTTTTTGTGTGAATCGATAGTACTAACATACGCTCTCCATCAAAACAA
AACGAAACAAAACAAACTAGCAAAATAGGCTGTCCCCAGTGCAAGTGCAGGTGCCAGAACATTTCTCT

* Underlined capitals indicate hybridization region with PCR primers.
* The IRES region is highlighted in green capitals.
* The NanoLuc-coding region is highlighted in orange capitals.

PCR Product DNA (IRES + NanoLuc)

CCCGGATCCTAATACGACTCACTATAGGCGCATATTAAGGTGACGCGTAATTCACGCGTCGAGCATGCAT
CTAGGGCGGCCAATTCCGCCCCTCTCCCCCCCCCCCCTCTCCCTCCCCCCCCCCTAACGTTACTGGCCGA
AGCCGCTTGGAATAAGGCCGGTGTGCGTTTGTCTATATGTTATTTTCCACCATATTGCCGTCTTTTGGC
AATGTGAGGGCCCGGAAACCTGGCCCTGTCTTCTTGACGAGCATTCCTAGGGGTCTTTCCCCTCTCGCC

AAAGGAATGCAAGGTCTGTTGAATGTCGTGAAGGAAGCAGTTCCTCTGGAAGCTTCTTGAAGACAAAC
AACGTCTGTAGCGACCCTTTGCAGGCAGCGGAACCCCCCACCTGGCGACAGGTGCCTCTGCGGCCAAAA
GCCACGTGTATAAGATACACCTGCAAAGGCGGCACAACCCCAGTGCCACGTTGTGAGTTGGATAGTTGT
GGAAAGAGTCAAATGGCTCTCCTCAAGCGTATTCAACAAGGGGCTGAAGGATGCCCAGAAGGTACCCC
ATTGTATGGGATCTGATCTGGGGCCTCGGTGCACATGCTTTACATGTGTTTAGTCGAGGTTAAAAAAAC
GTCTAGGCCCCCCGAACCACGGGGACGTGGTTTTCCTTTGAAAAACACGATGATAAGCTTGCCACAACC
CGGGATCGCCACCATGGTCTTCACACTCGAAGATTTCGTTGGGGACTGGCGACAGACAGCCGGCTACAA
CCTGGACCAAGTCCTTGAACAGGGAGGTGTGTCCAGTTTGTTTCAGAATCTCGGGGTGTCCGTAACTC
CGATCCAAAGGATTGTCCTGAGCGGTGAAAATGGGCTGAAGATCGACATCCATGTCATCATCCCGTATG
AAGGTCTGAGCGGCGACCAAATGGGCCAGATCGAAAAAATTTTTAAGGTGGTGTACCCTGTGGATGAT
CATCACTTTAAGGTGATCCTGCACTATGGCACACTGGTAATCGACGGGGTTACGCCGAACATGATCGAC
TATTTCGGACGGCCGTATGAAGGCATCGCCGTGTTCGACGGCAAAAAGATCACTGTAACAGGGACCCTG
TGGAACGGCAACAAAATTATCGACGAGCGCCTGATCAACCCCGACGGCTCCCTGCTGTTCCGAGTAACC
ATCAACGGAGTGACCGGCTGGCGGCTGTGCGAACGCATTCTGGCGTAATTCTAGAAAAAAAAAAAAAA
AAAAAAAAAAAAAAA

* The IRES region is highlighted in green capitals.
* The NanoLuc-coding region is highlighted in orange capitals.

IVT Product IRES-NanoLuc-mRNA

GGCGCAUAUUAAGGUGACGCGUAAUUCACGCGUCGAGCAUGCAUCUAGGGCGGCCAAUUCCGCCCCU
CUCCCCCCCCCCCCUCUCCCUCCCCCCCCCCUAACGUUACUGGCCGAAGCCGCUUGGAAUAAGGCCGG
UGUGCGUUUGUCUAUAUGUUAUUUUCCACCAUAUUGCCGUCUUUUGGCAAUGUGAGGGCCCGGAAA
CCUGGCCCUGUCUUCUUGACGAGCAUUCCUAGGGGUCUUUCCCCUCUCGCCAAAGGAAUGCAAGGUC
UGUUGAAUGUCGUGAAGGAAGCAGUUCCUCUGGAAGCUUCUUGAAGACAAACAACGUCUGUAGCGA
CCCUUUGCAGGCAGCGGAACCCCCCACCUGGCGACAGGUGCCUCUGCGGCCAAAAGCCACGUGUAUA
AGAUACACCUGCAAAGGCGGCACAACCCCAGUGCCACGUUGUGAGUUGGAUAGUUGUGGAAAGAGU
CAAAUGGCUCUCCUCAAGCGUAUUCAACAAGGGGCUGAAGGAUGCCCAGAAGGUACCCCAUUGUAUG
GGAUCUGAUCUGGGGCCUCGGUGCACAUGCUUUACAUGUGUUUAGUCGAGGUUAAAAAAACGUCUA
GGCCCCCCGAACCACGGGGACGUGGUUUUCCUUUGAAAAACACGAUGAUAAGCUUGCCACAACCCGG
GAUCGCCACCAUGGUCUUCACACUCGAAGAUUUCGUUGGGGACUGGCGACAGACAGCCGGCUACAAC
CUGGACCAAGUCCUUGAACAGGGAGGUGUGUCCAGUUUGUUUCAGAAUCUCGGGGUGUCCGUAACU
CCGAUCCAAAGGAUUGUCCUGAGCGGUGAAAAUGGGCUGAAGAUCGACAUCCAUGUCAUCAUCCCG
UAUGAAGGUCUGAGCGGCGACCAAAUGGGCCAGAUCGAAAAAAUUUUUAAGGUGGUGUACCCUGUG
GAUGAUCAUCACUUUAAGGUGAUCCUGCACUAUGGCACACUGGUAAUCGACGGGGUUACGCCGAACA
UGAUCGACUAUUUCGGACGGCCGUAUGAAGGCAUCGCCGUGUUCGACGGCAAAAAGAUCACUGUAAC
AGGGACCCUGUGGAACGGCAACAAAAUUAUCGACGAGCGCCUGAUCAACCCCGACGGCUCCCUGCUG
UUCCGAGUAACCAUCAACGGAGUGACCGGCUGGCGGCUGUGCGAACGCAUUCUGGCGUAAUUCUAGA
AAAAAAAAAAAAAAAAAAAAAAAAAAAAAA

* The IRES region is highlighted in green capitals.
* The NanoLuc-coding region is highlighted in orange capitals.

Splint DNA for circularization of RNA

5’-ACCTTAATATGCGCCTTTTTTTTTTTTTTT-3’

11. References

1. Inagaki, M., Abe, N., Li, Z.M., Nakashima, Y., Acharyya, S., Ogawa, K., Kawaguchi, D., Hiraoka, H., Banno, A., Meng, Z.Y. *et al.* (2023) Cap analogs with a hydrophobic photocleavable tag enable

facile purification of fully capped mRNA with various cap structures. *Nat Commun*, **14**, 2657.

2. Litosh, V.A., Wu, W.D., Stupi, B.P., Wang, J.C., Morris, S.E., Hersh, M.N. and Metzker, M.L. (2011) Improved nucleotide selectivity and termination of 3'-OH unblocked reversible terminators by molecular tuning of 2-nitrobenzyl alkylated HOMedU triphosphates. *Nucleic Acids Res*, **39**, e39.
3. Gray, G.M. and Macfarlane, M.G. (1961) Composition of Phospholipids of Rabbit, Pigeon and Trout Muscle and Various Pig Tissues. *Biochem J*, **81**, 480-488.
4. Senthilvelan, A., Vonderfecht, T., Shanmugasundaram, M., Pal, I., Potter, J. and Kore, A.R. (2021) Trinucleotide Cap Analogue Bearing a Locked Nucleic Acid Moiety: Synthesis, mRNA Modification, and Translation for Therapeutic Applications. *Org Lett*, **23**, 4133-4136.
5. Abe, N., Imaeda, A., Inagaki, M., Li, Z., Kawaguchi, D., Onda, K., Nakashima, Y., Uchida, S., Hashiya, F., Kimura, Y. *et al.* (2022) Complete Chemical Synthesis of Minimal Messenger RNA by Efficient Chemical Capping Reaction. *ACS Chem Biol*, **17**, 1308-1314.
6. Edler, M., Mayrbrugger, S., Fian, A., Trimmel, G., Radl, S., Kern, W. and Griesser, T. (2013) Wavelength selective refractive index modulation in a ROMP derived polymer bearing phenyl- and -nitrobenzyl ester groups. *J Mater Chem C*, **1**, 3931-3938.
7. Gaplovsky, M., Il'ichev, Y.V., Kamdzhilov, Y., Kombarova, S.V., Mac, M., Schwörer, M.A. and Wirz, J. (2005) Photochemical reaction mechanisms of 2-nitrobenzyl compounds:: 2-Nitrobenzyl alcohols form 2-nitroso hydrates by dual proton transfer. *Photoch Photobio Sci*, **4**, 33-42.
8. Wasmuth, C.R., Hutcherson, R. and Edwards, C. (1964) Participation of So2-Radical Ion in Reduction of P-Nitrophenol by Sodium Dithionite. *J Phys Chem*, **68**, 423-425.
